# Enalapril Influence on Arterial Stiffness in Rheumatoid Arthritis Women: A Randomized Clinical Trial

**DOI:** 10.3389/fmed.2019.00341

**Published:** 2020-01-29

**Authors:** Felipe Perez-Vazquez, Magnus Bäck, Efrain Chavarria-Avila, Eduardo Gomez-Bañuelos, Carlos G. Ramos-Becerra, Óscar Pizano-Martínez, Mario Salazar-Páramo, Fernando Grover-Páez, Arnulfo H. Nava-Zavala, Ernesto G. Cardona-Muñoz, David Cardona-Müller, Sergio Duran-Barragán, Valeria N. Mera-Riofrio, Natalia Prado-Bachega, Monica Vazquez-Del Mercado

**Affiliations:** ^1^Instituto de Investigación en Reumatología y del Sistema Músculo Esquelético, Departamento de Biología Molecular y Genómica, Centro Universitario de Ciencias de la Salud, Universidad de Guadalajara, Guadalajara, Mexico; ^2^Department of Cardiology and Center for Molecular Medicine, Karolinska University Hospital, Stockholm, Sweden; ^3^Departamento de Disciplinas Filosofico, Metodologicas e Instrumentales, Centro Universitario de Ciencias de la Salud, Universidad de Guadalajara, Guadalajara, Mexico; ^4^Servicio de Reumatología PNPC 004086 CONACyT, Hospital Civil Dr. Juan I. Menchaca, Guadalajara, Mexico; ^5^Instituto de Terapéutica Experimental y Clínica, Departamento de Fisiología, Centro Universitario de Ciencias de la Salud, Universidad de Guadalajara, Guadalajara, Mexico; ^6^Departamento de Fisiología, Centro Universitario de Ciencias de la Salud, Universidad de Guadalajara, Guadalajara, Mexico; ^7^Centro Médico Nacional de Occidente, Instituto Mexicano del Seguro Social, Guadalajara, Mexico; ^8^Hospital General de Occidente, Secretaría de Salud, Zapopan, Mexico; ^9^UDG-CA-703, Inmunología y Reumatología, Centro Universitario de Ciencias de la Salud, Universidad de Guadalajara, Guadalajara, Mexico

**Keywords:** rheumatoid arthritis, arterial stiffness, ACEi, enalapril, CAVI

## Abstract

**Introduction:** Cardiovascular parameters disruption can be found in patients at early stages of rheumatoid arthritis (RA). The primary endpoint of this study was the reduction of arterial stiffness in RA patients without traditional cardiovascular risk factors or previous comorbidities, measured by cardio-ankle vascular index (CAVI) through the enalapril intervention. The secondary endpoints were the enalapril influence on carotid femoral pulse wave velocity (cfPWV), carotid intima media thickness (cIMT), carotid artery distensibility (cDistensibility), Young's incremental elastic modulus (Einc)].

**Materials and Methods:** Fifty-three patients were enrolled in a clinical, randomized, closed-label trial. The subjects were randomly assigned into two groups: One receiving 5 mg of enalapril (27) or placebo (26), both twice a day. The drug was acquired at Victory Enterprises®. The placebo was kindly provided by the Universidad de Guadalajara (UdeG), as well as the blinding into two groups: A and B. Enalapril and placebo were packed into bottles without labeling. Clinical assessment included a structured questionnaire to gather demographic and clinical variables as well as determination of CAVI, cfPWV, cIMT, carotid artery distensibility and Einc. The whole set of evaluations were analyzed at the baseline and at the end of 12 weeks of intervention.

**Results:** The CAVI measurement at baseline was 7.1 ± 1.4 and increased up to 7.5 ± 1.2 at the end of 12 weeks. Meanwhile, the enalapril group was as follows: 7.4 ± 1.2 and at the of intervention, reduced to 7.1 ± 0.9. A reduction in delta CAVI of 0.21 in the enalapril intervention group was found. In contrast, an increase of 0.39 was observed in the placebo group. The delta CAVI reduction was not influenced by age or peripheral systolic blood pressure (pSBP).

**Discussion:** Enalapril seems to be effective in CAVI reduction in RA patients. The effect of enalapril intervention on arterial stiffness translated to the clinical context might be interpreted as a reduction of 6.4 years of arterial aging.

**Trial Registration:** The protocol was approved by the Institutional Review Board with the register CI-0117 from UdeG, and 0211/18 from Hospital Civil “Dr. Juan I. Menchaca”, Secretaría de Salud Jalisco: DGSP/DDI/D.INV.28/18 and retrospectively registered at ClinicalTrials.gov Protocol Registration and Results System: NCT03667131.

## Introduction

The leading cause of death in rheumatoid arthritis (RA) is cardiovascular disease (CVD) causing an important reduction of life expectancy (1–3). The increased CVD risk develops after RA onset (4), and subclinical signs of CVD are common in RA patients (5, 6). For example, arterial stiffness (7, 8) results from changes in elasticity and functional compliance in the arterial vascular network (2) and is one of the main indicators of early CVD in asymptomatic individuals (9). We recently reported that RA disease duration predicted arterial stiffness, indicating that each year lived with the chronic inflammation of RA contributes more to vascular stiffening and CVD risk compared with a year of life without RA (10). Current evidence to reduce arterial stiffness in RA is scarce (11, 12). In this context, CVD risk stratification guidelines specific for RA is a pending task (13).

The aim of this study was to establish if an angiotensin-converting enzyme inhibitor (ACEi) could improve arterial stiffness in RA. The choice of ACEi was made based on its possible effect on arterial stiffness reduction and its feasibility (oral administration, adequate tolerance, affordable). A randomized double blind placebo-controlled pilot study was carried out in female RA patients without comorbidities to evaluate the effects of ACEi on different cardiovascular parameters. Arterial stiffness was evaluated by cardio-ankle vascular index (CAVI) (14), which is based on the stiffness index beta (15, 16) and less affected by blood pressure (BP) compared with PWV according to the algorithm (17) and previous clinical studies (18).

## Materials and Methods

### Participants

We included patients classified as RA according to the American College of Rheumatology/European League Against Rheumatism (ACR/EULAR) 2010 criteria (19) attending to the rheumatology outpatient clinic at the OPD Hospital Civil “Dr. Juan I. Menchaca,” Guadalajara, Jalisco, México. The treatment regimen of the patients was registered. Inclusion criteria were: age > 18 to < 80 years old; female gender; no history of CVD, diabetes mellitus, high blood pressure (HT), thyroid, renal or hepatic disease. All subjects identified themselves with a Federal ID and gave their written consent before enrollment. Supporting information at: https://figshare.com/s/02dc51e5c796feaceff3. Exclusion criteria were current pregnancy, glucocorticoid use > 10 mg per day or equivalent, statin intake; nicotine use, alcohol use; adherence to treatment intervention <80%, and serious adverse effects secondary to enalapril intake (angioedema, orthostatic hypotension, cough, dizziness, syncope, muscle cramps, or diarrhea). From a total of 83 patients invited, 59 individuals met the inclusion criteria and were randomly assigned as enalapril treatment (30 cases) and 29 into placebo group.

### Ethics Approval and Consent to Participate

The protocol was approved by the Institutional Review Board (IRB) with the register CI-0117 from Centro Universitario de Ciencias de la Salud of the Universidad de Guadalajara (UdeG), 0211/18 from Hospital Civil “Dr. Juan I. Menchaca,” Secretaría de Salud Jalisco: DGSP/DDI/D.INV.28/18 and retrospectively registered at ClinicalTrials.gov Protocol Registration and Results System: NCT03667131. This protocol can be accessed at https://figshare.com/s/02dc51e5c796feaceff3 (Supplementary Data Sheets 1, 2). Research was conducted following Helsinki criteria last updated in 2013, Fortaleza, Brazil.

### Interventions

The study was carried out from February 2017 to February 2018. The subjects were randomly assigned to a group receiving 5 mg of enalapril or to a group receiving 5 mg of placebo, both twice a day. Intervention was carried out for 90 days at time zero (baseline) and after 12 weeks. The minimum level of medical adherence required was 80%.

### Objectives

The aim of this study was to determine the effect of suboptimal doses of enalapril to reduce arterial stiffness in RA patients using non-invasive cardiovascular parameters.

#### Endpoints

Primary endpoint: The primary endpoint of this study was the reduction of arterial stiffness in RA patients without traditional cardiovascular risk factors or previous comorbidities, measured by CAVI through the enalapril intervention.

The secondary endpoints were the enalapril influence on cfPWV, carotid intima media thickness (cIMT), carotid artery distensibility (cDistensibility), Young's incremental elastic modulus (Einc)].

### Sample Size

Sample size was determined with continuous response equation for parallel group clinical trials considering alfa 0.05, beta 0.80, SD 1.3 m/s, delta 1 m/s, and tracing lost of 20%, obtaining a minimum size of 26 subjects per group studied (10).

### Design

This is a parallel two-arm pilot trial, randomized and closed-label investigator-driven study (not sponsored by any pharmaceutical company).

#### Randomization-Sequence Generation

Treatment was allocated by blocked randomization. The random allocation sequence was generated by EG-B, MD, PhD using the world wide web (www) page Randomization.com (http://www.randomization.com), considering a block size of 8 and randomization ratio of 1:1. To ensure allocation concealment, the Centro Universitario de Ciencias Exactas e Ingenieria (CUCEI), UdeG prepared the study treatments in identical sequentially numbered containers labeled as treatment A and B. Felipe Perez-Vazquez MD, assigned treatment to participants in the consecutive order for enrollment, according to the random list. Felipe Perez-Vazquez MD was blinded to the block size.

#### Randomization-Allocation Concealment

**Figure 1:**
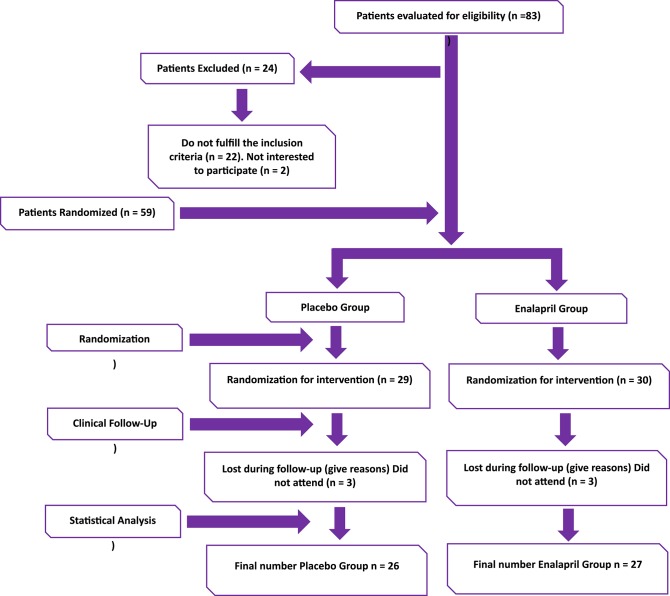
Flow chart clinical trial.

The participants were randomly assigned (with a ratio 1:1) to receive either enalapril or placebo treatment. A total of 53 subjects completed the intervention: 27 from enalapril and 26 from placebo group (Figure 1).

#### Randomization-Implementation

Felipe Perez-Vazquez MD, MSc assigned participants to each group in the consecutive order for enrolment, according to the random list.

#### Blinding

The drug was acquired at Victory Enterprises® S. A. de C. V. The placebo treatment and blinding procedure were kindly provided by the CUCEI, UdeG. Patients, and personnel conducting data collection and arterial stiffness evaluations were blinded to the allocation status of the study participants. The blinded groups were named as A and B. Enalapril and placebo were packed into bottles without labeling. The blinding was opened until the end of the intervention period and analysis results in front of all the coauthors of the study.

#### Enalapril Adverse Reactions

In agreement with the Official Mexican Standard NOM-220-SSA1-2016, Installation and operation of pharmaco-surveillance, all patients were monitored for the possible presence of adverse events, the characteristics of these events and the importance of reporting any symptoms during the study. Information was given about emergency phone numbers of the responsible researchers in case of suspected adverse events.

For this purpose, specific questionnaires for the registration of any event associated with the intervention were supplied to the patients. For the report of adverse events, the Naranjo score was contemplated (20).

### Statistical Methods

Variables were assessed for normality using the Kolmogorov-Smirnov test. In this study, the variables studied were divided into qualitative and quantitative. Qualitative variables were represented in frequencies and percentages (%). Quantitative variables were divided into parametric or non-parametric. If the variable was found to be quantitative parametric, we represented it as a mean ± standard deviation (SD); if it was quantitative non-parametric, we represented it as a median and interquartile range, from quartile 1 (25%) to quartile 3 (75%). Comparisons were made using one-way ANOVA and Kruskall-Wallis tests followed by Tukey and Mann-Whitney U *post hoc* tests as applicable. Chi-square, Pearson and Spearman correlations coefficients were also calculated, as appropriate. An ANCOVA analysis was carried out with backward mode and a *P* = 0.05 for entrance and *P* = 0.10 for elimination. All data were analyzed using SPSS 24.0 software (SPSS Inc. Chicago, IL) and GraphPad Prism version 6.00 for Windows (GraphPad Software, La Jolla, CA), considering a two-tailed level of *P* < 0.05 to be statistically significant for analysis.

### Clinical Assessment

A structured questionnaire to gather demographic and clinical variables, including disease duration and treatment, was applied to each patient. RA disease activity indexes: Disease Activity Score on 28 joints (DAS28), Simple Disease Activity Index (SDAI) and Clinical Disease Activity Index (CDAI) were applied to all patients during the time of intervention (baseline and at the end of 12 weeks), selected anthropometric measurements, biochemical and cardiovascular parameters (cfPWV, CAVI, cIMT, cDistensibility, Einc) were determined for both groups.

#### Laboratory Measurements

Venous blood samples were collected at the moment of clinical assessment after an overnight fasting. Sera were stored at −70°C until used. αCCP was determine by ELISA (Axis-Shield Diagnostics Ltd., Dundee, Scotland), Erythrocyte sedimentation rate (ESR) was measured using Wintrobe's method (21). C-reactive protein (CRP) by nephelometry. Total cholesterol (TC), triglycerides (TG), high-density lipoprotein cholesterol (HDL-c) and low-density lipoprotein cholesterol (LDL-c) were determined by standard techniques.

#### Arterial Stiffness Measurement

Briefly, the cfPWV was measured by tonometry using the Pulse Pen device (DiaTecne s.r.l., Milan, Italy) in meters/second (m/s) (10). Cardio-ankle vascular index. CAVI was performed using the VaSera VS-1000 device (Fukuda Denshi Co., Ltd. 2-35-8 Hongo, Bunkyo-ku, Tokyo, 113-8420, Japan). Carotid ultrasound examination. As described elsewhere, carotid examination was done by doppler ultrasound (MyLabOne, Esaote, Firenze, Italy) using a software guidance (22). cIMT was tracked by radiofrequency and an automated software (22). Carotid artery distensibility (cDistensibility) was evaluated by an automated software using radiofrequency (22). Einc modulus, also named longitudinal elasticity module; is a parameter that evaluates the elastic properties of the arterial wall, but not influenced by vessel anatomy. Einc was calculated as follows: [3(1+Luminal Cross-Sectional Area (LCSA)/Wall Cross-Sectional Area (WCSA))]/Distensibility, where LSCA is a function of BP (23). The cardiovascular disease risk score version 3 (QRISK3)-2018 % (10-year QRISK3 score) and QRISK3-2018 RR: (QRISK3 relative risk) were obtained using the calculator available online (https://qrisk.org/three/) (24).

## Results

### Clinical Characteristics of the Study Groups at Baseline and After 12 Weeks of Intervention

From a total of 83 RA patients (Figure 1), 59 met the inclusion criteria. After randomization 29 were allocated to the placebo group and 30 to receive enalapril. In both groups 3 subjects were lost during follow-up. 26 subjects from the placebo group and 27 from the enalapril completed the trial and were included in the final analysis. Table 1 shows the characteristics of the study groups. At baseline and at the end of the intervention, there were no significant differences in age, disease activity, autoantibodies frequency, lipid profile or QRISK score and pharmacological treatments. RA patients were treated with conventional synthetic disease-modifying anti-rheumatic drugs (csDMARDs) such as methotrexate, chloroquine and sulfasalazine.

**Table 1:** Clinical characteristics of the RA study groups at baseline and after 12 weeks of intervention.

	**Baseline**			**12 weeks**		
	**Placebo**	**Enalapril**	***P***	**Placebo**	**Enalapril**	***P***
	***n* = 26**	***n* = 27**		***n* = 26**	***n* = 27**	
Age, years	49 ± 11	49 ± 10	0.89	49 ± 11	50 ± 10	0.89
BMI, kg/m^2^	28.1 ± 4.2	28.2 ± 3.7	0.94	28.0 ± 4.3	28.1 ± 4.1	0.97
Fat mass, %	36.1 ± 5.6	37.4 ± 6.1	0.44	35.7 ± 5.5	36.9 ± 5.0	0.41
TC, mg/dL	210.7 ± 40.9	198.6 ± 38.3	0.27	203.9 ± 40.2	210.7 ± 47.9	0.58
TG, mg/dL	143.4 ± 52.6	148.5 ± 70.2	0.77	152.5 ± 57.9	156.2 ± 101.9	0.87
HDL-c, mg/dL	46.1 ± 10.0	46.7 ± 11.5	0.85	44.8 ± 11.1	48.3 ± 11.4	0.26
LDL-c, mg/dL	124.9 ± 63.1	105.4 ± 52.5	0.23	86.2 ± 17.3	94.3 ± 46.8	0.41
Glucose, mg/dL	90.5 ± 6.8	91.1 ± 11.9	0.83	84.6 ± 9.9	89.0 ± 9.8	0.11
Disease duration, years	5.1 (3.7–6.8)	5.6 (2.7–0.8)	0.89	5.3 (3.9–7.1)	5.9 (3.0–12.0)	0.92
RF (+), *n* (%)	12 (50)	17 (63)	0.41	12 (50)	17 (63)	0.41
αCCP (+), *n* (%)	17 (65)	16 (59)	1.00	17 (65)	16 (59)	1.00
CRP, mg/L	5.8 (3.2–11.7)	6.9 (3.7–11.6)	0.55	6.4 (2.0–10.6)	6.4 (3.8–11.7)	0.47
ESR, mm/h	20.2 ± 13.2	24.0 ± 12.2	0.28	20.9 ± 12.1	22.4 ± 11.9	0.64
DAS-28 CRP	3.3 ± 1.6	3.3 ± 1.4	0.93	2.8 ± 1.3	3.2 ± 1.1	0.18
CDAI	8.4 (1.9–26.2)	5.4 (3.0–15.1)	0.78	4.9 (1.8–11.2)	5.5 (3.9–13.8)	0.17
SDAI	18.3 (7.3–31.0)	12.5 (8.2–31.0)	0.99	10.5 (5.8–24.9)	13.6 (9.7–23.1)	0.29
QRISK3-2018, %	1.9 (1.1–4.8)	1.8 (1.0–4.0)	0.69	2.1 (1.1–4.7)	2.0 (1.0–4.2)	0.63
QRISK3-2018, RR	1.2 (1.1–1.6)	1.2 (1.1–1.4)	0.34	1.3 (1.1–1.6)	1.2 (1.0–1.3)	0.10

*BMI, Body mass index; TC, total cholesterol; TG, triglycerides; HDL-c, High density lipoprotein cholesterol; LDL-c, Low-density lipoprotein cholesterol; RF, rheumatoid factor; αCCP, anti-cyclic citrullinated peptide antibodies; CRP, c-reactive protein; ESR, Erythrocyte sedimentation rate; CDAI, Clinical disease activity index; SDAI, Simple disease activity index; DAS-28, Disease activity score on 28 joints; QRISK3, cardiovascular disease risk score version 3; QRISK3-2018%, 10-year QRISK3 score; QRISK3-2018RR, QRISK3 relative risk*.

### Monitoring of Adverse Reactions to Enalapril

We did not report cough as an adverse reaction or any other symptom during the intervention period. In one case, a patient reported flu symptoms and experienced cough during 5 days. This event was registered at 45 days after the beginning of the study. However, at the end of the intervention period, we noticed that this patient was included on the placebo group.

### Enalapril Reduced the Cardio-Ankle Vascular Index (CAVI) in RA Subjects After 12 Weeks of Intervention

**Figure 2:**
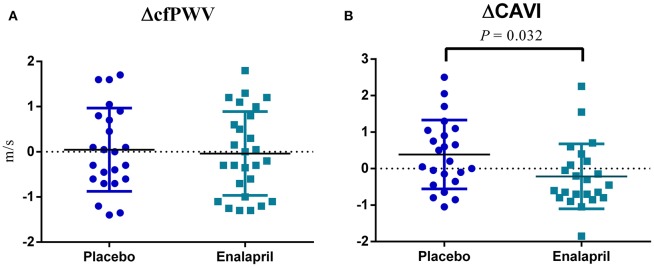
Comparison of 1cfPWV and 1CAVI. **(A)** The difference (Δ) of meters per second (m/s) in carotid-femoral pulse wave velocity (cfPWV) after 12 weeks between placebo and enalapril groups. **(B)** The difference (Δ) in cardio-ankle vascular index (CAVI) after 12 weeks between placebo and enalapril groups.

The comparison of blood pressure, cfPWV and CAVI evaluations at baseline and at 12 weeks are shown on Table 2. Subjects with RA showed a significant reduction in SBP after 12 weeks of treatment with enalapril. Regarding arterial stiffness, there was no significant difference in the mean cfPWV in both groups after 12 weeks of intervention. Nonetheless, a trend for lower CAVI in the enalapril group after 12 weeks of intervention was observed, although this difference was not statistically significant (Table 2). Next, the effect size of the intervention (25) was evaluated by addressing the difference in cfPWV and CAVI (ΔcfPWV and ΔCAVI) between the baseline and after 12 weeks of treatment (Supplementary Table 1). ΔCAVI was statistically significant between the placebo and enalapril groups (Figure 2B). On the other hand, there was no significant difference in ΔcfPWV (Figure 2A).

**Table 2:** Cardiovascular parameters of the RA study groups at baseline and after 12 weeks of intervention.

	**Baseline**			**12 weeks**		
	**Placebo**	**Enalapril**	***P***	**Placebo**	**Enalapril**	***P***
pSBP, mmHg	114 ± 8.7	111.9 ± 10.8	0.51	111 ± 9.5	105.9 ± 5.8	0.02
pDBP, mmHg	69 ± 6.8	70 ± 7.3	0.67	68 ± 6.8	68 ± 4.5	0.91
cIMTmean, μm	626.3 ± 119.8	621.6 ± 68.7	0.86	633.8 ± 135.3	622.9 ± 103.6	0.74
cDistensibility, 10^−3^/ kPa	24.6 ± 9.4	23.4 ± 8.6	0.62	24.7 ± 7.9	25.9 ± 10.1	0.63
Einc, kPax10^3^	0.48 (0.4–0.7)	0.61 (0.4–0.7)	0.67	0.57 (0.4–0.8)	0.49 (0.4–0.6)	0.89
cfPWV, m/s	7.4 ± 1.0	7.5 ± 1.0	0.70	7.4 ± 1.5	7.4 ± 1.7	0.94
CAVI	7.1 ± 1.4	7.4 ± 1.2	0.52	7.5 ± 1.2	7.1 ± 0.9	0.27

*pSBP, peripheral systolic blood pressure; pDBP, peripheral diastolic blood pressure; cIMT, Carotid intima-media thickness; cDistensibility, Carotid artery distensibility; Einc, Young's Elastic modulus; cfPWV, carotid to femoral pulse wave velocity; CAVI, Cardio-Ankle Vascular Index. Values were representend as mean ± SD and median with ranges. Comparisons were done with the Student's T test and Mann-Whitney U for dependent samples*.

### Predictive Value of Intervention With Enalapril and Age Decade on CAVI in RA Patients

To further assess which factors could influence the response to enalapril in RA subjects, we evaluated the correlation between clinical characteristics and pharmacological treatments at baseline and 12 weeks with ΔCAVI (Supplementary Table 2). Except from age, which significantly correlated with CAVI at baseline and 12 weeks, no significant correlations were established.

An ANCOVA to assess the impact of age decade on CAVI (Model 1) showed no influence at baseline (Table 3 Model 1, Supplementary Figure 1A). On the other hand, the effect of enalapril intervention on arterial stiffness translated to a clinical context, might be interpreted as a diminish of 7.0% in total CAVI which could represents a reduction of 6.4 years of arterial aging (Table 3, Model 2, Supplementary Figure 1B).

**Table 3:** Multiple linear regression analysis for CAVI.

**Model 1^#^ Dependent variable: CAVI Baseline**			
	***R***^****2****^ **=** **0.333**		
	****β****	**95% CI**	***P***
Constant	5.995	(5.630 to 6.780)	<0.001
Age Group (0 when <40, 1 when 40 to <50, 2 when 50 to <60, 3 when >60)	0.746	(0.451 to 1.094)	<0.001
Study groups (1 when placebo, 2 when enalapril)	0.136	(−0.523 to 0.795)	0.679
**Model 2^##^ Dependent variable: CAVI After 12 weeks of intervention**			
	***R***^**2**^ **=** **0.522**		
	****β****	**95% CI**	***P***
Constant	6.982	(6.204 to 7.761)	<0.001
Age Group (0 when <40, 1 when 40 to <50, 2 when 50 to <60, 3 when >60)	0.762	(0.531 to 0.993)	<0.001
Study groups (1 when placebo, 2 when enalapril)	−0.488	(−0.950 to −0.026)	0.039
**Model 3^###^ Dependent variable:** **Δ** **CAVI**			
	***R***^**2**^ **=** **0.103**		
	****β****	**95% CI**	***P***
Constant	0.958	(0.028 to 1.887)	0.044
Age Group (0 when <40, 1 when 40 to <50, 2 when 50 to <60, 3 when >60)	0.022	(−0.254 to 0.299)	0.872
Study groups (1 when placebo, 2 when enalapril)	−0.610	(−1.162 to 0.058)	0.031

*β—Coefficients are given as value (95% confidence Interval); unit for β—Coefficients is index from CAVI. The models were adjusted by peripheral Systolic blood pressure (pSBP), the method backward was used with a P_IN_ = 0.05 and a P_OUT_ = 0.10*.

# *Excluded variables: study group, log-αCCP, BMI, DAS-28 CRP, ESR, HDL-c, SDAI, TC, TG, and the pharmacological treatments: methotrexate, chloroquine and sulfasalazine*.

## *Excluded variables: log-αCCP, BMI, DAS-28 CRP, ESR, HDL-c, SDAI, TC, TG, and the pharmacological treatments: methotrexate, chloroquine and sulfasalazine*.

### *Exclude variables: study group, age group, log-αCCP, BMI, DAS-28 CRP, ESR, HDL-c, SDAI, TC, TG and the pharmacological treatments: methotrexate, chloroquine and sulfasalazine*.

*log-αCCP: log_10_ of titer anti-cyclic citrullinated peptide antibodies; BMI: Body mass index; CDAI: Clinical disease activity index; CRP: c-reactive protein; DAS-28 CRP: Disease activity score on 28 joints by CRP; Disease duration; ESR: Erythrocyte sedimentation rate; HDL-c: High density lipoprotein cholesterol; SDAI: Simple disease activity index; TC: Total cholesterol and TG: Triglycerides*.

### Delta CAVI of Enalapril Effect on Life Decades and Comparison of CAVI From Enalapril Group With a Reference Value

Although the enalapril-induced effect on ΔCAVI in the context of life decades was not significant in the ANCOVA analysis (Table 3, Model 3), a marginal means plot revealed an important reduction on ΔCAVI from 40 to 60 years old. In contrast, a less apparent improvement was registered in patients younger than 40 and older than 60 years old (Supplementary Figure 2). In order to look for a possible explanation of less apparent improvement in RA patients younger than 40 and older than 60 years old; we included CAVI values from a reference population in a graphic to compare the enalapril effect on arterial aging (25) (Supplementary Figure 3). This comparison indicated that RA patients younger than 40 years old could not improve more, since their arterial aging is close to the reference population CAVI values (25).

## Discussion

Currently, RA is significantly associated with subclinical atherosclerosis and in turn, with an increased CV risk (26). In addition, RA patients have subclinical increase in aortic stiffness and positive aCCP, suggesting a possible role of these autoantibodies in the accelerated atherosclerosis process (27). In addition, the role of autoantibodies such as rheumatoid factor and anti-CCP in other studies is controversial, but some authors pointed out the possible association of RF in CVD risk development (28). The above suggests that patients with RA may benefit from a meticulous screening for CV risk factors, early arterial stiffness evaluation and more specific CV prevention strategies, not biased by the normal aging arterial process (26, 29).

Current evidence of intervention trials to reduce arterial stiffness in RA is however scarce. Vitamin D serum levels were positive associated with CD34+ circulating cells but failed to show correlation with cIMT and PWV in RA naïve patients, moderate active and without comorbidities (30).

Our study addressed the use of suboptimal doses of enalapril for the reduction of arterial stiffness in female RA patients without known CVD. Clinical trials on patients with hypertension showed that while perindopril (10 mg/day), enalapril (20 mg/day), losartan (100 mg/day), and telmisartan (80 mg/day) decrease SBP, only perindopril and telmisartan reduced the arterial stiffness evaluated by PWV (31). However, is important to note that PWV is largely dependent on BP, which therefore becomes an important confounder when evaluating the effect of anti-hypertensive drugs on arterial stiffness. In contrast, the use of CAVI could be a better tool since is less dependent of BP (17).

In the present study, the enalapril and placebo groups did not differ in terms of age, anthropometric measurements, metabolic, inflammation, and cardiovascular parameters, indicating that the groups were in ideal conditions to be compared without bias. After 12 weeks of enalapril intervention, a reduction in BP was observed compared with the placebo group. In contrast, none of the cardiovascular non-invasive measurements were significantly different between the groups.

However, when evaluating differences between groups at baseline and after 12 weeks of intervention, a reduction in CAVI of 0.21 in the enalapril intervention group was observed whereas an increase of 0.39 was registered in the placebo group. This difference was significantly different when comparing the two groups, indicating a reduced delta-CAVI in the enalapril-group compared with the placebo-group. This reduction was independent of the changes in BP induced by enalapril indicating that the improvement of CAVI was not a result of BP lowering.

In addition to BP, age is a possible confounder in studies of arterial stiffness. One could argue that arterial aging in RA placebo group was as expected, resulting from the chronic inflammation state in RA. Several arguments indeed suggest that arterial stiffness is a direct result of aging (32, 33). Phenomena such as elastin fiber deterioration, calcification, glycoxidation, and lipid peroxidation within the arterial wall might be responsible for arterial aging (34). The observed positive relationship between arterial stiffness and RA has been reported by our group (10). Further analyses were performed to dissociate the enalapril-induced effect on arterial aging evaluated by CAVI with that from age itself. These analyses revealed that a younger subject, whose CAVI was close to a reference value for their age, did not improve further on treatment. Likewise, older subjects did not exhibit an improvement in CAVI. These results suggest that there may be an optimal age span for initiating CVD preventive treatment in RA. The improvement in CAVI by enalapril in RA patients in the multivariate analyses was 0.488, which was equivalent to a reduction of 7.0% in total CAVI. This could be interpreted as a reduction of 6.4 years in arterial aging in the enalapril-treated group. Taken together, one of the main findings of this clinical trial was the reduction of CAVI after 3 months of enalapril treatment in suboptimal dose.

We did not observe adverse effects secondary to enalapril treatment, as described in Material and Methods and Results; we were monitoring all patients during the intervention period. Previous studies described cough secondary to the use of enalapril as an adverse reaction on 15% of the cases (35). We consider that in this study the use of low dose of enalapril diminished the chances for developing adverse effects.

One strength of our study, is the delta calculation of the cardiovascular parameters, since this allow us to analyze the outcome on arterial stiffness through enalapril use (25). Even when we were able to demonstrate the amelioration of arterial stiffness with CAVI, it is important to highlight that this finding do not mean that the rest of cardiovascular parameters evaluated in this clinical trial including the gold standard (cfPWV), are not useful for the measurement of arterial stiffness.

We acknowledge that this study has certain limitations. The short period of time (12 weeks) evaluated may not have been enough to observe changes in the rest of cardiovascular parameters explored: cIMT, cDistensibility, Einc, PWV (36). Even 12 weeks of intervention period is a short period of time, we were able to register an improvement in CAVI measurement, since the device is less influenced by peripheral blood pressure and less dependent from the observer. cIMT is more related to aging process and peripheral blood pressure than CAVI. It is reported that cIMT increase in about 100 μm per year of life in general population (37). On the other hand, Einc modulus is an algorithm recently described. Our RA patients treated with enalapril improved at the end of the intervention period in −0.08 vs. to 0.01 in the placebo group but not enough to achieve statistical significance. Since cDistensibility is also an algorithm related to: cIMT, PWV and peripheral blood pressure it applies the same explanation for the absence of positive results on cIMT. A caveat in our study was the lack of inter and intra-observer variability coefficients. Notwithstanding, the reproducitibility of the device used for arterial stiffness measurement (VaSera) has shown high reproducibility in other studies (38).

It is also important to point out that only female RA patients without comorbidities were included and that the applicability of our findings to larger non-selected RA populations remains to be established. Finally, the small sample size of the present study is an obvious limitation of the study. More studies with longer periods of intervention and larger sample size are needed to confirm and replicate our results using non-invasive cardiovascular measurements including the gold standard.

The use of conventional synthetic disease-modifying anti-rheumatic drugs (csDMRADs) did not influence or decrease the arterial stiffness measurement. We described this information on Table 3 and Supplementary Table 2.

Notwithstanding, the clinical relevance of this trial relies on the proposal itself offering a low cost, common used, affordable drug even in low economic income population, which offers the possibility to include a strategy to reduce arterial stiffness in RA patients. Even this trial needs to be reproducible with larger samples of patients, longer periods of treatment etc, it is worthy to support early treatment options to prevent cardiovascular comorbidities beyond the treat to target (T2T) strategy, which is focused so far to achieve clinical remission of RA disease activity through the use of disease modifying anti-rheumatic drugs (DMARDs).

In summary, suboptimal doses of enalapril (10 mg daily), was successful to reach a CAVI improvement, independent of age and other confounders according to ANCOVA analysis. Enalapril treatment in suboptimal doses employed in this clinical trial was able to ameliorate CAVI values after 3 months. This observation translated to the clinical context might be interpreted as enalapril intervention could reduce 6.4 years arterial aging in RA patients.

We are enthusiastic that in the near future, the treatment of RA will not be focused only on the T2T strategy, which is directed to achieve as soon as possible, the remission of disease clinical activity. A message for an integral treatment including the early detection and preservation of cardiovascular health in RA patients is mandatory.

## Data Availability Statement

The datasets generated and analyzed for this study can be found in the figshare repository (https://figshare.com/s/02dc51e5c796feaceff3).

## Ethics Statement

The studies involving human participants were reviewed and approved by Comités de Ética, de Investigación y Bioseguridad register CI-0117, Centro Universitario de Ciencias de la Salud. The patients/participants provided their written informed consent to participate in this study. Register 0211/18 from Hospital Civil Dr. Juan I. Menchaca, Secretaría de Salud Jalisco: DGSP/DDI/D.INV.28/18. Research was conducted following Helsinki criteria last updated in 2013, Fortaleza, Brazil.

## Author Contributions

FP-V, MV-D, and EG-B contributed to the conceptualization of the study. FP-V, MB, EC-A, EG-B, CR-B, ÓP-M, MS-P, FG-P, AN-Z, EC-M, DC-M, SD-B, VM-R, and NP-B design the methodology of the work. FP-V, MV-D, EC-A, CR-B, ÓP-M, FG-P, AN-Z, EC-M, DC-M, SD-B, VM-R, and NP-B had an active role in the process of patients and data acquisition. FP-V, MV-D, MB, ÓP-M, MS-P, FG-P, AN-Z, and SD-B contributed to the validation of results. FP-V, MB, EC-A, and EG-B carried out the formal analysis of the data. MV-D, CR-B, EC-M, and FG-P were in charge of supply needed resources. FP-V, EC-A, EG-B, CR-B, VM-R, and NP-B worked together for data curation. FP-V, MV-D, EC-A, EG-B, ÓP-M, MS-P, AN-Z, EC-M, DC-M, SD-B, VM-R, and NP-B wrote the work's draft. FP-V, MV-D, MB, EC-A, CR-B, ÓP-M, MS-P, FG-P, AN-Z, EC-M, DC-M, and SD-B reviewed the intellectual content of the final document. MV-D, MB, CR-B, EC-M, FP-V, VM-R, and NP-B coordinated and supervised the entire project.

### Conflict of Interest

The authors declare that the research was conducted in the absence of any commercial or financial relationships that could be construed as a potential conflict of interest.

## Supplementary Material

Supplementary Data Sheet 1 Trial Protocol English language.

Supplementary Data Sheet 2 Trial Protocol Spanish language.

## Abbreviations

ACEi: Angiotensin-converting enzyme inhibitors
ACR/EULAR: American College of Rheumatology/European League Against Rheumatism
BMI: Body mass index
BP: Blood Pressure
CAVI: cardio-ankle vascular index
CDAI: Clinical Disease Activity Index
cfPWV: carotid femoral pulse wave velocity
cIMT: carotid intima media thickness
CRP: C-reactive protein
CVD: cardiovascular disease
DAS28: Disease Activity Score on 28 joints
Einc: Young's incremental elastic modulus
ESR: Erythrocyte sedimentation rate
HDL-c: High-density lipoprotein cholesterol
HT: High blood pressure
IRB: Institutional Review Board
LCSA: Luminal Cross-Sectional Area
LDL-c: low-density lipoprotein cholesterol
PBP: Peripheral blood pressure
pDBP: Peripheral diastolic blood pressure
pSBP: Peripheral systolic blood pressure
PWV: Pulse wave velocity
RA: Rheumatoid arthritis
RAAS: Renin-angiotensin-aldosterone system
RF: Rheumatoid factor
SD: Standard deviation
SDAI: Simple Disease Activity Index
TC: Total cholesterol
TG: Triglycerides
WCSA: Wall Cross-Sectional Area
Δ: Delta
αCCP: Anti-cyclic citrullinated peptide antibodies
QRISK 3: cardiovascular disease risk score version 3.
