# Supplementary material for: Enalapril Influence on Arterial Stiffness in Rheumatoid Arthritis Women: A Randomized Clinical Trial
Source: Front Med (Lausanne). 2020 Jan 29;6:341. doi: 10.3389/fmed.2019.00341 (PMC7025600; doi:10.3389/fmed.2019.00341)
Supplement: Supplementary Data Sheet 1 — Trial Protocol English language. [file Data_Sheet_1.PDF]

CI/006/2017

EVALUATION DICTUM

In the city of Guadalajara, Jalisco on 23th January 2017, in the Centro Universitario de Ciencias de la Salud of the Universidad de Guadalajara, the members of the Ethics, Research and Biosafety Committees met to decide on the evaluations issued by their peers of the project with registration number CI-0117: **"Effect of prophylactic treatment with enalapril maleate on arterial stiffness in patients with rheumatic arthritis"** of which Mónica Vázquez Del Mercado Espinosa MD, PhD is Principal Investigator.

The members of the committees agreed that according to the evaluations issued by their peers, the project presented the requirements regarding its presentation, quality and content for the **approval.**

ATTENTIVELY

Research ethics committee

Martha Villaseñor Farías PhD — President

Noe Alfaro Alfaro PhD — Secretary

Research committee

Jorge R. Padilla Gutierrez PhD — President

Andrés Palomera Chávez PhD — Secretary

Biosecurity committee

Ma. De la Luz Galván Ramírez PhD — President

Celia Guerrero Velázquez PhD — Secretary

Vo. Bo.

Adrián Daneri Navarro PhD

Research Coordinador

UNIVERSIDAD DE GUADALAJARA

NUEVO HOSPITAL CIVIL DE GUADALAJARA

"DR. JUAN I. MENCHACA"

"EFFECT OF THE USE OF ENALAPRIL ON ARTERIAL STIFFNESS IN PATIENTS WITH RHEUMATOID ARTHRITIS".

Principal Investigator: Mónica Vázquez del Mercado Espinosa, MD, PhD

Guadalajara, Jalisco, January 2017

UNIVERSIDAD DE GUADALAJARA

NUEVO HOSPITAL CIVIL DE GUADALAJARA

“DR. JUAN I. MENCHACA”

Protocol proposal

“EFFECT OF THE USE OF ENALAPRIL ON ARTERIAL STIFFNESS IN PATIENTS WITH RHEUMATOID ARTHRITIS”

Principal Investigator: Mónica Vázquez del Mercado Espinosa, MD, PhD

Guadalajara, Jalisco, January 2017

“EFFECT OF THE USE OF ENALAPRIL ON ARTERIAL STIFFNESS IN PATIENTS WITH RHEUMATOID ARTHRITIS”

Index.

Title. 6

Researchers. 6

Headquarters. 6

1. Theoretical framework. 7

1.1 Rheumatoid arthritis. 7

1.1.1 RA epidemiology. 7

1.1.2 RA etiology. 7

1.1.3 RA Physiopathology. 8

1.1.4 Morbidity and Mortality in RA. 9

1.1.5 Arterial stiffness in rheumatoid arthritis. 10

1. 2 Pulse wave velocity. 10

1.3 Enalapril maleate. 12

1.3.1 Chemical structure of enalapril maleate. 12

1.3.2 Metabolism and pharmacokinetic. 12

1.3.3 Pharmacodynamic. 13

1.3.4 Mechanism of action. 13

1.3.5 Enalapril indications. 14

1.3.6 Adverse effects. 14

1.3.7 Findings in laboratory test. 15

2. Background. 16

3. Justification. 17

4. Problem statement. 18

4.1 Research question. 18

5. Hypothesis. 19

5.1 Alternative hypothesis. 19

5.2 Null hypothesis. 19

6. Objectives. 20

6.1 General Objective. 20

6.2 Specific objectives. 20

7. Materials and methods. 21

7.1 Study design. 21
7.2 Study universe. 21
7.3 Workplace. 21
7.4 Intervention and follow-up. 21
7.4.1 Selection or assignement of study subjects to study groups. 22
8. Sample Height. 23
9. Selection criteria. 24
9.1 Inclusion criteria. 24
9.2 Non-inclusion criteria. 24
9.3 Elimination criteria. 24
10. Variables. 26
10.1 Independents. 26
10.2 Dependents. 26
10.3 Intervening. 26
10.4 Variables definition. 26
10.5 Operationalization of variables. 28
11. Study group. 30
11.1 Assignment of medication intervention and blinding. 30
12. Clinical determinations. 31
13 Laboratory determination. 34
13.1 Obtaining biological samples. 34
14. Statistical analysis. 36
15. Study description. 36
15.1 Field phase development. 36
15.2 Therapeutic adherence. 38
15.3 Segurity criteria. 38
15.4 Data collection. 38
15.5 Dissemination of the final results of the investigation. 38
16. Ethical aspects. 39
17. Financing. 40
18. Human resources. 40
18.1 Work team. 40
19. Material resources. 42

20. Conflicts of interest. 42

21. Bibliographic references. 43

22. ATTACHED. 47

22.1 ATTACHED 1. CONSENT LETTER UNDER INFORMATION 47

22.2 ATTACHED 2. DATA COLLECTION AND IDENTIFICATION SHEET. 53

22.3 ATTACHED 3. DAILY MONITORING AND PATIENT FOLLOW-UP 69

Title.

“EFFECT OF THE USE OF ENALAPRIL ON ARTERIAL STIFFNESS IN PATIENTS WITH RHEUMATOID ARTHRITIS”.

Researchers.

- **Principal Investigator.** Mónica Vázquez del Mercado Espinosa, MD, PhD
- **Research collaborator.** José de Jesús Eduardo Gómez Bañuelos, MD, PhD
- **Research collaborator.** Felipe de Jesús Pérez Vázquez, MD, MSc

Headquarters.

- **Servicio de Reumatología**, División de Medicina Interna del Nuevo Hospital Civil de Guadalajara, “Dr. Juan I. Menchaca”.
- **Instituto de Investigación en Reumatología y del Sistema Musculo-Esquelético**, Departamento de Biología Molecular, Centro Universitario de Ciencias de la Salud, Universidad de Guadalajara.

1. Theoretical framework.

1.1 Rheumatoid arthritis.

The rheumatoid arthritis (RA) is a sistemic chronic inflammatory disease [1] differentiated for chronic erosive arthritis in synovial's joints. Its main symptoms are: pain, fatigue, morning stiffness and functional disability. Its characterized for the presence of auto-antibodies like rheumatoid factor (RF) and antibodies against citrullinated peptides (ACPA) [1, 2].

1.1.1 RA epidemiology.

The RA affects to 0.5% to 1% of the world population [3, 4] with a relation women : men 3:1 being the fifth decade of life the most frequent starting age [5]. In Mexico the prevalence reported is 1.6%, although a big variation occurs in the epidemiology of the country in accordance to the studied region, in the Peláez Ballestas research in 2011 about the epidemiology in rheumatic diseases in Mexico, he found that the geographical variations suggest a role for the geographical factors in the prevalence of rheumatic diseases.

1.1.2 RA etiology.

The RA is a multifactorial disease, whereby, its exact etiology remains undefined, however, is considered that the development of the disease is related with the genetic predisposition and the exposure to a triggers from environmental factors [9, 10]. The genetic risk factor more important for the development of RA is the sequence known as shared epitope (SE), present in the different HLA-DRB1 alleles, that may be encountered up to a 78% of the patients with RA [11]. This genetic variation subserve the antigenic presentation to the T lymphocytes of the citrullinated peptides [12]. Moreover, into the triggering environmental factors, smoking is considered the most important risk factor for RA development. Padyukoy [13] in his study in 2004 note an interaction between gens and

environment between smoking and shared epitope gens HLA-DR providing a higher risk of seropositive rheumatoid arthritis. Later, Lundström [14] in his study in 2009 trace a strong interaction between the different alleles DRB1 and smoking, conferring a higher risk in the development of RA with positive ACPA. Additionally, it has been proposed that the tobacco smoke inhalation is related with increase in the expression of the peptidyl-arginine-deaminase enzyme (PAD) who generate increment at pulmonary level of citrullinated peptides [15]. Moreover, the hormonal deregulation in women's is associated to a higher risk of developing RA; Being greater its prevalence in the post-partum period and menopause.

1.1.3 RA Physiopathology.

Despite multiple efforts to unmask, the mechanisms of initiation and propagation of the RA, it haven't been possible to define them completely. Nevertheless, the autoimmunity and the chronic inflammatory process have an important role in the RA pathogeny. Being found in those mechanisms the T helper lymphocytes (Th) participation, as well as B lymphocytes (BL) with the antibodies generation [17]. The Th participation is through the proinflammatory cytokines production as the tumor necrosis factor alfa (TNF-α) and the interleukin 6 (IL-6), which have been found in a greater amounts, being TNF-α as the central cytokine in the pathogeny in the joint damage mechanism and the IL-6 as the principal endothelium and osteoclast activator. [17]. On the other hand, the autoantibodies generation made by BL, being the most described the RF and the ACPA [18] are antibodies that are directed to proteins that have suffered a post translational modification known as citrullation, in which the peptidyl-arginine conversion to peptidyl-citrulline is catalyzed for the enzymes family PAD [19]. The ACPA description and its presence in the patients serum have been a useful test in the RA diagnosis with a high specificity for the diagnosis of 90% [20] and a severity disease predictor [21, 22]. By the mentioned, is known that the inflammatory synovial process is complex with multiple immunologic mechanisms participation of simultaneous activity [23] generating joint damage [2] with clinical manifestation as pain, inflammation and peripheral joint stiffness [24] mainly in the proximal interphalangeal, metacarpophalangeal and carpal joints; followed by the metatarsophalangeal, ankles, shoulders, elbows [25] and extra-articular manifestations in

the 15% of the patients, such as vasculitis, neuropathy, pericarditis, pleuritis and scleritis [24].

1.1.4 Morbidity and Mortality in RA.

Frequently RA compromises other organs different to the joints and is considered as an independent cardiovascular risk factor [26] associated to a greater lethality than the general population [27] Previous studies have shown that RA reduces life expectancy in standardized mortality rates between 1.14 and 2.24 [28] In 2003, Salomón [27] & Cols described the follow-up of 114,342 healthy women with an average age between 30-55 years old, throughout 19 years (1977-1996) among them 527 had RA. In this study it was found that the patients who suffered RA had higher mortality than the healthy subjects with a mortality relation of 2:1. Crowson [29] In 2005 compared the proportion of risk for the development of heart failure (HF) and ischemic heart disease (IHD) attributable to traditional's cardiovascular risk factors between patients with and without RA. It was observed that the excess risk of HF and IHD in patients with RA is not explain with the increase of the frequency or the effect of cardiovascular risk factors being these factors adscribable in 54% in subjects with RA in comparation 77% in subjects without RA. Additionally, Kremers [30] in his study in 2008 estimated the absolute risk to 10 years of cardiovascular (CV) events in newly diagnosed RA and the potential contribution of CV risk in the evaluation of the absolute risk, in the found 16.8% of risk in CV diseases in subjects with RA without newly diagnosed CV risk factors and 60.4% of increment in presence to risk factors like smoking, hypertension, dyslipidemia, diabetes and obesity.

In posterior research, Crilly and Cols [31] in 2009, describe a dose-response relation between the accumulated inflammatory load and accumulated arterial stiffness in RA independent of the established risk factors. This study report 114 patients with RA (93 woman's and 23 men's) with average age of 53.7 years old (40-65) and a sickness duration of 9.6 (4.4-16.9) it was found a higher CV risk in his cardiology evaluation by means of augmentation index measurement, being this an 31.5 average value; what suggested the CV affectation of RA patients is associated with the increased arterial stiffness, similar results reported by Klocke in 2003 [32] and subsequently by Park in 2012 [33].

1.1.5 Arterial stiffness in rheumatoid arthritis.

The arterial stiffness is defined by Kozakova M (2015) like: “The reduced capacity of one artery to expand or contract in response to pressure changes, result of the vascular remodeling that produce changes in the collagen and elastin balance in the blood vessels walls” who is generated by the biological aging, arteriosclerosis, endothelial disfunction, chronic inflammation, accumulated damage of high mechanic tension, dyslipidemia, smoking, obesity, diabetes mellitus and vascular activation.

In RA, the arterial stiffness is mainly generated by related factors with the accelerated disease for the gender and the disease activity [34], the evolution time of the disease and C-reactive protein circulating levels [35], the auto-antibodies presence [36] and circulating cytokines, mainly TNF-α [37, 38] and IL6 [35]. These factors contribute to loose of endothelial function [39] vasodilatation capacity inhibition and arterial elasticity lost which affect the renin-angiotensin-aldosterone system (RAA) and vasodilatory molecules like bradykinin [40] being this the last one reported for first time by Erspamer group in 1960 as the final product of the kinin-kallikrein system in the blood system in mammals [41].

1. 2 Pulse wave velocity.

Previous studies have shown that arterial elasticity can be measured by central pulse wave velocity (cfPWV); which, relates the CV risk by the arterial distensibility evaluated [42]. Actually, the cfPWV is considered the gold standard for the arterial stiffness evaluation [43] and with the central augmentation index elevated (CAI) corroborate the elasticity reduction caused by structural changes in leading artery walls [35, 44] considering the increase of 1m/s up the cfPWV normal value according to age, the equivalent to 12% increase of CV risk, this was observed in the Ali R Khoshdel research in 2007.

cfPWV distribution (m/s) according age

in healthy subjects (1455)	
Age	Mean
<30	6.2
30-39	6.5
40-49	7.2
50-59	8.3
60-69	10.3
>70	10.9

Box 1. cfPWV standard values according age. European Society of Cardiology. European Heart Journal. 2010.

cfPWV is determine by two intervals, first the colocation of the tonometer in common carotid artery with a simultaneous electrocardiogram (EKG), and repeat the procedure in femoral artery. The delay in time belongs to the delay of the increase of the distal pulse wave of R belonging to QRS complex of the EKG of the second time and the apparition retard of the proximal pulse wave to the R belonging to the QRS complex of the first time. The cfPWV is obtained by many measurement instruments, one of them is the PulsePen, measurement instrument by non-invasive tonometry with the device (DiaTecne s.r.l., Milan, Italia) with a previous validated technique and showed efficacy to gauge cfPWV. cfPWV is calculated by: cfPWV = distance between two arterial segments / delay in time and its units of measurement are meters / seconds (m/s) [45].

Besides of the PWV measurement a new device called pOpmetre is proposed, which measures the arterial stiffness using infrared photodiodes in the hand finger and toe. The PWV measurement by pOpmetre is in function of the patient stature and the second derivation is the time difference between the pulse in each finger. On the other hand, is possible evaluate arterial stiffness and arteriosclerosis with the cardio-ankle vascular index (CAVI). CAVI is an oscillometric measurement tool, an arteriosclerotic exploration method for calculate the normal vascular function independent of the blood pressure, measure the blood pressure in the four extremities.

$$CAVI = \left[InPs/Pd \right] \cdot \frac{2\rho}{\Delta P} \cdot PWV^2 \quad \cdots (3)$$

Ps : Presión sistólica Pd : Presión diastólica

PWV : Velocidad de la onda de pulso entre el corazón y el tobillo

ρ : Densidad de la sangre ΔP : Presión del pulso

$$\beta = \left[InPs/Pd \right] \cdot \left[D/\Delta D \right] \quad \cdots (1)$$

Ps : Presión sistólica D : Diámetro

Pd : Presión diastólica ΔD : Cambio de diámetro

CAVI is calculated through B stiffness parameter obtained by equation Bramwell-Hill. The B parameter is used for diagnosed the carotid artery sclerosis grade, as of the variation of the diameter and the average blood pressure by ultrasonic echography.

1.3 Enalapril maleate.

Enalapril maleate is an angiotensin conversion enzyme inhibitor (ACE). Enalapril, is a pro-drug by enalapril maleate, designed for his oral administration.

1.3.1 Chemical structure of enalapril maleate.

Formula C20H28N2O5

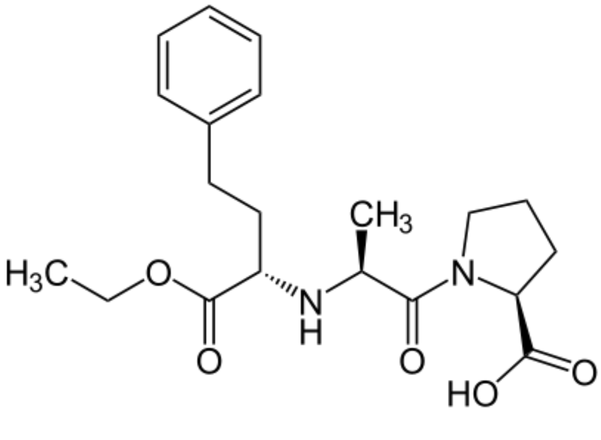

1.3.2 Metabolism and pharmacokinetic.

After oral administration of enalapril are observed maximum serum concentrations in 1 hour. Starting with the urine data, it follows that the enalapril is approximately 60% absorbed. Enalapril’s absorption is not affected by food presence in the digestive tract. Once absorbed, the enalapril is hydrolyzed to enalaprilat, the real ACE inhibitor. The maximum concentrations of enalaprilat are reached 4 hours after one oral dose of

enalapril. The quantity of drug do not increase with the dose, which indicate that the fixation point is saturable. The effective elimination half-life, determinate by kinetic data after multiples oral doses is 11 hours. The excretion of enalapril is highly renal. Approximately 94% of the administrated dose is recovered by the urine or stools as enalaprilat or enalapril.

1.3.3 Pharmacodynamic.

Enalapril administration in patients with mild to moderate hypertension causes the reduction of the arterial pressure in both supine and standing position, without being observed an orthostatic component. The symtomatic postural hypotension is infrequent, although it can occur in patients with volume depletion.

In most of the studied patients, after one enalapril dose, the anti-hypertensive effect start is observed one hour after the administration, occurring the highest blood pressure reduction at 6 hours. At the recommended dose the anti-hypertensive effect is maintained for at least 24 hours, although in some patients it is necessary to wait some weeks for the optimal reduction of blood pressure to be achived. The enalapril anti-hypertensive effect it is maintained during drug chronic administration and are not observed hypertensive rebound effects when abruptly discontinued.

1.3.4 Mechanism of action.

Oral enalapril, after being hydrolyzed to enalaprilat, inhibit the ACE both of men's and experimentation animals. ACE is an peptidyl-dipeptidase which catalyze the angiotensin I conversion to angiotensin II, a vasoconstrictor substance. Angiotensin II also stimulate the aldosterone secretion by the suprarenal cortex. The enalapril benefit effects in hypertension and HF are due to RAA system suppression [46].

RAA inhibition carries with a decrease of angiotensin II plasmatic levels, producing a vasopressore response decrease and aldosterone secretion. Besides the diminish of aldosterone secretion is not outheight, produce a little increase in potassium plasmatic

levels. Hypertensive patients treated with enalapril for 48 weeks, increased to 0.2 mEq/L. In patients treated with enalapril associated with thiazide diuretic, it was not observed changes in potassium levels.

Angiotensin II suppression produced, by a negative retro alimentation, an increase in renin levels. ACE is similar to kininase, enzyme uncharged to degrade bradykinin hence the suppression of its activity increased bradykinin levels, peptide with strongly vasodepressor effect. Is uncertain the role of this peptide in the enalapril therapeutic effect. Is secure that enalapril decrease the blood pressure acting in ACE system, is uncertain why this drug reduce the hypertension in renin low levels patients.

1.3.5 Enalapril indications.

Enalapril is indicated in all essential hypertension levels and renovascular hypertension. Can be used like initial treatment or concomitant with another antihypertensive drugs, specially diuretics. Enalapril is for oral administration only. For its absorption is not affected by food, enalapril can be administrated before, during and after foods. The usual daily dose goes from 10 to 40 mg in all indications. It can be administrated once or twice a day.

1.3.6 Adverse effects.

Enalapril is well tolerated. In clinics studies, the global incidence of side effects is not higher than placebo. For the most, side effects has been mild and transitory. The stop treatment was required in 6% of patients.

Secondary effects more commons was: instability sensation and headache. 2-3 per 100 patients was described: fatigue and asthenia. Other secondary effects with lower incidence of 2 out of 100 were: Orthostatic hypotension, syncope, nausea, diarrhea, muscle cramps and skin absorption. Its described dry and persistent cough with a frequency between 1 and 2 per 100, and could required the stop treatment. Hyper sensibility/angiogenic edema migth affect face, extremities, eyelids, tongue, glottis and/or larynx.

1.3.7 Findings in laboratory test.

It has been observed increase in urea and serum creatinine, reversible with the enalapril is discontinued.

2. Background.

It has been described in other diseases, the enalapril use for disminution of arterial stiffness. In 2002, J.L Tycho Vuurmans. And J Am Soc Neprhrol described the contribution in volume overload and angiotensin II in the PWV increased in hemodialyzed patients, in which was included 10 men's patients with a mild age 50 – 72 years in treatment with enalapril 5 mg once each 24 hours per 10 days and cfPWV evaluation (m/s) before and after hemodialysis (HD) with and without enalapril treatment, they found a significance diminution of cfPWV. A. Rehman found similar results in the diminution of cfPWV in the same year, using valsartan 80 mg per 3 days in 9 healthy men's patients of 24 years, before the measure.

In addition, Hideto Ishii (2008) got a cfPWV reduction in 11 patients with candesartan 8 mg per 90 days in diabetic type 2 patients and hypertension, and higher response with the use of candesartan in comparation with calcium blockers like amlodipine 6 mg and nifedipine 40 mg. Recently Ludovit Paulis (April 2016) reported a study with olmesartan per 6 weeks (10 mg/kg per day) in 65 male wistar mouse with hypertension through synthase nitric oxide inhibition with a significative PWV reduction.

3. Justification.

RA is an systemic chronic inflammatory disease considered as a CV risk factor independent which is associated to accumulative inflammatory charge with circulating levels elevated by inflammatory cytokines (CRP, TNF-α and IL6). This proinflammatory cytokines mediate the activation mechanism in endothelial cells with the increase of the ACE enzyme and the arterial stiffness generation.

Enalapril is a prodrug hydrolyzed in enalaprilat by hepatic esterase's, which is a higher inhibitor of ACE. The ACE inhibition reduce the systemic vascular resistance, the mean arterial pressure, diastolic and systolic pressure in hypertensive states, despite the mechanism, the ACE inhibitors have broad clinically utility for CV diseases.

Therefore, we consider that an association between enalapril use and arterial stiffness reduction is possible. However, actually the available information of enalapril use as prophylactic for IHD in RA patients is limited and with uncertain results. Therefore, the aim of this project is to explore the association between the use of enalapril and the decrease of arterial stiffness.

In order to submit this proposal, we decided to work in a multidisciplinary team to generate knowledge about the relationship between enalapril use and arterial stiffness diminution, to develop new therapeutic strategies for a better quality of life in RA patients.

4. Problem statement.

RA is an systemic chronic inflammatory disease considered as a CV risk factor per se, with high mortality rate compared to general population, due to a high prevalence of atherosclerosis and elevated arterial stiffness.

RA presents a premature arterial aging manifested as increased arterial stiffness due to the increase of expression of proinflammatory cytokines. Arterial stiffness is defined as the reduced capacity of artery to expand or contract in response to pressure changes, and ACE system is one of the important factors in arterial stiffness in other diseases.

These data suggest an association between vascular damage mechanisms in RA and ACE system. So we consider that blocking ACE system with enalapril will be an effective strategy to reduce arterial stiffness in RA patients.

4.1 Research question.

Which is the effect of enalapril administration in patients with rheumatoid arthritis in the arterial stiffness measurement?

5. Hypothesis.

Administration of enalapril decrease arterial stiffness in rheumatoid arthritis patients.

5.1 Alternative hypothesis.

Administration of enalapril increase arterial stiffness in rheumatoid arthritis patients.

5.2 Null hypothesis.

Administration of enalapril has no effect in arterial stiffness in rheumatoid arthritis patients.

6. Objectives.

6.1 General Objective.

Evaluate the enalapril effect in arterial stiffness in rheumatoid arthritis patients.

6.2 Specific objectives.

- Determinate the cfPWV by PulsePen and pOpmetre in RA patients at day zero and 90 days after enalapril treatment.
- Determinate CAVI in RA patients at day zero and 90 days after enalapril treatment.
- Determinate carotid index by carotid USG in RA patients at day zero and 90 days after enalapril treatment.
- Determinate antibodies anti-ACPA by ELISA and RF by immunoturbidimetry levels, before and after the intervention.
- Determinate proinflammatory cytokines serum levels TNF-α, IL6 and IL1 by ELISA in RA patients before and after the intervention.
- Evaluate the disease activity by “Disease activity score on 28 joints” (DAS-28) based in ESR and CRP, before and after the intervention.
- Determinate lipids serum levels in RA, cholesterol, triglycerides, LDL, VLDL, HDL and glucose, before and after the intervention.
- Report the tolerability of maleate of enalapril.

7. Materials and methods.

7.1 Study design.

Double blind clinical trial with randomization in parallel groups.

7.2 Study universe.

Volunteer women patients who attend to rheumatology outpatient clinic at the Hospital Civil " Dr. Juan I. Menchaca", with RA diagnosis according to ACR/EULAR 2010 criteria, without CV comorbidities, history or previous diagnostic of diabetes mellitus type 2, thyroid disease, hepatic or renal. All partipitants vollunteer accept to participate in this study and signed a written consent information.

7.3 Workplace.

This research project will be carried out in the Instituto de Investigación en Reumatología y del Sistema Músculo Esquelético del Centro Universitario de Ciencias de la Salud, Universidad de Guadalajara, Guadalajara University, Jalisco, Mexico.

7.4 Intervention and follow-up.

Two study groups will be conformed with 32 patients who met the inclusion and exclusion criteria and gave its consent to participate in this research project. Groups will be conformed by randomization by an aleatory number table, the volunteer will be assigned to one of the subsequent groups:

- Maleate enalapril group (5 mg each 12 hours)
- Placebo group (5 mg each 12 hours)

7.4.1 Selection or assignement of study subjects to study groups.

Will be included consecutively RA patients from rheumatology outpatient clinic at the Hospital Civil " Dr. Juan I. Menchaca" which fulfill inclusion and exclusion criteria and gave its written consent to participate in this research project.

8. Sample Height.

Sample Height was calculated with the continue response equation for clinical trials in parallel groups:

$$n = 2 \cdot \left[\frac{\sigma}{\Delta}\right]^2 \cdot c$$

Next values were considered for the equation substitution:

- σ represent primary outcome variable standard deviation, which corresponds to 1.3
- Δ shows the expected clinically relevant difference after maleate enalapril administration in arterial stiffness, which correspond to 1 m/s.
- c dependent constant on the confidence statistical level in 95% and statistical power 80%. Being equivalent to 7.9

Resulting in a sample Height of 26.7 to which are added 5.34 equivalent to 20% per loses resulting in a 32.04 total and considering a total of 32 subjects assumed by equal for both groups.

9. Selection criteria.

9.1 Inclusion criteria.

- Patients classified with RA according to the ACR / EULAR 2010
- Age (over 18 years old)
- Women
- Patients attending at the rheumatology outpatient clinic at the Hospital Civil " Dr. Juan I. Menchaca"
- Agreed to participate in the study and sign the informed written consent.
- Maintain rheumatological treatment with disease modifying drugs (DMARDS)

9.2 Non-inclusion criteria.

- Patients with previous diagnosis of diabetes mellitus, systemic hypertension, thyroid, kidney or liver disease
- History of acute myocardial infarction, cardiac arrhythmias, cerebral vascular event or heart failure
- Smoking patients active in the last 6 months
- Patients with a desire for close conception, pregnant or lactating.
- Patients with blood pressure <90/60 mmHg
- Patients who do not accept to participate in the study
- Patients with a BMI greater than ≥40 Kg / m2
- Non-palpable carotid and femoral pulses
- Unstable psychological state

9.3 Elimination criteria.

- Patients who withdraw their consent for the study

- Patients suffering from serious adverse effects to enalapril that results in the withdrawal from the study
- Subjects who leave the study due to medical or health reasons
- Patients with an attachment treatment less than 80%
- Patients who present extra-articular manifestations during the study and / or require modification of treatment with DMARS

10. Variables.

10.1 Independents.

- Administration of enalapril maleate.
- Placebo administration.

10.2 Dependents.

- Carotid-femoral pulse wave velocity by PulsePen
- Difference in carotid-femoral pulse wave velocity by PulsePen
- Progression of carotid-femoral pulse wave velocity by PulsePen
- Pulse wave velocity by pOpmetre
- Difference in pulse wave velocity by pOpmetre
- Progression of pulse wave velocity by pOpmetre
- Heart-ankle vascular index by CAVI
- Difference of the heart-ankle vascular index by CAVI
- Progression of heart-ankle vascular index by CAVI

10.3 Intervention.

- Nutrition (Diet, Weights, Height, BMI)
- Age
- Disease activity (DAS-28, ESR, CRP, RF, Anti-CCP, TNF-α)

10.4 Variables definition.

- **Age:** Time in years elapsed from the birth of an individual
- **Gender:** Biological sex one is born female or male

- **DAS-28: Disease Activity Score.** Disease activity measurement in which is considered the number of painful, swollen joints, analogous visual scale on the patient score, erythrocyte sedimentation rate and C-reactive protein.
- **HAQ: HEALTH ASSESSMENT QUESTIONNAIRE.** Instrument to assess disability, predict functional deterioration, work disability, premature mortality.
- **Disease evolution time:** Time elapsed from the disease diagnosis to the data of recruitment.
- **ESR:** Laboratory diagnostic test consists of measuring the speed with which red blood cells settle, it is an acute phase reactant, it is a nonspecific marker, whose elevation may involve inflammatory, infectious or neoplastic processes.
- **C-reactive protein:** It is a circulating plasma protein, which increases its levels in response to inflammation (acute phase protein).
- **Rheumatoid factor:** An autoantibody of the IgM type produced against the Fc portion of immunoglobulin G (IgG). Titers are elevated in certain rheumatic diseases and in some chronic infections.
- **Anti-CCP:** Antibodies against cyclic citrullinated peptides obtained from citrullinated proteins, highly specific for the diagnosis of RA. It will be considered as positive if in the ELISA test the absorbance is greater than 5 UI/mL.
- **Weight:** The weight is a gravitatoria force measure that acts on an object. The weight is equivalent to the force exerted by a body on a point of support, caused by the action of the local gravitational field on the mass of the body.
- **Height:** Is measure of vertical distance of a subject expressed in centimeters
- **Body mass index (BMI):** A measure that relates body weight to height, calculated with the formula, BMI = weight/height squared.
- **Central pulse wave velocity (cfPWV).** It is a pulse wave speed measurement that generates the left ventricle and spreads to the cardiovascular system, it is determined at intervals of two, first the placement of the tonometer in the common carotid artery and then the procedure in the femoral artery, it is calculated cfPWV = distance between two arterial segments/time delay and their units of measurement are meters/second (m/s)
- **Pulse Wave Velocity (PWV / pOpmetre).** It is a pulse wave speed measurement that generates the left ventricle and propagates to the cardiovascular system, it is determined using infrared photodiodes in the finger and the toe. The measurement

of PWV by pOpmétre is a function of the height of the patient and the second derivative of the time difference between the pulse of each finger.

- **Vascular Heart-Ankle Index.** It is an oscillometric measurement tool, an atherosclerotic exploration method to calculate normal vascular function independent of blood pressure, measures blood pressure in all four extremities. It is calculated by the stiffness parameter B obtained from the Bramwell-Hill equation.

10.5 Operationalization of variables.

Variables		Measurement Unit	Scale	Descriptive	Inferential
**Independent**	Enalapril Maleate	-	Qualitative nominal	Proportion	X^2
	Placebo	-	Qualitative nominal	Proportion	X^2
**Dependent**	Central pulse wave velocity (PulsePen)	m/s	Quantitative	Mean/SD	Student's t
	cfPWV Difference (PulsePen)	m/s	Quantitative	Mean/SD	Student's t
	cfPWV Progression (PulsePen)	m/s	Quantitative	Mean/SD	Student's t
	PWV (pOpmetre)	m/s	Quantitative	Mean/SD	Student's t
	PWV Difference (pOpmetre)	m/s	Quantitative	Mean/SD	Student's t
	PWV Progression (pOpmetre)	m/s	Quantitative	Mean/SD	Student's t
	Vascular index (CAVI)	-	Quantitative	Mean/SD	Student's t
	Vascular index difference (CAVI)	-	Quantitative	Mean/SD	Student's t
	Vascular index Progression (CAVI)	-	Quantitative	Mean/SD	Student's t
**Intervener**	Age	years	Quantitative	Mean/SD	Wilcoxon and U de Mann Whithey

Disease	FR	mg/dl	Quantitative	Mean/SD	Student's t
	Anti-CCP	UI/mL	Quantitative	Mean/SD	Student's t
	DAS-28	-	Quantitative	Mean/SD	Student's t
	HAQ	-	Quantitative	Mean/SD	Student's t
	ESR	mm/h	Quantitative	Mean/SD	Student's t
	CRP	mg/dl	Quantitative	Mean/SD	Student's t
Nutrición	Weight	kg	Quantitative	Mean/SD	Student's t
	Height	cm	Quantitative	Mean/SD	Student's t
	BMI	kg/m^2	Quantitative	Mean/SD	Student's t
	WC		Quantitative	Mean/SD	Student's t

11. Study group.

Two groups of 32 patients each will be formed:

1. Placebo.
2. Enalapril maleate.

Enalapril maleate group at a dose of 5 mg every 12 hrs orally, before each food and placebo group one dose every 12 hrs orally, before each food, both groups for 90 days.

11.1 Assignment of medication intervention and blinding.

The conformation of the groups will be carried out by randomization with codes obtained by random number generator to one of the two groups, according to the double blind code with random numbers. Neither the patient nor the researcher will know which pharmacological intervention corresponds to each group.

12. Clinical determinations.

• **Age:** Years completed

• **Gender:** M (Masculine) and F (Feminine) will be recorded.

• **Weight.** The weight (kg) will be taken using the TANITA BC418® Tokio, JPN system, with 0.01 kg of precision).

• **Height.** Vertical distance (cm) with the seca 214 stadiometer (GmbH & Co. KG Hamburg, Germany with 1 mm accuracy). For its measurement, the participant will be asked to take off their shoes, maintain the standing position with the heels together and around 5cm separation between their first toes. In an upright position, the outer commissure of one of the eyes was aligned with the ipsilateral external auditory canal orifice.

• **Body mass index.** The body mass index (BMI) will be calculated with the following formula BMI = WEIGHT (kg)/HEIGHT^2 (cm) = kg/m^2.

• **Waist circumference:** Measured with a Lufkin^{MR} tape should be performed at the level of the mid-axillary line, at the midpoint between the lower part of the last rib and the highest part of the hip. It is performed with the patient in standing position, and at the end of a normal expiration without clothes.

• **Peripheral blood pressure.** Peripheral arterial pressure measured in the office will be determined by the OMRON® HEM 907XL Tokyo Japan digital automatic oscillometric sphygmomanometer, with the patient sitting comfortably, with the feet well supported on the floor and after 5 min of rest, the bracelet will be selected according to the brachial circumference of 22-32 cm the medium bracelet will be used, and 32-42 cm will be used the large bracelet, it fits 3 cm above the elbow fold of the left arm to perfectly surround the brachial circumference: blood pressure (following international recommendations, O'Brien, Asmar et al., 2005)

• **Breathing frequency.** It will be measured by direct auscultation of the lung area and is defined as the number of expiratory and inspiratory cycles completed in a period of 60 seconds.

• **Heart rate** It will be measured by direct auscultation of the cardiac area and is defined as the number of heartbeats in a period of 60 seconds.

• **Temperature.** It will be measured by mercury thermometer, in the axillary region for 3 min, the measurement will be in °C.

• **DAS-28** The activity of the disease will be evaluated with the Disease activity index (DAS-28). First, the RA patient will mark on the analog visual scale (AVE) for general health in a line with a length from 0 to 100 mm, where 0 mm is equivalent to the best possible state of greeting and 100 to the worst; then the count of 28 joints will be made and the number of painful joints (NTJ) and swollen joints (NSJ) will be recorded in annex 8. The DAS-28 will be calculated with the formula: DAS-28 = 0.56 (√NTJ) +0.28 (√NSJ) +0.7 (lnESR) +0.14 (AVE).

• **Questionnaire of hereditary, pathological, non-pathological and gynecological-obstetric personal antecedents.** It will be carried out by means of an evaluation instrument which will be carried out through direct questioning.

• **Central Pulse Wave Velocity (cfPWV).** Will be measured the speed of the pulse wave generated by the left ventricle and its propagation to the cardiovascular system, it is determined at intervals of two, first placing the tonometer in the common carotid artery and then the proceeding into the femoral artery, is calculated cfPWV = distance between two arterial segments / time delay and their units of measurement are meters/second (m/s)

• **Pulse Wave Velocity (PWV / pOpmetre).** Will be measured the PWV that generates the left ventricle and spreads to the cardiovascular system, is determined using infrared photodiodes in the finger and toe. The measurement of PWV by pOpmétre is in function of the Height of the patient and the second derivative of the time difference between the pulse of each finger.

• **Vascular Heart-Ankle Index.** Blood pressure is measured in all four extremities. It is calculated by the stiffness parameter B obtained from the Bramwell-Hill equation.

CLINICAL DETERMINATIONS.		
Determination	Instrument	Method
Age	Interrogation	Years Old
Weight	TANITA BC418® Tokio, JPN, with 0.01 kg of precision.	Isak (International Society for the Advancement of Cineanthropometry)
Height	Stadiometer Seca 214 (GmbH & Co. KG. Hamburg, Germany 1 mm accuracy).	
BMI	BMI formula = Weight (kg)/Height^2 (cm) = kg/m^2	WHO (World Health Organization)
WC	Fiberglass tape measure	
BP	Blood pressure cuff	AHA (American Association of the Heart)
BR	Stethoscope	Number of expiratory and inspiratory cycles completed (60s)
HR	Stethoscope	number of heartbeats (60 s)
DAS-28	Instrument. EVA (0-100mm) 28 joints D and T	equation:DAS-28= 0.56(√NAD)+0.28(√NAT)+0.7(InESR)+0.14(EVA).
HAQ	Disability evaluation instrument	>0.5= affectation
cfPWV	Pulse Pen (DiaTecne s.r.l., Milán, Italy)	cfPWV = distance between two arterial segments/time delay and their units of measurement are meters/second (m/s)
PWV	pOpmetre (Axelife sas 300)	PWV = the second derivative of the time difference between the pulse of each finger, (m/s)
Vascular Index	CAVI (VaSera 1000)	It is calculated by the stiffness parameter B obtained from the Bramwell-Hill equation.

13 Laboratory determinations.

13.1 Obtaining biological samples.

By venous puncture in the arm to obtain blood samples in vacutainer® system tubes (BD Diagnostic Systems Montenegro 1402, (C1427AND) Buenos Aires - Argentina), with anticoagulant EDTA-K3 1 mg/mL, CPT tube for ESR, and without anticoagulant, once coagulated at room temperature, it was centrifuged at 1509 RCF (Rotanta 460R, Andreas HettichGmbH & Co. KG.) for 10 min. Serum will be separated into aliquots to be stored at -80 °C until analysis.

- Erythrocyte sedimentation rate (ESR) mm/h, will be measured by the Westergren method. A Westergren pipette -in a position of 90° with respect to the surface- will be loaded with a sample of anticoagulated blood with previously homogenised citrate and at the moment of reaching the 0 mm mark, the count will start up to 60 minutes, at this moment erythrocyte sedimentation will be read and recorded in mm/h.
- High sensitivity C-reactive protein (hs-CRP, mg/L), by turbidimetry, antigen-antibody reaction against human C-reactive protein, with limit of detection of 0.15 mg/L, and rheumatoid factor with the Selectra E system, Randox laboratories.
- The ACPA positivity will be measured with a commercial anti-CCP2 antibody kit from Axis Shield.
- The positivity of rheumatoid factor will be measured with a commercial kit of antibodies.
- The glucose oxidase technique, expressed in mg/dl, will be used to determine serum glucose. Reactivo Merck México, S.A. The glucose oxidase technique oxidizes glucose and generates gluconic acid and H_2O_2.The liberated hydrogen peroxide reacts with a chromogen (phenol/4-aminoantipyrine) by the Trinder reaction, to give a quinone that absorbs between 492 and 550 nm. The intensity of color produced is directly proportional to the concentration of glucose.
- Total cholesterol (TC), HDL-C, LDL-C and triglycerides (TG) will be evaluated by the colorimetry technique, in which *Vitros* reagents are used, Ortho-Clinical Diagnostics, Inc. to Johnson-Johnson Company, Rochester, NY, USA. The

technique is as follows: the Vitros reagent, is a dry, multilayer analytical element, incorporated in a polyester support in which a drop of the patient's serum sample is deposited, which is evenly distributed through the diffusing layer to the underlying layers. Subsequently the reaction between the sample and the element to be determined is given to produce hydrogen peroxide. Finally, hydrogen peroxide oxidizes a dye in the presence of peroxidase to generate a color. The density of the dye formed is proportional to the concentration of the metabolite present in the sample and is measured by reflection spectrophotometry.

Laboratory Determinations

Determination	Instrument	Method
ESR	Westergreen pipette (60 min)	Westerngreen method
CRP	Selectra E system, Randox laboratories	turbidimetry
RF	Selectra E system, Randox laboratories	turbidimetry
Anti-CCP	commercial anti-CCP antibody kit from Axis Shield.	ELISA
Glucose	Merck Mexico Reagent	glucose oxidase technique
TC, HDL, LDL, TG	Reactivos Vitros, Ortho-Clinical Diagnostics, Inc. Johnson-Johnson Company, Rochester, NY, USA	colorimetry technique

14. Statistical analysis.

For the statistical analysis, two groups will be considered: 1) Treatment group with enalapril 5 mg/12 h; and 2) Control group with placebo /12 hrs. The distribution of the variables will be evaluated with the Kolmogorov-Smirnov test. According to its distribution, the continuous variables will be reported as mean ± standard deviation or median (interquartile range). The categorical variables will be reported as frequencies. Continuous variables were compared using the Student's t test for dependent variables or the Wilcoxon test depending on their distribution. Categorical variables by χ^2 test or Fisher 's exact test as appropriate. A value of P <0.05 will be considered as statistically significant. An ANCOVA analysis was carried out with backward mode and a P = 0.05 for entrance and P = 0.10 for elimination. All data were analyzed using SPSS 24.0 software (SPSS Inc. Chicago, IL) and GraphPad Prism version 6.00 for Windows (GraphPad Software, La Jolla, CA), considering a two-tailed level of P <0.05 to be statistically significant for analysis.

15. Study description.

15.1 Field phase development.

Visit 0, Scrutiny (Day -1).

All prospective candidates will be cited at 08:00 a.m. with a 12-hour fast. In this first visit, information regarding the study will be provided; the objectives, characteristics, procedures, as well as their potential benefits and risks. If the individual accepts and the person in charge of the investigation decides that the patient meets the criteria for participation in the study, will be signed the informed written consent letter. The clinical history and anthropometric measures will be taken (weight, height, BMI, percentage of body fat, waist circumference) vital signs (peripheral arterial pressure, heart rate and respiratory rate), evaluation of the state of the disease (DAS-28, HAQ). The blood sample will be taken (10 ml of blood) to measure fasting glucose, lipid profile, CRP, ESR, antibodies (RF and anti-CCP).

Visit 1. Baseline: Review of criteria.

Laboratory results are valued and it is corroborated that the individual meets all the inclusion criteria and if this is the case, the randomization of their treatment will proceed, the patient will be able to enter the group studied with enalapril or placebo (the enalapril treatment will consist of 5 mg every 12 hrs before each food, like the placebo group). The pulse wave velocity and vascular index will be measured, and a diary will be given to the patient describing the daily posology, and the medication corresponding to the first month of the intervention will be delivered, and finally, general nutrition recommendations will be given since during the study it is not indicated to initiate some type of diet to lose weight or an increase in the physical activity. The telephone number of the managers will also be included for any questions the patient may have regarding the project. The control patient will be scheduled 30 days after starting the treatment.

Visit 2 and 3. Follow-up (Days 30, 60 ± 3 respectively).

During the first and second month of the intervention will be cited to monitor attachment and tolerability to treatment using questionnaires, anthropometric measurements, vital signs will be taken and the medication corresponding to the current month will be provided.

Visit 4. End of the intervention period (Day 90± 3).

After 90 days at the beginning of the intervention, the individual will be re-quoted and clinical and laboratory measurements will be taken in the same way as at the baseline visit, with a 12-hour fast. Attachment and tolerability to treatment will be evaluated.

Visit 5: Evaluation and delivery of results (Day 120± 3).

After 4 weeks the patient will be summoned to deliver results that include: scrutiny, baseline and end of the intervention and the evolution of each of them will be explained. Finally, a clinical summary will be delivered and it will have medical-nutritional recommendations, as well as constant medical surveillance if necessary.

15.2 Therapeutic adherence.

Adherence to treatment will be evaluated by indirect methods, questionnaire answered by the same patient. The adherence rate to the treatment is reported as a percentage individually. Although there is no consensus that defines the cut-off point to determine an adequate adherence, it will be taken into account as acceptable therapeutic adherence to more than 80% of the prescribed doses of the investigational drug.

15.3 Security criteria.

All patients will be monitored for the possible presence of adverse events of any kind throughout the study. The presence of adverse events will be monitored. From the beginning of the study, patients will have the responsible investigators emergency telephone numbers. Throughout the follow-up period there is the possibility of carrying out laboratory determinations in case of suspected adverse events or toxic effects of the medication. It will be considered as loss and abandonment of follow-up when the patient misplaces the drug that was provided and the responsible person is not informed in time or has less than 80% adherence.

15.4 Data collection.

It will be carried out by clinical history, a database will subsequently be made to monitor the patient progress, the proper management and discretion of the patient's data will be monitored at all times, and an electronic database will be integrated.

15.5 Dissemination of the final results of the investigation.

The results will be delivered confidentially to each of the participants, as well as special measures that merit self-care or monitoring according to the results. The study results will be published in a scientific journal indexed, presentation at events like congresses and conferences related to research.

16. Ethical aspects.

The study fulfill with international standards for research in humans, according to the General Law of Health on the ethical principles of research in humans Article 17 section 3 is considered as a research with a risk greater than the minimum and in compliance with the ethical principles for pharmacological research in human beings detailed in the last revision of the Declaration of Helsinki and the International Conference on Harmonization.

The nature, purposes and potential risks of the study will be explained to each participant. All patients will give their consent under information with the signature of witnesses and their relationship with them. Acording to the guidelines of good clinical practice, all study participants will be identified only by initials and number in the database. The records and laboratory results will be available only to the principal investigators and with the restrictions of law for the participant. Voluntary patients have the right to withdraw when they wish, with the previous notice to their medical researcher without affecting their medical-patient relationship or affecting their concomitant treatment. It will also proceed on the basis of NOM-012-SSA-2012 that establishes the criteria for the execution of research projects for health. NOM 007-SSA3-2011 for the clinical laboratories organization and operation. In matters of biosafety, proceed are according to NOM-087-ECOL-SSA1-2002. That establishes the requirements for the separation, packaging, storage, collection, transportation, treatment and final disposal of hazardous biological-infectious waste generated in establishments that provide medical care services. The hazardous biological-infectious residues (HBIW) to be used will be blood, defined as the blood tissue with all its elements, the non-anatomical residues, sharp objects, the handling of HBIW will comply with the corresponding provisions such as identification of the waste, packaging, temporary storage, collection and transportation specified in the same NOM-087.

The handling of chemical reagents will be in accordance with the NOM-018-STPS-2000 system for the identification and communication of hazards and risks due to dangerous chemical substances in the workplace. The risk degree of the chemical reagents use are consistent with risk equal to 0 or 1, specifications such as identification, signage, personal protective equipment, and proper handling stipulated in NOM-018 will be met.

17. Financing.

The present study does not currently have its own sponsorship, its funding comes from the Postgraduate Program of Incorporation and Permanence in the National Postgraduate Program of Quality (PROINPEP) fron the National Council of Science and Technology (CONACyT), in the same way, funding is obtained parallel to other studies with a fund of $ 166,190 MNX,. The total financial budget required for the preparation of this project is $ 90,089.8 MNX

18. Human resources.

18.1 Work team.

Research group on arterial stiffness in rheumatoid arthritis

Centro Universitario de Ciencias de la Salud, C. U. C. S. Universidad de Guadalajara.

For the development of the project, we have the necessary human resources to carry out the laboratory analysis and evaluation of patients in the Instituto de Investigación en Reumatología y del Sistema Musculo-Esquelético (IIRSME), as well as in the Instituto de Terapéutica Experimental y Clínica (INTEC). The main function of each of them is described briefly:

MD, PhD. Mónica Vázquez del Mercado Espinosa. - Principal Investigator. Director of the Instituto de Investigación en Reumatología y Sistema Músculo Esquelético (IIRSME). Rheumatologist and Doctor in Molecular Biology.

PhD Rosa Elena Navarro Hernández. - Investigador. Researcher. In charge of laboratory of the Instituto de Investigación en Reumatología y Sistema Músculo Esquelético (IIRSME). Chemist biologist and doctor in Biomedical Sciences.

MD, PhD. José de Jesús Eduardo Gómez Bañuelos. - Researcher. Research of the Investigación en Reumatología y Sistema Músculo Esquelético (IIRSME). Rheumatologist

and doctor in biomedical sciences with orientation in immunology, in charge of the clinical-rheumatological evaluation of patients.

MD. Felipe de Jesús Pérez Vázquez. – Researcher. PhD student in Pharmacology from the Instituto de Investigación en Reumatología y Sistema Músculo Esquelético (IIRSME), Charge of patient appointments, clinical evaluation, monitoring of patients during the study, capture of data and delivery of results.

Gustavo Ignacio Díaz Rubio. - PhD student of Molecular Biology in Medicine program from the Instituto de Investigación en Reumatología y Sistema Músculo Esquelético (IIRSME). Drug-biologist chemist in charge of taking samples, processing them and evaluating the results.

PhD. Efraín Chavarría Ávila. - Researcher. Research from the Investigación en Reumatología y Sistema Músculo Esquelético (IIRSME). Chemist, Biologist and doctor in Biomedical Sciences with orientation in immunology, diploma in biostatistics, in charge of the statistical evaluation of the data, statistical analysis and preparation of tables and graphs.

PhD. Ernesto German Cardona Muñoz. - Researcher. Director from the Instituto de Terapéutica Experimental y Clínica (INTEC). Cardiologist and Doctor in Pharmacology.

PhD. David Cardona Müller. - Researcher. Instituto de Terapéutica Experimental y Clínica (INTEC). Cardiologist, in charge of cardiological assessment of patients, as well as the performance of carotid ultrasound.

PhD. Fernando Grover Páez. - Researcher. Instituto de Terapéutica Experimental y Clínica (INTEC). Internist Doctor, Doctor in Pharmacology, responsible for the measurement of the heart-ankle index (CAVI) and evaluation of patients for suspected adverse effects to the medicine.

PhD. Carlos Gerardo Ramos Becerrra. - Researcher. Instituto de Terapéutica Experimental y Clínica (INTEC). Doctor in Hypertension, responsible for the measurement of pulse wave velocity by means of pOpmetre and pulsepen.

Lic. en Nutrición Lesli Yazmín Lozano Padilla. - Nutritionist from the Instituto de Investigación en Reumatología y Sistema Músculo Esquelético (IIRSME). Responsible for the evaluation of nutrition and anthropometric measurements of patients.

Mrs. Cynthia Alejandra Gómez Ríos. – Assistent of the Instituto de Investigación en Reumatología y Sistema Músculo Esquelético (IIRSME). Responsible for the administration of the institute and reception of patients.

19. Material resources.

For the development of the project, we have the necessary material resources to carry out the laboratory analysis and evaluation of the patients in the Instituto de Investigación en Reumatología y del Sistema Musculo-Esquelético. In brief, we have centrifuges for the separation of the samples, ultra-freezers for the conservation of them. Spectrophotometer for reading the ELISA plates and equipment for the analysis of the images obtained by chemiluminescence. The pulsepen, pOpmetre and CAVI are available for cardiovascular analysis at the Instituto de Terapéutica Experimental y Clínica (INTEC).

20. Conflicts of interest.

The present study does not present conflicts of interest of any kind, such as agreements with the pharmaceutical industry or those of the principal investigator or the personnel involved in the study.

21. Bibliographic references.

1. Gossec, L., M. Dougados, and W. Dixon, Patient-reported outcomes as end points in clinical trials in rheumatoid arthritis. RMD Open, 2015. 1(1): p. e000019.
2. Burmester, G.R., E. Feist, and T. Dorner, Emerging cell and cytokine targets in rheumatoid arthritis. Nat Rev Rheumatol, 2014. 10(2): p. 77-88.
3. Gibofsky, A., Overview of epidemiology, pathophysiology, and diagnosis of rheumatoid arthritis. Am J Manag Care, 2012. 18(13 Suppl): p. S295-302.
4. Silman, A.J. and J.E. Pearson, Epidemiology and genetics of rheumatoid arthritis. Arthritis Res, 2002. 4 Suppl 3: p. S265-72.
5. Tobon, G.J., P. Youinou, and A. Saraux, The environment, geo-epidemiology, and autoimmune disease: Rheumatoid arthritis. Autoimmun Rev, 2010. 9(5): p. A288-92.
6. Cardiel, M.H. and J. Rojas-Serrano, Community based study to estimate prevalence, burden of illness and help seeking behavior in rheumatic diseases in Mexico City. A COPCORD study. Clin Exp Rheumatol, 2002. 20(5): p. 617-24.
7. Bennett, K., et al., Community screening for rheumatic disorder: cross cultural adaptation and screening characteristics of the COPCORD Core Questionnaire in Brazil, Chile, and Mexico. The PANLAR-COPCORD Working Group. Pan American League of Associations for Rheumatology. Community Oriented Programme for the Control of Rheumatic Disease. J Rheumatol, 1997. 24(1): p. 160-8.
8. Pelaez-Ballestas, I., et al., Epidemiology of the rheumatic diseases in Mexico. A study of 5 regions based on the COPCORD methodology. J Rheumatol Suppl, 2011. 86: p. 3-8.
9. Karlson, E.W. and K. Deane, Environmental and gene-environment interactions and risk of rheumatoid arthritis. Rheum Dis Clin North Am, 2012. 38(2): p. 405-26.
10. Too, C.L., et al., Smoking interacts with HLA-DRB1 shared epitope in the development of anti-citrullinated protein antibody-positive rheumatoid arthritis: results from the Malaysian Epidemiological Investigation of Rheumatoid Arthritis (MyEIRA). Arthritis Res Ther, 2012. 14(2): p. R89.
11. Bax, M., et al., Genetics of rheumatoid arthritis: what have we learned? Immunogenetics, 2011. 63(8): p. 459-66.

12. Feitsma, A.L., et al., Identification of citrullinated vimentin peptides as T cell epitopes in HLA-DR4-positive patients with rheumatoid arthritis. Arthritis Rheum, 2010. 62(1): p. 117-25.
13. Padyukov, L., et al., A gene-environment interaction between smoking and shared epitope genes in HLA-DR provides a high risk of seropositive rheumatoid arthritis. Arthritis Rheum, 2004. 50(10): p. 3085-92.
14. Lundstrom, E., et al., Gene-environment interaction between the DRB1 shared epitope and smoking in the risk of anti-citrullinated protein antibody-positive rheumatoid arthritis: all alleles are important. Arthritis Rheum, 2009. 60(6): p. 1597-603.
15. Makrygiannakis, D., et al., Smoking increases peptidylarginine deiminase 2 enzyme expression in human lungs and increases citrullination in BAL cells. Ann Rheum Dis, 2008. 67(10): p. 1488-92.
16. Pikwer, M., et al., Early menopause and severity of rheumatoid arthritis in women older than 45 years. Arthritis Res Ther, 2012. 14(4): p. R190.
17. Brzustewicz, E. and E. Bryl, The role of cytokines in the pathogenesis of rheumatoid arthritis--Practical and potential application of cytokines as biomarkers and targets of personalized therapy. Cytokine, 2015. 76(2): p. 527-536.
18. van Boekel, M.A., et al., Autoantibody systems in rheumatoid arthritis: specificity, sensitivity and diagnostic value. Arthritis Res, 2002. 4(2): p. 87-93.
19. Hensen, S.M. and G.J. Pruijn, Methods for the detection of peptidylarginine deiminase (PAD) activity and protein citrullination. Mol Cell Proteomics, 2014. 13(2): p. 388-96.
20. Mouterde, G., et al., Association of anticyclic citrullinated peptide antibodies and/or rheumatoid factor status and clinical presentation in early arthritis: results from the ESPOIR cohort. J Rheumatol, 2014. 41(8): p. 1614-22.
21. Gerli, R., et al., Association of anti-cyclic citrullinated peptide antibodies with subclinical atherosclerosis in patients with rheumatoid arthritis. Ann Rheum Dis, 2008. 67(5): p. 724-5.
22. Luban, S. and Z.G. Li, Citrullinated peptide and its relevance to rheumatoid arthritis: an update. Int J Rheum Dis, 2010. 13(4): p. 284-7.
23. McInnes, I.B. and G. Schett, Cytokines in the pathogenesis of rheumatoid arthritis. Nat Rev Immunol, 2007. 7(6): p. 429-42.

24. van der Helm-van Mil, A.H., et al., A prediction rule for disease outcome in patients with recent-onset undifferentiated arthritis: how to guide individual treatment decisions. Arthritis Rheum, 2007. 56(2): p. 433-40.
25. Huizinga, T.W. and A. van der Helm-van Mil, A quantitative approach to early rheumatoid arthritis. Bull NYU Hosp Jt Dis, 2011. 69(2): p. 116-21.
26. Gkaliagkousi, E., et al., Cardiovascular risk in rheumatoid arthritis: pathogenesis, diagnosis, and management. J Clin Rheumatol, 2012. 18(8): p. 422-30.
27. Solomon, D.H., et al., Cardiovascular morbidity and mortality in women diagnosed with rheumatoid arthritis. Circulation, 2003. 107(9): p. 1303-7.
28. Kramer, H.R. and J.T. Giles, Cardiovascular disease risk in rheumatoid arthritis: progress, debate, and opportunity. Arthritis Care Res (Hoboken), 2011. 63(4): p. 484-99.
29. Crowson, C.S., et al., How much of the increased incidence of heart failure in rheumatoid arthritis is attributable to traditional cardiovascular risk factors and ischemic heart disease? Arthritis Rheum, 2005. 52(10): p. 3039-44.
30. Kremers, H.M., et al., High ten-year risk of cardiovascular disease in newly diagnosed rheumatoid arthritis patients: a population-based cohort study. Arthritis Rheum, 2008. 58(8): p. 2268-74.
31. Crilly, M.A., et al., Arterial stiffness and cumulative inflammatory burden in rheumatoid arthritis: a dose-response relationship independent of established cardiovascular risk factors. Rheumatology (Oxford), 2009. 48(12): p. 1606-12.
32. Klocke, R., et al., Arterial stiffness and central blood pressure, as determined by pulse wave analysis, in rheumatoid arthritis. Ann Rheum Dis, 2003. 62(5): p. 414-8.
33. Park, S. and E.G. Lakatta, Role of inflammation in the pathogenesis of arterial stiffness. Yonsei Med J, 2012. 53(2): p. 258-61.
34. Holmes, M.V., et al., Paradoxical association of C-reactive protein with endothelial function in rheumatoid arthritis. PLoS One, 2010. 5(4): p. e10242.
35. Roman, M.J., et al., Arterial stiffness in chronic inflammatory diseases. Hypertension, 2005. 46(1): p. 194-9.
36. Vazquez-Del Mercado, M., et al., Serum levels of anticyclic citrullinated peptide antibodies, interleukin-6, tumor necrosis factor-alpha, and C-reactive protein are associated with increased carotid intima-media thickness: a cross-sectional analysis of a cohort of rheumatoid arthritis patients without cardiovascular risk factors. Biomed Res Int, 2015. 2015: p. 342649.

37. Vassilopoulos, D., et al., Adalimumab decreases aortic stiffness independently of its effect in disease activity in patients with rheumatoid arthritis. Clin Rheumatol, 2015. 34(2): p. 359-64.
38. Angel, K., et al., Tumor necrosis factor-alpha antagonists improve aortic stiffness in patients with inflammatory arthropathies: a controlled study. Hypertension, 2010. 55(2): p. 333-8.
39. Wallberg-Jonsson, S., et al., Increased arterial stiffness and indication of endothelial dysfunction in long-standing rheumatoid arthritis. Scand J Rheumatol, 2008. 37(1): p. 1-5.
40. Wong, M., et al., Reduced arterial elasticity in rheumatoid arthritis and the relationship to vascular disease risk factors and inflammation. Arthritis Rheum, 2003. 48(1): p. 81-9.
41. Xi, X., et al., A review on bradykinin-related peptides isolated from amphibian skin secretion. Toxins (Basel), 2015. 7(3): p. 951-70.
42. Wilkinson, I.B., et al., Reproducibility of pulse wave velocity and augmentation index measured by pulse wave analysis. J Hypertens, 1998. 16(12 Pt 2): p. 2079-84.
43. Laurent, S., et al., Expert consensus document on arterial stiffness: methodological issues and clinical applications. Eur Heart J, 2006. 27(21): p. 2588-605.
44. Yildiz, M., Arterial distensibility in chronic inflammatory rheumatic disorders. Open Cardiovasc Med J, 2010. 4: p. 83-8.
45. Townsend, R.R., et al., Recommendations for Improving and Standardizing Vascular Research on Arterial Stiffness: A Scientific Statement From the American Heart Association. Hypertension, 2015. 66(3): p. 698-722.
46. Igic, R. and R. Skrbic, The renin-angiotensin system and its blockers. Srp Arh Celok Lek, 2014. 142(11-12): p. 756-63.

22. ATTACHED.

22.1 ATTACHED 1. CONSENT LETTER UNDER INFORMATION

“Effect of the use of enalapril on arterial stiffness in patients with rheumatoid arthritis”.

Do not sign this consent under information if you do not agree to participate in the study. The document may contain information or terms that you do not understand, please ask the medical officer in charge or, if appropriate, the doctor designated for your interview to solve all your doubts.

Rheumatoid arthritis is a disease that affects the joints generating pain and joint deformity. Rheumatoid arthritis in addition, damages blood vessels causing arterial stiffness and often compromising the heart.

Enalapril maleate, commonly known as enalapril, is a drug that has been shown to have beneficial effects in the treatment of arterial stiffness in other diseases. Enalapril is a drug that is eliminated by the kidney, and is generally well tolerated, adverse effects have been reported: tiredness, weakness, low blood pressure, fainting, nausea, headache, diarrhea, muscle cramps, allergy, cough dry, swelling of the eyelids and tongue; which have been presented in low frequency and 94% of the cases does not merit suspension of the medication.

Corn starch is a food product that is used in the research area under the name of "placebo" which is considered as a substance which has no beneficial or maleficent property in the body.

The study will be blinded, meaning neither the investigator in charge, nor you, will know who is taking enalapril or placebo, unless required or until the end of the study. Both will be provided free of charge, with sufficient quantity and with indications of how you should take it, at any time you can communicate doubts or inconveniences that the treatment causes.

The purpose of the study.

The objective of the study is to evaluate and monitor your levels of pulse wave velocity, vascular index and other laboratory studies after the administration of enalapril or placebo.

Description of the study.

64 patients participated in this study; 32 patients per group.- Enalapril group and placebo group . If you wish to participate, a file will be opened and the clinical history will be done, laboratory and clinical tests will be taken as weight, height, waist circumference and vital signs. This study lasts 3 months, with 6 visits which are written below:

-Visit 1 (Scrutiny): The candidate will be summoned at 08:00 a.m. with 12 hours of fasting. In this first visit, information regarding the study is provided; the objectives, characteristics, procedures, as well as their benefits and risks. If you accept and the person in charge of the study decides that you meet the selection criteria for participation in the study, you will proceed to sign this consent letter. The clinical history, rheumatological medical examination, measurements and vital signs (weight, height, BMI, percentage of body fat, waist circumference, blood pressure, heart rate and respiratory rate), blood sample taking (10 ml of blood / is equivalent to 2 tablespoons) to measure glucose, cholesterol, triglycerides, erythrocyte sedimentation rate, C-reactive protein, levels of tumor necrosis factor alpha (TNF a), interleukin-6 (IL-6) and antibodies against factor Rheumatoid (FR) and anti-citrullinated antibodies (anti-CCP). For its safety and hygiene, all the material used in this study is sterile and disposable and at the end of the planned analyzes, the rest of the sample will be destroyed. The approximate time is 30 minutes.

-Visit 2 (Basal): Your laboratory results are assessed and it is confirmed that you meet all the inclusion criteria, if so, cardiovascular studies will be carried out, which will consist in the measurement of pulse wave velocity and vascular index, and we will proceed with the randomization of your treatment, either enalapril or placebo (the treatment will be administered as a capsule of 5 mg, 30 minutes before each meal, twice a day for 90 days). A diary will be delivered describing the daily dose and, in addition, the medicine corresponding to the first month of the intervention will be delivered. Finally, general nutrition recommendations will be given since during the study it is not indicated to initiate

any type of diet to lose weight or an increase in physical activity. It will be cited 30 days after starting the treatment, date that will be recorded in your diary.

-Visit 3 and 4 (Follow-up): Day 30 and 60, the patient will be called to monitor the amount of medication ingested and the tolerability to treatment, and the medication corresponding to the current month will be provided.

-Visit 5 (End of the intervention period): After 90 days at the beginning of the intervention, you will be cited again and clinical and laboratory measurements will be taken in the same way as they were performed in the first visit.

-Visit 6 (Evaluation and delivery of results): Throughout 4 weeks will be scheduled to deliver and explain the results, including the initial and final, will also explain the evolution of each of them. Finally, a clinical summary will be made with medical-nutritional recommendations, as well as your constant medical surveillance if necessary.

Results during and at the end of the study and new information.

During the course of this study, we inform you of any new findings that are important in deciding whether to continue or stop the intervention. If we can provide you with new information, we will ask for your consent again to continue participating in this study in case of new interventions.

General risks of the intervention.

During the collection of the blood samples, one or more punctures (needle and syringe blood collection) will be required in one of the veins of your arms, during which you may feel pain and occasionally a bruise may form in the area where blood is taken. In order to avoid unnecessary risks for you and your family, the medicine given to you or prescribed in a strict manner must be consumed only by you and in the way it is indicated. You may have tiredness, weakness, low blood pressure, fainting, nausea, headache, diarrhea, muscle cramps, allergy, dry cough, swelling of the eyelids and tongue. It is important to mention that these may or may not be presented, due to the physical and genetic characteristics of each individual. It is essential that you inform the treating researcher in this investigation of all the discomfort that may occur throughout the study. The investigator is aware of all the discomforts that may occur and the best way to treat them, which may include the suspension of treatment or reduction of the dose, and based on

ARTICLE 14 OF THE REGULATIONS OF THE GENERAL LAW OF HEALTH IN MATTER OF RESEARCH, the attention to all the participants of this research will be 24 hours a day and in case of any adversity or amendment during the execution of the project, the researcher will inform the Research Ethics Committee of the Hospital Civil Juan I. Menchaca. There is availability of medical treatment and compensation to which the institution legally has rights in the case of damages that warrant directly caused by the investigation, in the same way if there are additional expenses, these will be absorbed by the research budget.

Benefits of the intervention.

You will not receive payment for your participation in this study, nor does this study involve expenses for you. You will benefit from an assessment that includes: weight, height, body mass index, percentage of body fat, blood pressure, waist circumference, laboratory determinations, in addition to receiving professional guidance about what measures you should take to decrease the risk of presenting complications. At the end of the study, nutritional follow-up and multidisciplinary support will be given to treat rheumatoid arthritis if necessary. The visits to the hospital, the procedures caused by the medication will not have any cost for you if necessary during the intervention. In addition, the results of this study will contribute to the advancement of scientific knowledge for new applications in the use of enalapril, as well as for the management of people like you who present the same problem.

Participation or withdrawal in the study.

Your participation in our study is completely voluntary. You may decide not to enter or discontinue your participation at any time during the study without penalty or loss of benefits. The investigator may also suspend your participation without your consent if you need additional treatment, do not follow the instructions or there is a suspicion that the medication is harmful to your health. However, it will still have the benefit of medical care and nutritional follow-up.

Privacy and confidentiality

The information that is provided to us (such as your name, telephone number and address), as well as your answers to the questionnaires and the results of your clinical and laboratory tests will be kept confidential to guarantee your privacy. No one else will have access to the information provided to us during your participation, unless you wish to do so and / or as necessary to protect your welfare and rights.

Dissemination of results

The results will be delivered confidentially to each of the participants as well as special measures that merit self-care or follow-up according to the results. The result of the study will be published in an indexed scientific journal and will be provided to each of the study participants.

Contact staff for questions and clarifications

If during the study, you have questions or doubts about this research study, you can communicate 24 hours a day with the principal investigator of the study.

Contact staff for questions and clarifications

If during the study, you have questions or doubts about this research study, you can communicate 24 hours a day with the principal investigator of the study.

WHO TO CONSULT.

For any doubt or clarification related to the treatment, handling of the patient, as well as the sample to know a specific or particular information related to it, you can contact the principal investigator:

Name: Dr. Mónica Vázquez del Mercado Espinosa
Email: dravme@hotmail.com
Cell number 3322232420/3325996852

Declaration of informed consent.

We have been clearly explained what this study consists of, in addition I have read and / or someone has read me the content of this consent. We have been given the opportunity to

ask questions and all our questions have been answered with clear language and fully satisfied our doubts, and they have also provided us with a copy of this format. By signing this consent, we acknowledge that we have been informed about the methods, study procedures, administration of the medication, as well as the benefits and adverse effects that may occur. I understand that we are free to withdraw from the study at any time with prior notice to the investigators of our reasons, without losing benefits or obtaining any penalty. Freely and without reservations, we give our consent to participate as a patient in this study.

Place: Instituto de Investigación en Reumatología y Sistema Músculo Esquelético, Centro Universitario de Ciencias de la Salud, Universidad de Guadalajara. Sierra Mojada No. 950. Colonia Independencia. CP. 44370. Tel. (33) 10585200. Ext. 33635.

Guadalajara, Jalisco, ______ /__________ /__________

___________________________________ _____________________ _____________________

Doctor or researcher who provides the Signature Date

Information and collect consent

______________________________ _____________________ _____________________

Full Patient Name Signature Date

Witness 1

Full Name Witness 1: _______________________

Address: ____

Relationship____

Date____________________ Sign____________________

Witness 2

Full Name Witness 2: ________________________

Address: ____

Rerationship____

Date____________________ Sign____________________

22.2 ATTACHED 2. DATA COLLECTION AND IDENTIFICATION SHEET.

Visit 1 (day -1)

CLINIC HISTORY

GENERAL DATA		
01	**ELABORATION DATE**	
02	**REGISTRY NUMBER**	
03	**FULL NAME**	
04	**BIRTHDATE**	
05	**CURRENT AGE**	
06	**GENDER**	
07	**CIVIL STATUS**	
08	**OCUPATION**	
09	**SCHOLARSHIP**	
10	**BIRTH PLACE**	
11	**CURRENT POPULATION**	
12	**COLONY**	
13	**STREET**	
14	**OUTDOOR NUMBER**	
15	**INTERIOR NUMBER**	
16	**CROSS WITH**	
17	**LANDLINE**	
18	**CELL PHONE**	
19	**EMAIL**	

20 HOW DO YOU CONSIDER YOUR CURRENT HEALTH STATUS? : GOOD ( ) REGULAR ( ) BAD ( )

FAMILY INHERITANCE BACKGROUND mark in the parenthesis only if you have the disease		
21	OBESITY/ OVERWEIGHT	Mom () Dad () Brothers () Sisters () Uncles father (), () Uncles mother, () Grandmother father () Grandfather father () grandmother mother () grandfather mother ()<br>Mom () Dad () Brothers () Sisters () Uncles father (), () Uncles mother, () Grandmother father () Grandfather father () grandmother mother () grandfather mother ()
22	TYPE 2 DIABETES MELLITUS	Mom () Dad () Brothers () Sisters () Uncles father (), () Uncles mother, () Grandmother father () Grandfather father () grandmother mother () grandfather mother ()
23	HBP	Mom () Dad () Brothers () Sisters () Uncles father (), () Uncles mother, () Grandmother father () Grandfather father () grandmother mother () grandfather mother ()
24	CVDs *	Mom () Dad () Brothers () Sisters () Uncles father (), () Uncles mother, () Grandmother father () Grandfather father () grandmother mother () grandfather mother ()
25	MI	Mom () Dad () Brothers () Sisters () Uncles father (), () Uncles mother, () Grandmother father () Grandfather father () grandmother mother () grandfather mother ()
26	CKD	Mom () Dad () Brothers () Sisters () Uncles father (), () Uncles mother, () Grandmother father () Grandfather father () grandmother mother () grandfather mother ()
27	CVA	Mom () Dad () Brothers () Sisters () Uncles father (), () Uncles mother, () Grandmother father () Grandfather father () grandmother mother () grandfather mother ()
28	CANCER	Mom () Dad () Brothers () Sisters () Uncles father (), () Uncles mother, () Grandmother father () Grandfather father ()

		grandmother mother () grandfather mother ()
29	HYPERCHOLESTEROLEMIA	Mom () Dad () Brothers () Sisters () Uncles father (), () Uncles mother, () Grandmother father () Grandfather father () grandmother mother () grandfather mother ()

***Includes atherosclerosis, angina. Venoues and arterial insufficiency**

PATHOLOGICAL PERSONAL HISTORY		
30	Consumption of medications	
31	TOBACCO	No. Of cigarrettes:<br>CONSUMPTION TIME:<br>Passive smoker:
32	ALCOHOLISM	FREQUENCY: QUANTITY:<br>CONSUMPTION TIME:
33	DRUG ADDICTION	
34	OBESITY/OVERWEIGHT	
35	TYPE 2 DIABETES MELLITUS	
36	HBP	
37	CVDs	
38	MI	
39	CKD	
40	CVA	
41	CANCER	
42	HYPERCHOLESTEROLEMIA	
43	OBSTETRICS BACKGROUND	G: D: C: A:
44	GYNECOLÓGICAL BACKGROUND	Menarca: RHYTHM: FUM:<br>HORMONE CONSUMPTION:
45	AR DIAGNOSIS DATE:	
46	DATE DISEASE ONSET	
47	CURRENT TREATMENT	

**48. INTERROGATORY FOR APPARATUS AND SYSTEMS: If positive, enclose in an oval**<br><br>**Headache - Dizziness / dizziness - Tinnitus - blurred vision - paresthesia - syncope - convulsions - bleeding / hematoma - skin pallor - brittle nails, alopecia - itching - muscle weakness - fatigue - joint / muscle / bone pain - joint stiffness - joint edema - anorexia - fever - night sweats - weight gain / loss - polydipsia - polyphagia - polyuria - tremor - palpitations - depressed - stress - lethargic - chest pain - exertional dyspnea - paroxysmal nocturnal dyspnea - orthopnea - upper or lower limbs edema - intermittent claudication / rest - cough - sputum - hemoptysis - abdominal pain - abdominal distension - dyspepsia - epigastric pain - tenesmus / urgency - postmiction drip - decrease in the Height of the jet - urinary incontinence - dysuria - hematuria - nocturia - change in intestinal habit / caliber change - pelvic pain - vaginal itching - waste**<br><br>**49. Description:**		
**PHYSICAL EXPLORATION**		
50	BP (mmHg)	RA: LA: A: 2: 3:<br>AVERAGE:
51	PULSE (X’)	
52	HR (X’)	
53	RR (X’)	
54	TEMPERATURE (°C)	
55	HEAD	Negative ( ) Positive:
56	FONDOSCOPY	Negative ( ) Positive:
57	OTOSCOPY	Negative ( ) Positive:
58	NECK	Negative ( ) Positive:
59	CHEST	Negative ( ) Positive:
60	CARDIAC AREA	Negative ( ) Positive:
61	ABDOMEN	Negative ( ) Positive:

62	LIMBS	Negative ( ) Positive:
63	CNS	Negative ( ) Positive:

ANTHROPOMETRY AND BIOELECTRIC IMPEDANCE		
64	WEIGHT (kg)	
65	BMI (kg/m^2)	
66	GREASY MASS(Kg)	
67	BODY FAT (%)	
68	TOTAL BODY WATER (kg)	
69	LEAN BODY MASS (kg)	
70	BONE MASS	
71	FAT MASS (kg/%):	
72	CHEST	
73	RIGHT ARM	
74	LEFT ARM	
75	RIGHT LEG	
76	LEFT LEG	
**ANTHROPOMETRY**		
77	HEIGHT (cm)	
78	WAIST CIRCUNFERENCE (cm)	
79	HIP CIRCUNFERENCE (cm)	
80	MEDIUM ARM CIRCUMFERENCE (cm)	
81	ABDOMINAL ADIPOSE PANICLE (mm)	
82	BICYCLE ADIPOSE PANICLE (mm)	
83	TRICYPTAL ADIPOSE PANICLE (mm)	
84	ADIPOSE SUB-ESCAPULAR PANICLE (mm)	
85	SUPRA-ILIACO ADIPOSE PANICLE (mm)	

86	ADIPOSE FEMORAL PANICLE (mm)	
87	SAGITAL DIAMETER (mm)	
88	CORON DIAMETER (mm)	

FOOD CONSUMPTION FREQUENCY

Indicate with a cross in the frequency column, the option that is considered closest to your reality. In the column on the right, record the number corresponding to the frequency of reported consumption.

Food group		Consumption (times per month)										
		90	60	30	20	16	12	8	4	2	0	
Vegetables	Natural											
	Processed											
Fruits	Natural											
	Processed											
Cereals and Tubers	Natural											
	Processed											
Legumes	Natural											
	Processed											
Animal origin	Natural											
	Processed											
Milk / Yogurt	Natural											
	Processed											
Oils and fats	Natural											
	Processed											
Sugars												
Junk Products												
Alcoholic Drinks												

EVALUATION OF THE DISEASES

Disease Activity Score – DAS 28

Evaluation date: ______________

Patient folio: ____

Case file number: ________________ RA number: __________________

Mark with an X the corresponding joint:

	Right		Left	
	**Tender**	**Swollen**	**Tender**	**Swollen**
Shoulder				
Elbow				
Wrist				
MCP 1				
MCP 2				
MCP 3				
MCP 4				
MCP 5				
Gaeslen				
PIP 1				
PIP 2				
PIP 3				
PIP 4				
PIP 5				
Knee				
**Total DAS**	**Tender**		**Swollen**	

1. Disease severity perceived by patients

Very well   Very bad

Line total Height (mm):________   Total to mark (mm): _________

2. Pain severity perceived by patients.

Very well   Very bad

Line total Height (mm):________   Total to mark (mm): _________

3. Severity perceived by doctor.

Very well   Very bad

Line total Height (mm):________   Total to mark (mm): _________

**Calculation DAS-28**	
ESR	
CRP	
NAT	
NAD	
NAT/NAD index	
DAS-28 ESR	
DAS-28 CRP	
CDAI	
SDAI	

HEALTH ASSESSMENT QUESTIONNAIRE (HAQ)

Puntuación:					
	During the last week, have you been able to..?	Without any difficulty	With some difficulty	With much difficulty	Unable to do
1	Dress yourself, including tying shoelaces and performing buttons?				
	Soap your head?				
2	Get up from a chair without armrests?				
	Get in and out of bed?				
3	Cut a steak?				
	Open a new milk carton?				
	Pour the drink?				
4	Walk outdoors on flat ground?				
	Climb five steps?				
5	Wash and dry your entire body?				
	Sit down and get up out of the toilet?				
	Take a shower?				
6	Take a 1 kg sugar packet from a shelf placed above your head?				
	Bend down to pick up clothing from the floor?				
7	Open a car door?				
	Open closed jars that had already been opened?				
	Open and close taps?				
8	Do delegations and purchases?				
	Get in and out of car?				
	Do household chores such as sweeping or washing dishes?				

Choose the activities in which you need help from another person:	Choose if you habitually use some of these tools:

__Dress and groom<br>__Get up<br>__Eat<br>__Walk<br>__Personal hygiene<br>__Reach<br>__Open and close things<br>__Delegations and households chores	__Wide handle cutlery<br>__Cane, crutches, walker or wheelchair<br>__Seat or special bar for the bathroom<br>__Tall seat for toilet<br>__Opener for previously opened jars

D A T A C O L L E C T I O N S H E E T

V i s i t 2 (d a y 0)

S T A R T O F I N T E R V E N T I O N

**Date:** I______I_______I_______I<br>_________________________	Initials:	**Randomization:**<br>I_____________I<br>**Register:**<br>I_____I_____I_____I

**Clinical data:**	**Paraclinical data:**
	**BP (mmHg):** I________I / I________I
**Weight:** I_________I **kg**	**CBP (mmHg):** I________I / I________I
**Height:** I_________I **m**	**% of body fat:** I_______I **%**
**BMI:** I__________I **kg/m^2**	**% of visceral fat:** I_______I **%**
**P:** I___________I **ppm**	**Waist circumference:** I_______I **cm**
**BR:** I___________I **bpm**	**Abdominal circumference:** I_______I **cm**

Additional comments:

____

____

____

____

____

____

Date to visit 3:__________________

V i s i t 3 (d a y 3 0 ± 2)

**Date:** I______I_______I_______I<br>_________________________	Initials:	**Randomization:** I_____________I<br>**Register:** I_____I_____I_____I

**Clinical data:**	**Paraclinical data:**
	**BP (mmHg):** I________I / I________I
**Weight:** I_________I **kg**	**CBP (mmHg):** I________I / I________I
**Height:** I_________I **m**	**% of body fat:** I_______I **%**
**BMI:** I__________I **kg/m^2**	**% of visceral fat:** I_______I **%**
**P:** I___________I **ppm**	**Waist circumference:** I_______I **cm**

**BR:** I___________I **bpm**	**Abdominal circumference:** I_______I **cm**
**Treatment adherence:**<br><br>**First dose date:**__________________________<br>**Last dose date:**<br>___________________________<br>**Has forgotten some dosage?:**<br>____________________<br>**When?:**<br>____<br>**Medication count:**<br>_________________________	**Tolerability and adverse effects:**<br><br>**Duration and adverse effect description:** ________________<br>____<br>____<br>____<br>____

Date to visit 4:___________________

V i s i t 4 (d a y 6 0 ± 2)

**Date:** I______I_______I_______I<br>__________________________	Initials:	**Randomization:** I_____________I<br>**Register:** I_____I_____I_____I

**Clinical data:**<br><br>**Weight:** I_________I **kg**<br>**Height:** I_________I **m**<br>**BMI:** I__________I **kg/m^2**<br>**P:** I___________I **ppm**<br>**BR:** I___________I **bpm**	**Paraclinical data:**<br>**BP (mmHg):** I________I / I________I<br>**CBP (mmHg):** I________I / I________I<br>**% of body fat:** I_______I **%**<br>**% of visceral fat:** I_______I **%**<br>**Waist circumference:** I_______I **cm**<br>**Abdominal circumference:** I_______I **cm**
**Treatment adherence:**<br><br>**First dose date:**__________________________<br>**Last dose date:**<br>___________________________<br>**Has forgotten some dosage?:**<br>____________________<br>**When?:**<br>____<br>**Medication count:**	**Tolerability and adverse effects:**<br><br>**Duration and adverse effect description:** ________________<br>____<br>____<br>____<br>____

______________________	

V i s i t 5 (d a y 9 0 ± 2)

<table>
<tr><td>Date: I______I_______I_______I Initials: ________________________</td><td>Randomization:
I_____________I
Register: I_____I_____I_____I</td></tr>
<tr><td>Clinical data:
Weight: I_________I kg
Height: I_________I m
BMI: I__________I kg/m^2
P: I___________I ppm
BR: I___________I bpm
BP (mmHg): I________I / I________I
CBP (mmHg): I________I / I________I
% of body fat: I_______I %
% of visceral fat: I_______I %
Waist circumference: I_______I cm
Abdominal circumference: I_______I cm
Basal glucose: I__________I mg/dl
HDL-C: I__________I mg/dl
LDL-c: I__________I mg/dl
TC: I__________I mg/dl
Triglycerides: I__________I mg/dl</td><td>Paraclinical data:
Uric acid:_____________mg/dl
Creatinine:___________ mg/dl
TGO:________________U/l
TGP:________________ U/l
PWV________________m/s</td></tr>
<tr><td colspan="2">Additional commentaries:
____
____
____
______________________________</td></tr>
</table>

22.3 ATTACHED 3. DAILY MONITORING AND PATIENT FOLLOW-UP

Research project:

Effect of enalapril use in arterial stiffness in rheumatoid arthritis patients.

**Patient name:**	**Randomization:** l_____l_____l_____l<br><br>**Register:** l_____l_____l_____l
**Start date:** l________l________l________l	**Finish date:** l________l________l________l
**Responsible researcher:**<br>**Mónica Vázquez del Mercado Espinosa, MD, PhD / Felipe de Jesús Pérez Vázquez. MD**<br>**3322232420 / 3325996852**	

REMEMBER:

1.- Wear light clothes for weight and Height.

2- Bring your treatment diary to appointments.

3.- Bring the bottle of capsules that we gave you for change it for another with new treatment.

How to fill the diary?

- **Appointment record:**
  - Here you register the dates of your evaluations.
- **Treatment record:**
  - You have to register dates and hours of the intake medication.

- **Observations or commentaries:**
  - Any discomfort related to the drug intake, please report with your responsable doctor
  - You have to register any disconfort as well sickness and/or treatment that occurs during the research period, including if you think is not related with the drug. Also register if was necessary the intake of another drug besides enalapril.
  - Here you write changes in healthy state as well if you had some extraordinary evento either physical activity or diet.

Appointmen record

Visit	Date	Hour	Indication
**1 Start**		8 am	Fast

**2 tracking**	8 am	Bring your jar<br>Bring your diary
**3 tracking**	8 am	Bring your jar<br>Bring your diary
**4 study's end**	8 am	Fast<br>Bring your diary
**5 results**	8 am	

Capsules intake record

Date ___/___/____ at ___/___/____ **# of capsule delivered in the month: _________**

Week ___/___/____ at ___/___/____

Intake register before foods

**Flask**	**Day**	**DIAL**		✓ ×	**Observations/ Comments***
		Before breakfast		Before dinner	
___	1				
___	2				
___	3				
___	4				
___	5				
___	6				
___	7				
___	8				

capsules you forgot to take: _________

Commentaries:

Capsules intake record

Date ___/___/____ at ___/___/____ # of capsule delivered in the month: _________

Week ___/___/____ at ___/___/____

Intake register before foods

**Flask**	**Day**	**DIAL**		✓	×	**Observations/ Comments***
		Before breakfast		Before dinner		
___	9					
___	10					
___	11					
___	12					
___	13					
___	14					
___	15					
___	16					

capsules you forgot to take: _________

Commentaries:

Capsules intake record

Date ___/___/____ at ___/___/____ # of capsule delivered in the month: _________

Week ___/___/____ at ___/___/____

Intake register before foods

Flask	Day	DIAL		✓ ✗	Observations/ Comments*
		Before breakfast		Before dinner	
___	17				
___	18				
___	19				
___	20				
___	21				
___	22				
___	23				
___	24				

capsules you forgot to take: _________

Commentaries:

Capsules intake record

Date ___/___/____ at ___/___/____ # of capsule delivered in the month: _________

Week ___/___/____ at ___/___/____

Intake register before foods

Flask	Day	DIAL		✓	×	Observations/ Comments*
		Before breakfast		Before dinner		
___	25					
___	26					
___	27					
___	28					
___	29					
___	30					
___						
___						

capsules you forgot to take: _________

Commentaries:
