# Supplementary material for: Enalapril Influence on Arterial Stiffness in Rheumatoid Arthritis Women: A Randomized Clinical Trial
Source: Front Med (Lausanne). 2020 Jan 29;6:341. doi: 10.3389/fmed.2019.00341 (PMC7025600; doi:10.3389/fmed.2019.00341)
Supplement: Supplementary Data Sheet 2 — Trial Protocol Spanish language. [file Data_Sheet_2.PDF]

UNIVERSIDAD DE GUADALAJARA

CENTRO UNIVERSITARIO DE CIENCIAS DE LA SALUD/
SECRETARÍA ACADÉMICA / COORDINACIÓN DE INVESTIGACIÓN

CI/006/2017

DICTAMEN DE EVALUACIÓN

En la ciudad de Guadalajara, Jalisco a los veintitrés días del mes de enero de dos diecisiete, en las instalaciones del Centro Universitario de Ciencias de la Salud de la Universidad de Guadalajara, se reunieron los miembros de los Comités de Ética, Investigación y Bioseguridad para dictaminar sobre las evaluaciones emitidas por sus pares del proyecto con número de registro **CI-0117: "EFECTO DEL TRATAMIENTO PROFILÁCTICO CON MALEATO DE ENALAPRILO EN LA RIGIDEZ ARTERIAL EN PACIENTES CON ARTRITIS REUMATOIDE "** del cual es responsable el (la) ***Dr (a). en C. Monica Vazquez del Mercado Espinosa.***

Los miembros de los comités acordaron que de acuerdo a las evaluaciones emitidas por sus pares, el proyecto presenta los requisitos necesarios en cuanto a su presentación, calidad y contenido por lo que se **APRUEBA** para su realización.

ATENTAMENTE

COMITÉ DE ÉTICA EN INVESTIGACIÓN

Dra. Martha Villaseñor Farías
Presidente

Dr. en C. Noe Alfaro Alfaro
Secretario

COMITÉ DE INVESTIGACIÓN

Dr. en C Jorge R Padilla Gutierrez
Presidente

Dr. en C. Andrés Palomera Chávez
Secretario

COMITÉ DE BIOSEGURIDAD

Dr. Ma. de la Luz Galván Ramírez
Presidente

Dra. Celia Guerrero Velázquez
Secretario

Vo. Bo.

Dr. Adrián Daneri Navarro
Coordinador de Investigación

SALUD

JALISCO
GOBIERNO DEL ESTADO

1767

Dirección General de Salud Pública
Oficio No. DGSP/DDI/D.INV. 28/18
Guadalajara, Jalisco.,
Asunto: Incorporación de Proyecto al Registro Estatal de Investigación

17 ABR 2018

Dra. Mónica Vázquez del Mercado Espinosa
OPD Hospital Civil de Guadalajara
"Dr. Juan I. Menchaca"
Ciudad

Por este conducto hago propicia la ocasión para enviarle un cordial saludo deseándole éxito en sus actividades; y a la vez me permito comunicar a usted que se recibió en el Departamento de Investigación el Protocolo: **"Efecto del uso de enalapril en la rigidez arterial en pacientes con artritis reumatoide"**. Para realizarse bajo su responsabilidad y como investigadores asociados: Felipe de Jesús Pérez Vázquez y José de Jesús Gómez Bañuelos.

Por la documentación presentada, con los dictámenes de aprobación del Comité de Investigación y Ética en Investigación, del Hospital Civil de Guadalajara "Dr. Juan I. Menchaca" y el Departamento de Investigación con el asesor correspondiente, se concluye que el proyecto cumple con los criterios metodológicos, éticos y de originalidad, lo anterior en cumplimiento a la Norma Técnica 313 para el Registro y Seguimiento en Materia de Investigación en Salud (D.O.F. 25/07/1988), motivo por el cual se ha incorporado al Registro Estatal de Investigación con el número 13/HCJIM-JAL/2018.

Sin otro particular reitero un saludo y me despido como su atento y seguro servidor.

A t e n t a m e n t e
Sufragio Efectivo. No Reelección
"2018, Centenario de la Creación del municipio de Puerto Vallarta y del XXX Aniversario del Nuevo Hospital Civil de Guadalajara"

GOBIERNO DE JALISCO
PODER EJECUTIVO
SERVICIOS DE SALUD
JALISCO
CORRESPONDENCIA Y ARCHIVO

Dr. Antonio Luévanos Velázquez
Director de Desarrollo Institucional

/ JGRR / MGPC / yadi*

Secretaría de Salud
GOBIERNO DEL ESTADO DE JALISCO

SECRETARIA DE SALUD JALISCO
DIRECCIÓN GENERAL DE SALUD PÚBLICA
DIRECCIÓN DE DESARROLLO INSTITUCIONAL

R-INV-01

1) No. de Registro Estatal: 0211/18 HCJIM/2018
2) No. de Solicitud de Registro Estatal:
3) Fecha de Registro Estatal: 15 de febrero de 2018

4) Institución que solicita el Registro Estatal de Investigación: OPD Hospital Civil de Guadalajara "Dr. Juan I. Menchaca"
5) Unidad (Región, Hospital, Dirección): Salvador Quevedo y Zubieta No. 750 Col. Independencia , C.P. 44340 Guadalajara, Jalisco México

6) Título: Efecto de uso de enalapril en la rigidez en pacientes con artritis reumatoide

7) Duración prevista

	día	mes	año
8) Inicio	1	3	2018
9) Terminación	31	3	2019

10) Institución donde se realizará la investigación: OPD Hosptial Civil de Guadalajara "Dr. Juan I. Menchaca"
11) Región, unidad y/o Departamento donde se desarrollará la Investigación: OPD Hospital Civil de Guadalajara "Dr. Juan I. Menchaca"
12) Unidad o Departamento donde se desarrollará la investigación: Piso 6 Medicina Interna Reumatología
13) Domicilio de la Unidad: Salvador Quevedo y Zubieta No. 750 Co. Independencia CP 44340 Guadalajara., Jalisco México

II.- DATOS DEL INVESTIGADOR PRINCIPAL

14) Nombre: Dra. Mónica Vasquez Del Mercado Espinosa
15) Lugar de adscripción:
17) Horario laboral: 15:00 pm. a 22:30 hrs.
16) Teléfono/Fax trabajo:
17) Teléfono Particular: 3339561304
18) Correo electrónico: dravme@hotmail.com

III.- TIPO DE INVESTIGACIÓN: de acuerdo a la metodología utilizada (marque con una cruz)

20) Según Objetivos	21) Según Metodología	22) Según el Sujeto de Estudio		23) Según Riesgo
Básica	Exploratoria	Biomédica	Educativa	■ Sin Riesgo
■ Aplicada	■ Comparativa	■ Clínica	Otra	■ Con Riesgo Mínimo
Tecnológica	Experimental	Salud Pública		Con Riesgo Mayor que el Mínimo

IV.- DISCIPLINAS COMPRENDIDAS EN EL PROYECTO:

24) otras disciplinas que participan

V.- ORIGEN DE LA INICIATIVA

25) Interno ■ 26) Externo 27) Interno / Externo

VI.- TESIS DE GRADO: 28) Para obtener el título de: Línea de Investigación
29) Universidad y Escuela Universidad de Guadalajara

VII.- APOYO EXTERNO: (recursos, información, asesoría, otros)

30) Institución	31) Tipo de apoyo que otorga

VIII.- INVESTIGADORES: Datos del investigador principal y máximo 4 investigadores asociados en orden de participación.

32) NOMBRE COMPLETO	33) PUESTO / FUNCIÓN EN EL PROYECTO	34) HRS/SEM DEDICA AL	35) MÁXIMO GRADO
Dra. Mónica Vázquez del Mercado Espinosa	Investigador Principal	10 hrs. Por semana	Doctorado
MCP Felipe de Jesús Pérez Vázquez	Colaborador	08 hrs por semana	Médico Cirujano y Partero
Dr. José de Jesús Gómez Bañuelos	Colaborador	08 hrs por semana	Doctorado
			Técnica

IX.- Número de otros participantes 36) Apoyo Técnico: 37) Apoyo Administrativo 38) Total: 3

X.- PROTOCOLO AUTORIZADO POR:

39) Comité de Investigación ■ 40) Comité de Ética ■ 41) Director de la unidad 42) Otro divisional

43) SE CONCLUYO EL PROYECTO Si No
44) FECHA DE TERMINACIÓN 31/ 3/2019 día mes año
45) INFORME PARCIAL SI NO
46) INFORME FINAL SI NO

PRODUCCIÓN CIENTIFICA DEL PROYECTO: (anexar fotocopias)

NOMBRES Y FIRMAS

Dra. Mónica Vázquez del Mercado Espinosa	Dr. Dante Daniel Hernández Colin	
**Firma del Investigador Principal**	**Director**	**Coordinador de Desarrollo Institucional**

OPD. HOSPITAL CIVIL DE GUADALAJARA DR. JUAN I. MENCHACA
SUBDIRECCIÓN DE ENSEÑANZA E INVESTIGACIÓN
ADMINISTRACIÓN 2016 - 2019

Fuente: Reglamento de la Ley General de Salud en Materia de I
Normas Técnicas 313, 314, y 315

El día 27 de julio de 2017, en la Ciudad de Guadalajara se reúne el Comité de Investigación del OPD Hospital Civil de Guadalajara "Dr. Juan I. Menchaca" para la revisión del protocolo de Investigación: **"Efecto del uso de enalapril en la rigidez arterial en pacientes con artritis reumatoide"** presentado por la Dra. Mónica Vázquez del Mercado Espinosa, estudio para línea de investigación teniendo como colaboradores al Médico Cirujano y Partero Felipe de Jesús Pérez Vázquez y al Dr. José de Jesús Eduardo Gómez Bañuelos.

Dictamen: Aprobado

COMITÉ DE INVESTIGACIÓN
OPD HOSPITAL CIVIL DE GUADALAJARA DR. JUAN I. MENCHACA

Dr. J. Jesús Pérez Molina
Presidente del Comité de Investigación

OPD HOSPITAL CIVIL DE
GUADALAJARA DR. JUAN I. MENCHACA

EVALUACIÓN DE DOCUMENTOS DE PROTOCOLOS ACTIVOS

Investigador: Dra. Mónica Vázquez del Mercado Espinosa
Colaboradores: MCP Felipe de Jesús Pérez Vázquez,
Dr. José de Jesús Eduardo Gómez Bañuelos

Titulo del Proyecto :

Efecto del uso de enalapril en la rigidez en pacientes con artritis reumatoide

Documentación evaluada

- Protocolo de Investigación
- Carta de Consentimiento Bajo Información
- Hoja de identificación recolección de datos (historia clínica)
- Hoja de Frecuencia de alimentos
- Hoja de recolección de datos (visitas)
- Diario de monitoreo y seguimiento del paciente

Fecha de este dictamen: 08 febrero de 2018

DICTAMEN :

Aprobado

Dr. Fernando Moreno Ávila
Presidente del Comité de Ética en Investigación

Salvador Quevedo Y Zubieta No. 750 C.P. 44340 Guadalajara Jalisco, México Tel: Conmutador: 36-18-93-26, 36-18-93-62

UNIVERSIDAD DE GUADALAJARA

NUEVO HOSPITAL CIVIL DE GUADALAJARA

“DR. JUAN I. MENCHACA”

“EFECTO DEL USO DE ENALAPRIL EN LA RIGIDEZ ARTERIAL EN PACIENTES CON ARTRITIS REUMATOIDE”

Investigador responsable: Dra. Mónica Vázquez del Mercado Espinosa

Guadalajara, Jalisco, Enero 2017

UNIVERSIDAD DE GUADALAJARA

NUEVO HOSPITAL CIVIL DE GUADALAJARA

"DR. JUAN I. MENCHACA"

Propuesta de protocolo

"EFECTO DEL USO DE ENALAPRIL EN LA RIGIDEZ ARTERIAL EN PACIENTES CON ARTRITIS REUMATOIDE"

Investigador responsable: Dra. Mónica Vázquez del Mercado Espinosa

Guadalajara, Jalisco, Enero 2017

“EFECTO DEL USO DE ENALAPRIL EN LA RIGIDEZ ARTERIAL EN PACIENTES CON ARTRITIS REUMATOIDE”

Índice.

Título. 6

Investigadores. 6

Sede. 6

1. Marco Teórico. 7

1.1 Artritis reumatoide. 7

1.1.1 Epidemiología de la AR. 7

1.1.2 Etiología de la AR. 7

1.1.3 Fisiopatología de la AR. 8

1.1.4 Morbilidad y mortalidad en AR. 9

1.1.5 Rigidez Arterial en Artritis reumatoide. 10

1. 2 Velocidad de onda de pulso. 10

1.3 Maleato de enalaprilo. 12

1.3.1 Estructura química del maleato de enalaprilo. 12

1.3.2 Farmacocinética y metabolismo. 13

1.3.3 Farmacodinamia. 13

1.3.4 Mecanismo de acción. 14

1.3.5 Indicaciones del enalapril. 15

1.3.6 Efectos adversos. 15

1.3.7 Hallazgos en las pruebas de laboratorio. 15

2. Antecedentes. 17

3. Justificación. 18

4. Planteamiento del problema. 19

4.1 Pregunta de Investigación. 19

5. Hipótesis. 20

5.1 Hipótesis Alterna. 20

5.2 Hipótesis Nula. 20

6. Objetivos. 21

6.1 Objetivo general. 21

6.2 Objetivos específicos. 21

7. Materiales y métodos. 22

7.1 Diseño del estudio. 22
7.2 Universo del estudio. 22
7.3 Lugar de trabajo. 22
7.4 Intervención y seguimiento. 22
7.4.1 Selección o asignación de los sujetos de estudio a los grupos de estudio. 23
8. Tamaño de muestra. 24
9. Criterios de selección. 25
9.1 Criterios de inclusión. 25
9.2 Criterios de no inclusión. 25
9.3 Criterios de eliminación. 25
10. Variables. 27
10.1 Independientes. 27
10.2 Dependientes. 27
10.3 Intervinientes. 27
10.4 Definición de las variables. 27
10.5 Operacionalización de variables. 29
11. Grupo de estudio. 31
11.1 Asignación del medicamento de la intervención y cegamiento. 31
12. Determinaciones clínicas. 32
13 Determinación de laboratorio. 35
13.1 Obtención de muestras biológicas. 35
14. Análisis estadístico. 37
15. Descripción del estudio. 37
15.1 Desarrollo de la fase de campo. 37
15.2 Adherencia terapéutica. 39
15.3 Criterios de seguridad. 39
15.4 Recolección de datos. 39
15.5 Difusión de los resultados finales de la investigación. 39
16. Aspectos éticos. 40
17. Financiamiento. 41
18. Recursos humanos. 41
18.1 Equipo de Trabajo. 41
19. Recursos materiales. 43

20. Conflictos de interés. 43
21. Referencias bibliográficas. 44
22. ANEXOS. 48
22.1 ANEXO 1. CARTA DE CONSENTIMIENTO BAJO INFORMACIÓN 48
22.2 ANEXO 2. HOJA DE IDENTIFICACIÓN Y RECOLECCIÓN DE DATOS. 55
22.3 ANEXO 3. DIARIO DE MONITOREO Y SEGUIMIENTO DEL PACIENTE. 69

Título.

“EFECTO DEL USO DE ENALAPRIL EN LA RIGIDEZ ARTERIAL EN PACIENTES CON ARTRITIS REUMATOIDE”.

Investigadores.

- **Investigador Principal.** Dra. En C. Mónica Vázquez del Mercado Espinosa.
- **Investigador Colaborador.** Dr. En C. José de Jesús Eduardo Gómez Bañuelos.
- **Investigador Colaborador.** Dr. Felipe de Jesús Pérez Vázquez.

Sede.

- **Servicio de Reumatología**, División de medicina Interna del Nuevo Hospital Civil de Guadalajara, “Dr. Juan I. Menchaca”.
- **Instituto de Investigación en Reumatología y del Sistema Musculo-esquelético**, Departamento de Biología Molecular, Centro Universitario de Ciencias de la Salud, Universidad de Guadalajara.

1. Marco Teórico.

1.1 Artritis reumatoide.

La artritis reumatoide (AR) es una enfermedad inflamatoria crónica sistémica [1] que se diferencia por la artritis crónica, erosiva de las articulaciones sinoviales. Sus síntomas principales son: dolor, fatiga, rigidez matinal e incapacidad funcional. Se caracteriza por la presencia de auto-anticuerpos como factor reumatoide (FR) y anticuerpos contra proteínas citrulinadas (ACPA) [1, 2].

1.1.1 Epidemiología de la AR.

La AR afecta al 0.5% a 1% de la población mundial [3, 4] con una relación mujer : hombre 3:1 siendo la quinta década de vida la edad de inicio más frecuente [5]. En México existe una prevalencia general reportada del 1.6%, aunque existe una gran variación en la epidemiología del país de acuerdo a la región estudiada, en el estudio de Peláez Ballestas del 2011 sobre la epidemiología de enfermedades reumáticas en México encontró que las variaciones geográficas sugieren un papel para los factores geográficos en la prevalencia de enfermedades reumáticas [6-8].

1.1.2 Etiología de la AR.

La AR es una enfermedad multifactorial, en la cual aún permanece indefinida su etiología exacta, sin embargo, se considera que el desarrollo de la enfermedad está relacionado con la predisposición genética y la exposición a factores medioambientales desencadenantes [9, 10]. El factor de riesgo genético más importante para el desarrollo de AR es la secuencia conocida como epítope compartido (EC), presente en los diferentes alelos del HLA-DRB1, que puede encontrarse hasta en un 78% de los pacientes con AR [11]. Esta variación genética favorece la presentación antigénica a los linfocitos T de los péptidos citrulinados [12]. Por otra parte, dentro de los factores medioambientales desencadenantes, el tabaquismo es considerado como el factor de

riesgo para el desarrollo con mayor influencia para el desarrollo de AR. Padyukov[13], en su estudio del 2004 observó una interacción entre genes y medio ambiente entre el tabaquismo y los genes compartidos del epítrope de HLA-DR proporcionando un alto riesgo de artritis reumatoide seropositiva. Posteriormente, Lundström [14], en su estudio del 2009 encuentra una fuerte interacción entre los distintos alelos DRB1 y el tabaquismo confiriendo un mayor riesgo en el desarrollo de la AR con ACPA positivos. Adicionalmente, se ha propuesto que la inhalación de humo de tabaco está relacionada con aumento en la expresión de la enzima peptidil-arginina-deaminasa (PAD) que genera el incremento a nivel pulmonar de péptidos citrulinados [15]. Por otra parte, la desregulación hormonal en el sexo femenino se asocia con un mayor riesgo para desarrollar AR; siendo mayor su prevalencia en el periodo post-parto y menopausia [16].

1.1.3 Fisiopatología de la AR.

A pesar de múltiples esfuerzos para desenmascarar los mecanismos de iniciación y propagación de la AR no ha sido posible definirlos completamente, sin embargo, la autoinmunidad y el proceso inflamatorio crónico tienen un papel central en la patogenia de la AR. Encontrándose en dichos mecanismos la participación de linfocitos T cooperadores (T_H) y linfocitos B (LB) con la generación de auto-anticuerpos [17]. La participación de los linfocitos T_H es por medio de la producción de citocinas pro-inflamatorias como el factor de necrosis tumoral alfa (TNF-α) y la interleucina 6 (IL-6), las cuales se han encontrado en mayor abundancia en estudios anteriores, siendo considerado el TNF-α como la citocina central en la patogenia en los mecanismos de daño articular y la IL-6 como principal activadora del endotelio y de osteoclastos [17]. Por otra parte, la generación de auto-anticuerpos por los linfocitos B, siendo los más descritos el factor reumatoide (FR) y los anticuerpos contra proteínas citrulinadas conocidos por sus siglas como ACPA[18] son anticuerpos que están dirigidos contra proteínas que han sufrido una modificación postraduccional conocida como citrulinación, en el que la conversión de la peptidil-arginina a peptidil-citrulina es catalizada por la familia de enzimas peptidilarginina deiminasa (PAD)[19]. La descripción de los ACPA y su presencia en el suero de los pacientes ha sido una prueba útil para el diagnóstico de AR con un alta especificidad para el diagnóstico del 95% [20] y un predictor de gravedad de la enfermedad [21, 22]. Con lo anterior, sabemos que el proceso inflamatorio sinovial es complejo con la participación de

múltiples mecanismos inmunológicos con participación simultanea [23] generando daño articular [2] con manifestaciones clínicas como dolor, inflamación y rigidez en las articulaciones periféricas [24] principalmente en las articulaciones interfalángicas proximales, metacarpofalángicas y carpos; seguido de las metatarsofalángicas, tobillos, hombros, codos[25] y clínica extraarticular en el 15% de los pacientes como vasculitis, neuropatía, pericarditis, pleuritis y escleritis [24].

1.1.4 Morbilidad y mortalidad en AR.

Frecuentemente la AR compromete otros órganos distintos a las articulaciones y es considerada como un factor de riesgo cardiovascular independiente [26] asociado con una mayor letalidad que la población en general [27]. Estudios previos han demostrado que la AR reduce la esperanza de vida en tasas de mortalidad estandarizadas entre 1.14 y 2.24 [28]. En el 2003, Salomón [27] y colaboradores describieron el seguimiento de 114,342 mujeres sanas con edad promedio de 30-55 años durante 19 años (1977-1996) entre las que 527 tenían AR. En este estudio se encontró que las pacientes con AR tuvieron mayor mortalidad que los sujetos sanos con una relación de mortalidad de 2: 1.Crowson [29] en el 2005 comparó la proporción del riesgo para el desarrollo de la insuficiencia cardíaca (IC) y la enfermedad cardíaca isquémica (CI) atribuible a factores de riesgo cardiovasculares tradicionales entre los sujetos con y sin AR. En él se observó que el exceso de riesgo de IC y CI en los pacientes con AR no se explica por un aumento de la frecuencia o el efecto de factores de riesgo cardiovascular siendo estos factores atribuibles en un 54% en sujetos con AR en comparación de un 77% en sujetos sin AR. Adicionalmente, Kremers [30] en su estudio del 2008 estima el riesgo absoluto de 10 años de eventos cardiovasculares (CV) en la artritis reumatoide de recién diagnóstico y la contribución potencial de los factores de riesgo CV a la evaluación del riesgo absoluto, en el encuentra un riesgo del 16.8% de las enfermedades CV en sujetos con AR sin factores de riesgo CV de recién diagnóstico y un aumento al 60,4% en presencia de factores de riesgo como el tabaquismo, la hipertensión, la dislipemia, la diabetes y la obesidad.

En un estudio posterior, Crilly y colaboradores [31] en el 2009 describieron una relación dosis-respuesta entre la carga inflamatoria acumulativa y la rigidez arterial acumulada en la artritis reumatoide independientemente de los factores de riesgo establecidos. En este estudio se reporta que en 114 pacientes con AR (93 mujeres y 21

hombres) con edad promedio de 53.7 años (40-65) y una duración de la enfermedad de 9.6 (4.4-16.9) se encontró un mayor riesgo cardiovascular con respecto a su evaluación cardiológica por medio de la medición del índice de aumentación, siendo este de un valor promedio de 31.5; lo que sugirió que la afectación cardiovascular de los pacientes con AR está asociada con el aumento de la rigidez arterial, resultados similares reportados por Klocke en el 2003 [32] y posteriormente por Park en el 2012 [33].

1.1.5 Rigidez Arterial en Artritis reumatoide.

La rigidez arterial es definida por Kozakova M (2015) como: “La capacidad reducida de una arteria para expandirse o contraerse en respuesta a cambios de presión, resultado de la remodelación vascular que produce cambios en el balance de colágeno y elastina en la pared de los vasos sanguíneos” que es generada por el envejecimiento biológico, la arteriosclerosis, la disfunción endotelial, la inflamación crónica, el daño acumulado de alta tensión mecánica, la dislipidemia, el tabaquismo, la obesidad, la diabetes mellitus y la activación vascular.

En AR, la rigidez arterial es generada principalmente por factores relacionados con la enfermedad acelerada por el género y la actividad de la enfermedad [34] el tiempo de evolución de la enfermedad y niveles circulantes de proteína C-reactiva[35] la presencia de auto-anticuerpos [36] y citocinas circulantes, principalmente factor de necrosis tumoral alfa (TNF a) [37, 38] e interleucina-6 (IL-6) [35] Estos factores contribuyen a la perdida de la función endotelial [39] inhibición de su capacidad vasodilatadora y pérdida de la elasticidad arterial que afectan al sistema renina-angiotensina-aldosterona (RAA) y moléculas vasodilatadoras como la bradicinina[40] siendo esta última reportada por vez primera por el grupo Erspamer en 1960 como el producto final del sistema de calicreína-quinina en el sistema sanguíneo de mamíferos [41].

1. 2 Velocidad de onda de pulso.

Estudios previos han demostrado que la elasticidad arterial puede ser medida por medio de la velocidad de la onda del pulso central (VOPcf); la cual, relaciona el riesgo cardiovascular mediante la evaluación de la distensibilidad arterial[42] Actualmente, la

VOPcf es considerada como el estándar de oro para la evaluación de la rigidez arterial[43] y junto al incremento del índice de aumentación central (AIC) [42] corroboran la reducción de la elasticidad ocasionada por los cambios estructurales en las paredes de las arterias principales de conducción sanguínea [35, 44] considerando el aumento de 1 m/s sobre el valor normal de la VOP acorde a la edad el equivalente a un aumento del 12% de riesgo cardiovascular esto observado en el estudio de Ali R Khoshdel del 2007.

**Distribución de la VOP (m/s) acorde a la edad en sujetos sanos (1455)**	
Categoría de Edad (años)	Media
<30	6.2
30-39	6.5
40-49	7.2
50-59	8.3
60-69	10.3
>70	10.9

Cuadro 1. Valores estándar de VOPcf según edad. Sociedad Europea de Cardiología. European Heart Journal 2010

La VOPcf se determina a intervalos de dos, primero la colocación del tonómetro en la arteria carótida común, mientras la realización simultánea del electrocardiograma (ECG), después se repite el procedimiento en la arteria femoral. El retardo de tiempo pertenece a la demora del aumento de la onda de pulso distal a la R perteneciente al complejo QRS del ECG del segundo tiempo y el retardo de aparición de la onda de pulso proximal a la onda R que pertenece al complejo QRS del ECG del primer tiempo. La VOPcf se obtiene por varios instrumentos de medición, uno de ellos conocido como PulsePen, instrumento de medición por tonometría no invasiva con el dispositivo de la pluma de pulso (DiaTecne s.r.l., Milán, Italia) con una técnica que ha sido validado previamente y mostró eficacia para medir VOP. La VOP se calcula: VOP = distancia entre

dos segmentos arteriales / retardo de tiempo y sus unidades de medida son metros / segundo (m/s) [45].

Además de la medición de la VOP se propone un nuevo dispositivo llamado pOpmétre, el cual mide la rigidez arterial utilizando fotodiodos de infrarrojos en el dedo de la mano y el dedo del pie. La medición de la VOP por pOpmétre es en función de la talla del paciente y la segunda derivada de la diferencia del tiempo entre el pulso de cada dedo. Por otra parte es posible evaluar la rigidez arterial y la ateriosclerosis por medio del Índice Vascular Corazón-Tobillo, conocido por sus siglas en inglés como CAVI (Cardio-Ankle Vascular Index).El CAVI es una herramienta de medición oscilométrica, es un método de exploración arteriosclerótica para calcular la función vascular normal independiente de la presión arterial, mide la tensión arterial en las cuatro extremidades.

$$CAVI = [\ln Ps/Pd] \cdot \frac{2\rho}{\Delta P} \cdot PWV^2 \quad \cdots (3)$$

Ps : Presión sistólica   Pd : Presión diastólica
PWV : Velocidad de la onda de pulso entre el corazón y el tobillo
ρ : Densidad de la sangre ΔP : Presión del pulso

$$\beta = [\ln Ps/Pd] \cdot [D/\Delta D] \quad \cdots (1)$$

Ps : Presión sistólica   D : Diámetro
Pd : Presión diastólica ΔD : Cambio de diámetro

El índice CAVI se calcula mediante el parámetro de rigidez B obtenido a partir de la ecuación de Bramwell-Hill. El parámetro B se utiliza para diagnosticar el grado de esclerosis de la arteria carótida, a partir de la variación del diámetro y la tensión arterial media mediante ecografía ultrasónica.

1.3 Maleato de enalaprilo.

El Maleato de enalaprilo es un inhibidor de la enzima de conversión de la angiotensina (ECA). El enalapril, es un pro-fármaco del maleato de enalaprilo diseñado para su administración oral.

1.3.1 Estructura química del maleato de enalaprilo.

Fórmula   C20H28N2O5

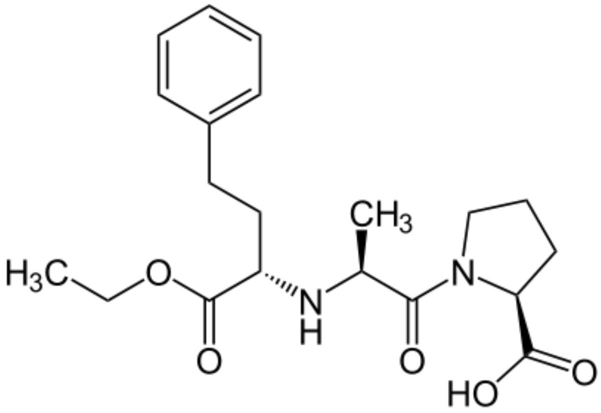

1.3.2 Farmacocinética y metabolismo.

Después de la administración oral del enalapril se observan unas concentraciones séricas máximas en 1 hora. A partir de los datos de la excreción urinaria, se deduce que el enalapril se absorbe en un 60% aproximadamente. La absorciòn del enalapril no es afectada por la presencia de alimento en el tracto digestivo. Una vez absorbido, el enalapril se hidroliza a enalaprilato, el verdadero inhibidor de la ECA. Las concentraciones máximas de enalaprilato se alcanzan unas 4 horas después de una dosis oral de enalapril. La cantidad de fármaco unida no aumenta con la dosis, lo que indica que el punto de fijación es saturable. La semi-vida de eliminación efectiva, determinada a partir de datos cinéticos después de dosis orales múltiples es de unas 11 horas. La excreción del enalapril es sobre todo renal. Aproximadamente el 94% de la dosis administrada se recupera de la orina o las heces como enalaprilato o enalapril.

1.3.3 Farmacodinamia.

La administración de enalapril a pacientes con hipertensión ligera a moderada ocasiona la reducción de la presión arterial tanto en posición supina como de pie, sin que se observe un componente ortostático. La hipotensión postural sintomática es infrecuente, aunque puede darse en pacientes con depleción de volumen.

En la mayoría de los pacientes estudiados, después de una dosis oral de enalapril, el inicio del efecto antihipertensivo se observa una hora después de la administración, produciéndose la máxima reducción de la presión arterial a las 6 horas. A las dosis

recomendadas el efecto antihipertensivo se mantiene al menos durante 24 horas, aunque en algunos pacientes hay que esperar algunas semanas para que se alcanze la reducción óptima de la presión arterial. Los efectos antihipertensivos del enalapril se mantienen durante la administración crónica del fármaco y no se observado efectos de rebotes hipertensivos cuando se discontinuado la medicación de forma abrupta.

1.3.4 Mecanismo de acción.

El enalapril oral, después de ser hidrolizado a enalaprilato, inhibe la enzima de conversión de la angiotensina (ECA) tanto en el hombre como en los animales de experimentación. La ECA es una peptidil-dipeptidasa que cataliza la conversión de la angiotensina I a la angiotensina II, una sustancia vasoconstrictora. La angiotensina II también estimula la secreción de aldosterona por la corteza suprarrenal. Los efectos beneficiosos del enalapril en la hipertensión y la insuficiencia cardíaca se deben a la supresión del sistema renina-angiotensina-aldosterona [46].

La inhibición de la ECA lleva consigo una disminución de los niveles plasmáticos de angiotensina II produciendo una disminución de la respuesta vasopresora y de la secreción de aldosterona. Aunque la disminución de la secreción de aldosterona no es muy grande, ocasiona un pequeño aumento de los niveles plasmáticos de potasio. En los pacientes hipertensos tratados con enalapril durante 48 semanas, este aumento llevó a ser de 0.2 mEq/L. En los pacientes tratados con enalapril asociado a un diurético tiazídico, no se observó practicamente ningún cambio en los niveles de potasio.

La supresión de la angiotensina II produce, por un efecto de retroalimentación negativa, un aumento de los niveles de renina. La ECA es similar a la kininasa, una enzima que degrada la bradikinina, y por lo tanto, la supresión de su actividad aumenta los niveles de bradikinina, un peptido con potentes efectos vasodepresores. No se sabe muy bien que papel juega este péptido en los efectos terapeúticos del enalapril. En efecto, si bien es seguro que el enalapril disminuye la presión arterial actuando sobre el sistema renina-angiotensina-aldosterona, no se sabe porque el fármaco reduce la hipertensión en pacientes con bajos niveles de renina.

1.3.5 Indicaciones del enalapril.

El enalapril está indicado en el tratamiento de todos los grados de hipertensión esencial y en la hipertensión renovascular. Puede emplearse como tratamiento inicial solo o concomitantemente con otros agentes antihipertensivos, especialmente diuréticos. El enalapril está indicado también en el tratamiento de la insuficiencia cardíaca congestiva.
El enalapril se debe administrar únicamente por vía oral. Dado que su absorción no se ve afectada por la comida, el enalapril puede administrarse antes, durante y después de las comidas. La dosis usual diaria varía desde 10 a 40 mg en todas las indicaciones. Se puede administrar el enalapril 1 ó 2 veces al día.

1.3.6 Efectos adversos.

El enalapril ha demostrado ser generalmente bien tolerado. En los estudios clínicos, la incidencia global de efectos indeseables no fue mayor con el enalapril que con placebo. Para la mayoría, los efectos indeseables han sido leves y transitorios. La suspensión del tratamiento fue requerida en el 6% de los pacientes.

Los efectos secundarios más comúnmente descritos fueron: sensación e inestabilidad y cefalea. En el 2-3 por 100 de las pacientes se describieron: fatigabilidad y astenia. Otros efectos secundarios con una incidencia menor del 2 por 100 fueron: hipotensión ortostática, síncope, náuseas, diarrea, calambres musculares y erupción cutánea. Se ha descrito tos seca y persistente con una frecuencia entre el 1 y el 2 por 100, y puede requerir la suspensión del tratamiento. Hipersensibilidad/edema angioneurótico: Se ha comunicado, raras veces, la aparición de edema angioneurótico de la cara, extremidades, párpados, lengua, glotis y/o laringe.

1.3.7 Hallazgos en las pruebas de laboratorio.

Raramente se observaron alteraciones en los parámetros estándar de laboratorio con la administración de enalapril que fuesen de significación clínica. Se han observado incrementos en la urea y creatinina séricas, reversibles con la suspensión de enalapril.

2. Antecedentes.

Se han descrito en estudios de otras patologías el uso del enalapril para disminución de la rigidez arterial. En el 2002, J.L Tycho Vuurmans. y J Am Soc Nephrol describieron la contribución de la sobrecarga de volumen y la angiotensina II del aumento de la velocidad de la onda de pulso de pacientes de hemodiálisis, en el cual se incluyeron 10 pacientes varones de edad promedio de 50 – 72 años con tratamiento con enalapril 5 mg cada 24hrs por 10 días y evaluación de VOPc (m/s) antes y después de la hemodiálisis (HD) sin y con tratamiento con enalapril, ellos encontraron que los pacientes obtuvieron una disminución significativa de la VOPc. Resultados similares de disminución de la VOP obtuvo A. Rehman en el mismo año, con uso de 80mg de valsartan por 3 días en 9 pacientes masculinos sanos de edad 24 años, antes de la medición.

Posteriormente Hideto Ishii (2008) obtuvo reducción de la VOP en 11 pacientes con el uso de candesartan (8mg) por 90 días en pacientes diabéticos tipo 2 e hipertensión, además en este estudio se encontró mayor respuesta con el uso de candesartán en comparación con el uso de bloqueadores de canales de calcio como amlodipino (6mg) y nifedipino (40mg). Recientemente Ludovit Paulis (abril 2016) realizó un estudio con olmesartán por 6 semanas (10mg / kg día) en 65 ratas wistar macho con hipertensión por medio de la inhibición de la oxido nitrico sintasa, el estudio reportó una reducción significativa de la VOP.

3. Justificación.

La artritis reumatoide (AR) es una enfermedad inflamatoria crónica sistémica considerada como un factor de riesgo cardiovascular independiente que está asociada a la carga inflamatoria acumulativa con la elevación de los niveles circulantes de citocinas inflamatorias (PCR, TNF-α e IL6). Estas citocinas pro-inflamatorias median el mecanismo de activación de las células endoteliales con el aumento de la enzima convertidora de angiotensina (ECA) y la generación de rigidez arterial.

El enalapril es un pro-fármaco que se hidroliza por esterasas hepáticas en enalaprilato, el cual es un inhibidor potente de la ECA. La inhibición de la ECA disminuye la resistencia vascular sistémica y las presiones arteriales media, diastólica y sistólica en varios estados hipertensivos, prescindiendo del mecanismo, los inhibidores de la ECA tienen una utilidad clínica amplia para enfermedades cardiovasculares.

Por lo anterior, consideramos que puede existir una asociación entre el uso de enalapril y la disminución de la rigidez arterial. Sin embargo, actualmente la información disponible en lo referente al uso de enalapril como profilaxis de cardiopatía isquémica en pacientes con AR es escasa con resultados poco contundentes. Por lo que el objetivo general del proyecto consiste en estudiar la asociación entre el uso del enalapril y la disminución de rigidez arterial.

Sometemos a consideración esta propuesta conformando un equipo de trabajo multidisciplinario con el objetivo de generar conocimiento sobre la relación del uso del enalapril y la disminución de rigidez arterial, que podría llevar al desarrollo de nuevas estrategias de terapéutica para la mejora en la calidad de vida de los pacientes con AR.

4. Planteamiento del problema.

La artritis reumatoide es una enfermedad inflamatoria crónica sistémica, es considerada como factor de riesgo cardiovascular con mayor tasa de mortalidad que la población general, debido a la mayor prevalencia de ateroesclerosis y aumento en la rigidez arterial.

La artritis reumatoide presenta un envejecimiento arterial prematuro manifestado como aumento de la rigidez arterial a causa del aumento de las citocinas pro-inflamatorias. La rigidez arterial es definida como la capacidad reducida de una arteria para expandirse o contraerse en respuesta a cambios de presión, y el eje renina – angiotensina – aldosterona es uno de los factores importantes en la rigidez arterial en otras enfermedades.

Estos datos sugieren que existe una asociación entre los mecanismos de daño vascular en AR y el sistema renina – angiotensina – aldosterona. Por lo que consideramos que el bloqueo del sistema renina-angiotensina – aldosterona con enalapril sería una estrategia eficaz para la disminución de la rigidez arterial en pacientes con artritis reumatoide.

4.1 Pregunta de Investigación.

¿Cuál es el efecto de la administración del enalapril en pacientes con artritis reumatoide en la rigidez arterial?

5. Hipótesis.

La administración del enalapril disminuye la rigidez arterial en pacientes con artritis reumatoide.

5.1 Hipótesis Alterna.

La administración del enalapril aumenta la rigidez arterial en pacientes con artritis reumatoide.

5.2 Hipótesis Nula.

La administración del enalapril no tienen ningún efecto en la rigidez arterial en los pacientes con artritis reumatoide.

6. Objetivos.

6.1 Objetivo general.

Evaluar el efecto del enalapril en la rigidez arterial en pacientes con artritis reumatoide.

6.2 Objetivos específicos.

- Determinar la velocidad de onda de pulso por medio del PulsePen y pOpmétre en pacientes con AR al inicio y 90 días después de iniciar el tratamiento con enalapril.
- Determinar el índice vascular corazón-tobillo por medio del CAVI en pacientes con AR al inicio y 90 días después de iniciar el tratamiento con enalapril.
- Determinar el índice carotideo por medio del USG carotideo en pacientes con AR al inicio y 90 días después de iniciar el tratamiento.
- Determinar los niveles de los anticuerpos anti-CCP ELISA y FR por inmunoturbidimetría antes y después de la intervención
- Evaluar la actividad de la enfermedad por el *Disease activity score on 28 joints* (DAS-28) basado en VSG y PCR antes y después de la intervención.
- Determinar los niveles séricos de lípidos en AR, colesterol, triglicéridos, LDL, VLDL, HDL y glucosa, antes y después de la intervención.
- Reportar la tolerabilidad de la administración de maleato de enalapril

7. Materiales y métodos.

7.1 Diseño del estudio.

Ensayo clínico doble ciego con asignación al azar en grupos paralelos.

7.2 Universo del estudio.

Pacientes voluntarios mujeres que acudan a la consulta externa de Reumatología del Hospital Civil "Juan I. Menchaca", con diagnóstico de artritis reumatoide de acuerdo a los criterios ACR/EULAR 2010, que no tengan historia de comorbilidad cardiovascular ni diagnóstico previo de DM tipo 2, enfermedad tiroidea, hepática o renal, que de manera voluntaria acepten participar en el estudio y firmen el consentimiento bajo información.

7.3 Lugar de trabajo.

El estudio se llevará a cabo en el Instituto de Investigación en Reumatología y del Sistema Musculo-esqueletico, Departamento de Biología Molecular, Centro Universitario de Ciencias de la Salud, Universidad de Guadalajara.Jalisco, México.

7.4 Intervención y seguimiento.

Se conformarán dos grupos de estudio de 32 pacientes cada uno que cumplan los criterios de inclusión y exclusión y que otorguen su consentimiento para participar en el proyecto de investigación. Los grupos serán conformados mediante asignación al azar por medio de una tabla de números aleatorios, se asignará al voluntario a uno de los siguientes grupos:

- Grupo maleato de enalaprilo (5 mg cada 12 hrs).
- Grupo placebo (cada 12 hrs).

7.4.1 Selección o asignación de los sujetos de estudio a los grupos de estudio.

Se incluirán de manera consecutiva a los pacientes con AR provenientes de la consulta externa del Servicio de Reumatología del Hospital Civil “Juan I. Menchaca” que cumplan los criterios de inclusión y exclusión y que otorguen su consentimiento para participar en el proyecto de investigación.

8. Tamaño de muestra.

El tamaño de la muestra fue calculado con la ecuación de respuesta continua para ensayos clínicos grupos paralelos:

$$n = 2 \cdot \left[\frac{\sigma}{\Delta}\right]^2 \cdot c$$

En donde se tomaron en cuenta los siguientes valores para la sustitución de la ecuación:

- La σ representa el valor de la desviación estándar de la variable de desenlace primaria, la que corresponde a 1.3
- La Δ se muestra la diferencia clínicamente relevante esperada tras la administración con maleato de enalapril en la rigidez arterial, la cual corresponde a 1 m/s.
- La c es una constante dependiente del nivel de confianza estadística en un 95% y poder estadístico del 80%. Siendo el valor de c equivalente a 7.9

Resultando un tamaño de muestra del 26.7 al cual se suma 5.34 equivalente al 20% por pérdidas resultando un total de 32.04 y considerando un total de 32 sujetos asumido por igual para los dos grupos.

9. Criterios de selección.

9.1 Criterios de inclusión.

- Pacientes clasificados con AR de acuerdo a los ACR/EULAR 2010.
- Edad (mayores de 18 años).
- Mujeres.
- Pacientes atendidos en la consulta externa del Servicio de Reumatología del Hospital Civil “Juan I. Menchaca”.
- Que acepten participar en el estudio y firmen carta de consentimiento bajo información.
- Mantener tratamiento reumatológico con fármacos modificadores de la enfermedad (DMARDS).

9.2 Criterios de no inclusión.

- Pacientes con diagnóstico previo de diabetes mellitus, hipertensión arterial sistémica, enfermedad tiroidea, renal o hepática.
- Antecedente de infarto agudo al miocardio, arritmias cardiacas, evento vascular cerebral o insuficiencia cardiaca.
- Pacientes fumadores activos en los últimos 6 meses.
- Pacientes con deseo de concepción próxima, embarazadas o en periodo de lactancia.
- Pacientes con tensión arterial < 90/60 mmHg.
- Pacientes que no acepten participar en el estudio.
- Pacientes con un IMC mayor a ≥40 kg/m^2
- Pulsos carotideo y femoral no palpables.

9.3 Criterios de eliminación.

- Pacientes que retiren su consentimiento bajo información para el estudio.

- Pacientes que padezcan efectos adversos graves al enalapril que condicionen el retiro del estudio.
- Sujetos que por causa médica o de salud salgan del estudio.
- Pacientes con un apego menor del 80% al tratamiento.
- Pacientes que durante el estudio presenten manifestaciones extrarticulares y/o que requieran modificación de tratamiento con DMARS.

10. Variables.

10.1 Independientes.

- Administración de maleato de enalaprilo.
- Administración de placebo.

10.2 Dependientes.

- Velocidad de onda de pulso carotido-femoral por PulsePen.
- Diferencia de velocidad de onda de pulso carotido-femoral por PulsePen.
- Progresión de velocidad de onda de pulso carotido-femoral por PulsePen.
- Velocidad de onda de pulso por pOpmetre.
- Diferencia de velocidad de onda de pulso por pOpmetre.
- Progresión de velocidad de onda de pulso porpOpmetre.
- Índice vascular corazón-tobillo por CAVI.
- Diferencia del índice vascular corazón-tobillo por CAVI.
- Progresión del índice vascular corazón-tobillo por CAVI.

10.3 Intervinientes.

- Nutrición (Dieta, Pesos, Talla, IMC).
- Edad.
- Actividad de la enfermedad (DAS28, VSG, PCR, FR, Anti-CCP).

10.4 Definición de las variables.

- **Edad:** Tiempo en años transcurrido a partir del nacimiento de un individuo.
- **Género:** Sexo biológico con el que se nace femenino o masculino.
- **DAS-28: Disease Activity Score.** Medición de la actividad de la enfermedad en la que se considera el número de articulaciones dolorosas, inflamadas, escala visual

análoga sobre la puntuación del enfermo velocidad de sedimentación globular y proteína C reactiva.

- **HAQ: HEALTH ASSESSMENT QUESTIONNAIRE.** Instrumento de evaluación de la discapacidad, predice el deterioro funcional, discapacidad laboral, mortalidad prematura.
- **Tiempo de evolución de la enfermedad:** Tiempo transcurrido del diagnóstico de la enfermedad a la captura de los datos.
- **VSG:** Prueba diagnóstica de laboratorio consiste en medir la velocidad con la que sedimentan los glóbulos rojos, es un reactante de fase aguda, es un marcador inespecífico, cuya elevación puede implicar procesos inflamatorios, infecciosos o neoplásicos.
- **Proteína C reactiva:** Es una proteína plasmática circulante, que aumenta sus niveles en respuesta a la inflamación (proteína de fase aguda).
- **Factor reumatoide:** Es un autoanticuerpo del tipo IgM producido contra la porción Fc de la inmunoglobulina G (Ig G). Los títulos se encuentran elevados en ciertas enfermedades reumáticas y en algunas infecciones crónicas.
- **Anti-CCP:** Anticuerpos contra péptidos citrulinados cíclicos obtenidos a partir de proteínas citrulinadas, altamente específicos para el diagnóstico de AR. Se considerarán como positivos si en la prueba de ELISA la absorbancia es mayor a 5 U/mL.
- **Peso:** El peso es una medida de la fuerza gravitatoria que actúa sobre un objeto. El peso equivale a la fuerza que ejerce un cuerpo sobre un punto de apoyo, originada por la acción del campo gravitatorio local sobre la masa del cuerpo.
- **Talla:** Estatura de un individuo expresada en centímetros .
- **Índice de masa corporal (IMC):** Es una medida de asociación entre la masa y la talla de un individuo, que se calcula con la formula, IMC: peso/talla al cuadrado.
- **Velocidad de Onda de Pulso central (VOPcf).** Es una medida de velocidad de la onda de pulso que genera el ventrículo izquierdo y se propaga al sistema cardiovascular, se determina a intervalos de dos, primero la colocación del tonómetro en la arteria carótida común y después el procedimiento en la arteria femoral, se calcula VOP = distancia entre dos segmentos arteriales / retardo de tiempo y sus unidades de medida son metros / segundo (m/s).
- **Velocidad de Onda de Pulso central (VOP / pOpmetre).** Es una medida de velocidad de la onda de pulso que genera el ventrículo izquierdo y se propaga al

sistema cardiovascular, se determina utilizando fotodiodos de infrarrojos en el dedo de la mano y el dedo del pie. La medición de la VOP por pOpmétre es en función de la talla del paciente y la segunda derivada de la diferencia del tiempo entre el pulso de cada dedo.

- **Índice Vascular Corazón-Tobillo.** Es una herramienta de medición oscilométrica, un método de exploración arteriosclerótica para calcular la función vascular normal independiente de la presión arterial, mide la tensión arterial en las cuatro extremidades. Se calcula mediante el parámetro de rigidez B obtenido a partir de la ecuación de Bramwell-Hill.

10.5 Operacionalización de variables.

Variables		Unidad de Medición	Escala	Descriptivos	Inferencial
**Independiente**	Maleato de enalaprilo	-	Cualitativa nominal	Proporción	X^2
	Placebo	-	Cualitativa nominal	Proporción	X^2
**Dependiente**	Velocidad de onda de pulso central (PulsePen)	m/s	Cuantitativa	Media /DE	t Student
	Diferencia de VOP (PulsePen)	m/s	Cuantitativa	Media /DE	t Student
	Progresión de VOP (PulsePen)	m/s	Cuantitativa	Media /DE	t Student
	VOP (pOpmetre)	m/s	Cuantitativa	Media /DE	t Student
	Diferencia VOP (pOpmetre)	m/s	Cuantitativa	Media /DE	t Student
	Progresión (pOpmetre)	m/s	Cuantitativa	Media /DE	t Student
	Indice Vascular (CAVI)	-	Cuantitativa	Media /DE	t Student
	Diferencia índice vascular (CAVI)	-	Cuantitativa	Media /DE	t Student
	Progresión índice vascular (CAVI)	-	Cuantitativa	Media /DE	t Student

**Interviniente**	Edad	Años	Cuantitativa	Media /DE	Wilcoxon y U de Mann Whithey
Actividad de la enfermedad	FR	mg/dL	Cuantitativa	Media /DE	t Student
	Anti-CCP	UI/mL	Cuantitativa	Media /DE	t Student
	DAS-28	-	Cuantitativa	Media /DE	t Student
	HAQ	-	Cuantitativa	Media / DE	t Student
	VSG	mm/h	Cuantitativa	Media /DE	t Student
	PCR	mg/dL	Cuantitativa	Media /DE	t Student
Nutrición	Peso	kg	Cuantitativa	Media /DE	t Student
	Talla	cm^2	Cuantitativa	Media /DE	t Student
	IMC	kg/m^2	Cuantitativa	Media /DE	t Student
	CC		Cuantitativa	Media /DE	t Student

11. Grupo de estudio.

Se conformarán dos grupos de 32 pacientes cada uno:

1. Placebo.
2. Maleato de enalaprilo.

Grupo maleato de enalaprilo a dosis 5 mg cada 12 hrs vía oral, cada una antes de cada alimento y grupo placebo una dosis cada 12 hrs vía oral, cada una antes de cada alimento, ambos grupos por 90 días.

11.1 Asignación del medicamento de la intervención y cegamiento.

La conformación de los grupos se llevará a cabo mediante asignación al azar con códigos obtenidos mediante un generador de números aleatorios a uno de los dos grupos, según el código doble ciego con números al azar. Ni el paciente, ni el investigador conocerán a que intervención farmacológica corresponde a cada grupo.

12. Determinaciones clínicas.

- **Edad:** Años cumplidos.
- **Género:** Se registrará M (Masculino) y F (Femenino).
- **Peso.** Se tomará el peso (kg) utilizando el sistema TANITA BC418® Tokio, JPN, con 0.01 kg de precisión).
- **Talla.** La talla (cm) con el estadímetro Seca 214 (GmbH & Co. KG. Hamburgo, Alemania con 1 mm de precisión). Para su medición se solicitará al participante descalzarse, mantener la posición de bidepestación con los talones juntos y una separación de alrededor de 5 cm entre sus primeros ortejos. En posición erguida, se alineó la comisura externa de uno de los ojos con el orificio del conducto auditivo externo ipsilateral.
- **Índice de masa corporal.** El índice de masa corporal (IMC) se calculará con la siguiente fórmula IMC = Peso (kg)/Talla^2 (cm) = kg/m^2.
- **Circunferencia de cintura:** Se medirá con una cinta Lufkin^{MR} debe ser realizada a nivel la línea media axilar, en el punto medio que se encuentra entre la parte inferior de la última costilla y la parte más alta de la cadera Se realiza con el paciente en posición de pie, y al final de una espiración normal sin ropa.
- **Presión arterial periférica.** La presión arterial periférica medida en consultorio, se determinará mediante el esfigmomanómetro oscilométrico automático digital OMRON® HEM 907XL Tokio Japan, con el paciente cómodamente sentado, con los pies bien apoyados en el piso y después de 5 min de reposo, se seleccionará el brazalete de acuerdo a la circunferencia braquial de 22-32 cms se utilizará el brazalete mediano, y de 32-42 cms se utilizará el brazalete grande, se ajusta 3 cm por arriba del pliegue del codo del brazo izquierdo para rodear perfectamente la circunferencia braquial. : presión arterial (siguiendo las recomendaciones internacionales(O'Brien, Asmar et al. 2005).
- **Frecuencia respiratoria.** Se medirá mediante la auscultación directa del área pulmonar y es definida como el número de ciclos espiratorios e inspiratorios completados en un periodo de 60 segundos.
- **Frecuencia cardiaca.** Se medirá mediante la auscultación directa del área cardiaca y es definida como el número de latidos cardiacos en un periodo de 60 segundos.

- **Temperatura.** Se medirá mediante termómetro de mercurio, en región axilar durante 3 min, la medición será en °C.
- **DAS 28** La actividad de la enfermedad será evaluada con el Disease activity index (DAS28). Primero, se hará el marcado por el enfermo con AR de la escala visual análoga (EVA) para la salud general en una línea con una longitud 0 a 100 mm, en donde 0 mm equivalen al mejor estado de saludo posible y 100 al peor; después se hará el conteo de 28 articulaciones y se registraran en el anexo 8, el número de articulaciones dolorosas (NAD) y tumefactas (NAT). Se calculará el DAS-28 con la fórmula: $DAS28=0.56(\sqrt{NAD})+0.28(\sqrt{NAT})+0.7(lnVSG)+0.14(EVA)$.
- **Cuestionario de antecendentes personales hereditarios, patologicos, no patológicos y gineco-obstetricos.** Se realizará por medio de un instrumento de evaluación el cual se efectuará por interrogatorio directo.
- **Velocidad de Onda de Pulso central (VOPcf).** Se medirá la velocidad de la onda de pulso que genera el ventrículo izquierdo y se propaga al sistema cardiovascular, se determina a intervalos de dos, primero la colocación del tonómetro en la arteria carótida común y después el procedimiento en la arteria femoral, se calcula VOP = distancia entre dos segmentos arteriales / retardo de tiempo y sus unidades de medida son metros / segundo (m/s).
- **Velocidad de Onda de Pulso central (VOP pOpmetre).** Se medirá la VOP que genera el ventrículo izquierdo y se propaga al sistema cardiovascular, se determina utilizando fotodiodos de infrarrojos en el dedo de la mano y el dedo del pie. La medición de la VOP por pOpmétre es en función de la talla del paciente y la segunda derivada de la diferencia del tiempo entre el pulso de cada dedo.
- **Índice Vascular Corazón-Tobillo.** Se mide la tensión arterial en las cuatro extremidades. Se calcula mediante el parámetro de rigidez B obtenido a partir de la ecuación de Bramwell-Hill.

DETERMINACIONES CLÍNICAS.		
Determinación	Equipo	Método
Edad	Interrogatorio	Años cumplidos
Peso	TANITA BC418® Tokio, JPN, con 0.01 kg de precisión.	Isak (Sociedad Internacional para el Avance de la Cineantropometría)
Talla	Estadímetro Seca 214 (GmbH & Co. KG. Hamburgo, Alemania con 1 mm de precisión).	
IMC	fórmula IMC = Peso (kg)/Talla^2 (cm) = kg/m^2	OMS (Organización Mundial de la Salud)
CC	Cinta métrica de fibra de vidrio	
PA	esfigmomanómetro	AHA (Asociación Americana del Corazón)
FR	Estetoscopio	número de ciclos espiratorios e inspiratorios completados (60s)
FC	Estetoscopio	número de latidos cardiacos (60s)
DAS-28	Instrumento. EVA (0-100mm) 28 articulaciones D y T	fórmula:DAS28=0.56(√NAD)+0.28(√NAT)+0.7(lnVSG)+0.14(EVA).
HAQ	Instrumento de evaluación de discapacidad	>0.5= afectación
VOP	Pulse Pen (DiaTecne s.r.l., Milán, Italia)	VOP = distancia entre dos segmentos arteriales / retardo de tiempo y sus unidades de medida son metros / segundo (m/s)
VOP	pOpmetre (Axelife sas 300)	VOP = la segunda derivada de la diferencia del tiempo entre el pulso de cada dedo. (m/s)
Índice Vascular	CAVI (VaSera 1000)	Se calcula mediante el parámetro de rigidez B obtenido a partir de la ecuación de Bramwell-Hill.

13 Determinación de laboratorio.

13.1 Obtención de muestras biológicas.

Por punción venosa en el brazo para obtener muestras de sangre en tubos del sistema vacutainer® (BD Diagnostic Systems Montenegro 1402, (C1427AND) Buenos Aires – Argentina), con anticoagulante EDTA-K3 1 mg/ml, tubo CPT para la VSG, y sin anticoagulante, este una vez que coagule a temperatura ambiente se centrifugara a 1509 RCF (Rotanta 460R, Andreas HettichGmbH& Co. KG.) por 10 min. Se separará el suero en alícuotas para ser almacenado a −80 °C hasta su análisis.

- Velocidad de sedimentación globular (VSG) mm/h, se medirá por el método de Westerngreen. Se cargará una pipeta de Westergreen –en posición de 90° con respecto a la superficie– con una muestra de sangre anticoagulada con citrato previamente homogeneizada y en el momento de llegar a la marca 0 mm se iniciará el conteo hasta 60 minutos, en este momento se leera la sedimentación eritrocitaria y se registrará en mm/hora.
- Proteína C reactiva de alta sensibilidad (hs-pCr, mg/L), por turbidimetria reacción antígeno-anticuerpo anti-proteína C reactiva humana, con límite de detección de 0.15 mg/L, y factor reumatoide con el sistema Selectra E, Randox laboratories.
- Se medirán la positividad de ACPA con un kit comercial de anticuerpos contra anti-CCP2 de Axis Shield.
- Se medirán la positividad de factor reumatoide con un kit comercial de anticuerpos.
- Para la determinación de la glucosa sérica se utilizará la técnica de glucosa oxidasa, expresada en mg/dl. Reactivo Merck México, S.A. La técnica glucosa oxidasa oxida a la glucosa y genera ácido glucónico y H_2O_2. El peróxido de hidrógeno liberado reacciona con un cromógeno (fenol/4-aminoantipirina) por la reacción de Trinder, para dar una quinona que absorbe entre 492 y 550 nm. La intensidad de color producida es directamente proporcional a la concentración de glucosa.
- El colesterol total (CT), C-HDL, C-LDL y triglicéridos (TG), se evaluarán por la técnica de colorimetría, en la que se utilizan reactivos de *Vitros*, Ortho-Clinical Diagnostics, Inc. a Johnson-Johnson Company, Rochester, NY, USA. La técnica

es la siguiente: el reactivo Vitros, es un elemento analítico seco, multicapas, incorporado en un soporte de poliéster en el que se deposita una gota de la muestra de suero del paciente, la cual es distribuida de manera uniforme por la capa difusora a las capas subyacentes. Posteriormente se da la reacción entre la muestra y el elemento a determinar para producir peróxido de hidrogeno. Finalmente, el peróxido de hidrógeno oxida un colorante en presencia de peroxidasa para generar un color. La densidad del colorante formado es proporcional a la concentración del metabolito presente en la muestra y es medida por espectrofotometría de reflexión.

**Determinaciones de Laboratorio**		
Determinación	Equipo	Método
VSG	pipeta de Westergreen (60 min)	método de Westerngreen
PCR	sistema Selectra E, Randox laboratorios	turbidimetria
FR	sistema Selectra E, Randox laboratorios	turbidimetria
Anti-CCP	kit comercial de anticuerpos contra anti-CCP de Axis Shield.	ELISA
Glucosa	Reactivo Merck México	técnica de glucosa oxidasa
CT, HDL, LDL, TG	Reactivos de *Vitros*, Ortho-Clinical Diagnostics, Inc. a Johnson-Johnson Company, Rochester, NY, USA	técnica de colorimetría

14. Análisis estadístico.

Para el análisis estadístico se considerarán dos grupos: 1) Grupo de tratamiento con enalapril 5 mg vía oral c-12 hrs y 2) Grupo control con placebo c-12 hrs. La distribución de las variables se evaluará con la prueba de Kolmogorov-Smirnov. De acuerdo a su distribución las variables continuas se reportarán como media ± desviación estándar o mediana (rango intercuartil). Las variables categóricas se reportarán como frecuencias. Las variables continuas se compararán utilizando la prueba de t de Student para variables dependientes o la prueba de U de Mann-Whitney dependiendo de su distribución. Las variables categóricas mediante la pruebas de X^2 o exacta de Fisher según corresponda. Un valor de $P < 0.05$ se considerará como estadísticamente significativo. Se realizará un análisis de ANCOVA con modo hacia atrás y una $P = 0.05$ para la entrada y $P = 0.10$ para la eliminación. Todos los datos se analizarán con el programa SPSS 24.0 (SPSS Inc. Chicago, IL) y GraphPad Prism versión 6.00 para Windows (GraphPad Sofware, La Jolla, CA), considerando que un nivel de P <0.05 de dos colas es estadísticamente significativo para el análisis.

15. Descripción del estudio.

15.1 Desarrollo de la fase de campo.

Visita 0, Escrutinio (Día -1).

Todos los individuos posibles candidatos serán citados a las 08:00 am con 12 horas de ayuno. En esta primera visita se proporcionará información referente al estudio; los objetivos, características, procedimientos, así como también sus potenciales beneficios y riesgos. Si el individuo acepta y el encargado de la investigación decide que el paciente cumple los criterios para la participación en el estudio, se procederá a la firma del consentimiento bajo información. Se realizará la historia clínica al individuo, se tomarán medidas antropométricas (peso, talla, IMC, porcentaje de grasa corporal, circunferencia de cintura) signos vitales (presión arterial periférica, frecuencia cardiaca y frecuencia respiratoria), evaluación del estado de la enfermedad (DAS28, HAQ). Se procederá a la toma de muestra sanguínea (10 ml de sangre) para medir glucosa de ayuno, perfil de lípidos, PCR, VSG, anticuerpos (FR y anti-CCP).

Visita 1. Basal: Revisión de criterios.

Se valoran los resultados de laboratorio y se corrobora que el individuo cumpla con todos los criterios de inclusión y de ser así se procederá a la asignación al azar de su tratamiento, el paciente podrá entrar en el grupo estudiado con enalapril o placebo (el tratamiento de enalapril consistirá en 5 mg cada 12 horas antes de cada alimento, al igual que el grupo placebo). Se procederá a la medición de la velocidad de onda de pulso e índice vascular, y se otorgará un diario al paciente donde se describe la posología diaria, además se entregara el medicamento que corresponde al primer mes de la intervención, y finalmente se darán recomendaciones generales de nutrición ya que durante el estudio no está indicado iniciar algún tipo de dieta para bajar de peso o un incremento en la actividad física. También se incluirán el teléfono de los encargados para cualquier duda que el paciente presente referente al proyecto. Se programará cita al paciente de control a los 30 días posteriores de haber iniciado el tratamiento.

Visita 2 y 3. Seguimiento (Días 30, 60 ± 3 respectivamente).

Durante el primero y segundo mes de la intervención se le citará para vigilar apego y tolerabilidad al tratamiento mediante cuestionarios, se tomarán medidas antropométricas, signos vitales y se proporcionará el medicamento correspondiente al mes en curso.

Visita 4. Fin del periodo de intervención (Día 90± 3).

Posteriores a los 90 días al inicio de la intervención, se citará nuevamente al individuo y se tomarán las mediciones clínicas y de laboratorio del mismo modo que en la visita basal, con ayuno de 12 horas, Se evaluarán el apego y tolerabilidad al tratamiento.

Visita 5: Evaluación y entrega de resultados (Día 120± 3).

Después de 4 semanas se citará al paciente para entrega de resultados que incluyen: escrutinio, basales y finales a la intervención y se le explicará la evolución de cada una de ellas. Finalmente se le hará un resumen clínico que incluye recomendaciones médico-nutrimentales, así como su constante vigilancia médica en caso necesario.

15.2 Adherencia terapéutica.

La adherencia al tratamiento será evaluada mediante métodos indirectos, cuestionarios contestados por el mismo paciente. La tasa de adherencia al tratamiento se reporta en porcentaje de forma individual. Aunque no existe un consenso que defina el punto de corte para determinar una adherencia adecuada, se tomará en cuenta como adherencia terapéutica aceptable a más del 80% de las tomas prescritas del fármaco de investigación.

15.3 Criterios de seguridad.

Todos los pacientes serán vigilados ante la posible presencia de eventos adversos de cualquier tipo a lo largo del estudio. Se vigilará la presencia de eventos adversos. Desde el inicio del estudio los pacientes contarán con los números telefónicos de emergencia de los investigadores responsables. Durante todo el periodo de seguimiento existe la posibilidad de realizarse determinaciones de laboratorio en caso de sospecharse eventos adversos o efectos tóxicos del medicamento. Se considerará como pérdida y abandono del seguimiento cuando el paciente extravíe el fármaco que le fue proporcionado y no se informe a tiempo al responsable o tenga una adherencia menor a 80%.

15.4 Recolección de datos.

Se realizará por medio de historia clínica, posteriormente se realizará una base de datos para dar seguimiento al progreso del paciente, se vigilará en todo momento el adecuado manejo y discreción de los datos del paciente, además se integrará una base de datos electrónica.

15.5 Difusión de los resultados finales de la investigación.

Los resultados se entregarán de manera confidencial a cada uno de los participantes, así como medidas especiales que ameriten a su autocuidado o seguimiento acorde a los resultados. Los resultados del estudio serán publicados en revista científica indexada, presentación en eventos como congresos y conferencias afines a la investigación.

16. Aspectos éticos.

El estudio cumple con las normas internacionales para realizar investigación en seres humanos, de acuerdo a la Ley General de Salud en materia de principios éticos de investigación en seres humanos Artículo 17 sección 3 se considera como una investigación con un riesgo mayor que al mínimo y en conformidad con los principios éticos para investigación farmacológica en seres humanos detallados en la última revisión de la declaración de Helsinki y de la Conferencia Internacional de Armonización.

La naturaleza, propósitos y riesgos potenciales del estudio serán explicados a cada participante. Todos los pacientes darán su consentimiento bajo información con la firma de testigos y su relación con ellos. Conforme a los lineamientos de las buenas prácticas clínicas todos los participantes en el estudio serán identificados únicamente mediante iniciales y número en la base de datos. Los expedientes y resultados de laboratorio se encontrarán disponibles solo para los investigadores principales y con las restricciones de ley para el participante. Los pacientes voluntarios tienen el derecho de retirarse cuando ellos lo deseen, con el previo aviso a su médico investigador sin afectar su relación médico- paciente ni afectar su tratamiento concomitante. También se procederá en base a la NOM-012-SSA-2012 que establece los criterios para la ejecución de proyectos de investigación para la salud. NOM 007-SSA3-2011 para la organización y funcionamiento de laboratorios clínicos. En cuestiones de bioseguridad se procederá de acuerdo a la NOM-087-ECOL-SSA1-2002, que establece los requisitos para la separación, envasado, almacenamiento, recolección, transporte, tratamiento y disposición final de los residuos peligrosos biológicos-infecciosos que se generan en establecimientos que presten servicios de atención médica. Los resíduos peligrosos biológicos-infecciosos (RPBI) a utilizar serán la sangre, definida como el tejido hemático con todos sus elementos, los resíduos no anatómicos, objetos punzocortantes, el manejo de RPBI cumplirá con las disposiciones correspondientes como identificación de los resíduos, envasado, almacenamiento temporal, recolección y transporte especificadas en la misma NOM-087.

El manejo de reactivos químicos será acorde a la NOM-018-STPS-2000 sistema para la identificación y comunicación de peligros y riesgos por sustâncias químicas peligrosas en los centros de trabajo. El grado de riesgo del uso de los reactivos químicos

son acordes a riesgo igual a 0 o 1, se cumplirán las especificaciones como identificación, señalización, equipo de protección personal, y manejo adecuado estipulados en la NOM-018.

17. Financiamiento.

El presente estudio no cuenta actualmente con patrocinio propio, su financiamiento proviene del Programa de Incorporación y Permanencia de Posgrado en el Programa Nacional de Posgrados de calidad (PROINPEP), de igual forma se obtiene financiamiento paralelo a otros estudios suficientes para cubrir el costo estimado de este protocolo la cual asciende a $90,089.80 MNX.

18. Recursos humanos.

18.1 Equipo de Trabajo.

Grupo de Investigación en rigidez arterial en artritis reumatoide
Centro Universitario de Ciencias de la Salud, C. U. C. S. Universidad de Guadalajara.

Para el desarrollo del proyecto se cuenta con los recursos humanos necesarios para llevar a cabo los análisis de laboratorio y evaluación de los pacientes en el Instituto de Investigación en Reumatología y del Sistema Musculo-esquelético (IIRSME), así también en el Instituto de Terapéutica Experimental y Clínica (INTEC). De forma breve se describe la función principal de cada uno de ellos:

Dra. en C. Mónica Vázquez del Mercado Espinosa. - Investigador Responsable. Directora del Instituto de Investigación en Reumatología y Sistema Músculo Esquelético (IIRSME). Reumatóloga y doctora en ciencias en biología molecular.

Dra. en C. Rosa Elena Navarro Hernández. - Investigador. Encargada de laboratorio del Instituto de Investigación en Reumatología y Sistema Músculo Esquelético (IIRSME). Químico fármaco-biólogo y doctora en ciencias biomédicas con orientación en inmunología.

Dr. en C. José de Jesús Eduardo Gómez Bañuelos. - Investigador. Instituto de Investigación en Reumatología y Sistema Músculo esquelético (IIRSME). Reumatólogo y doctor en Ciencias Biomédicas, encargado de la evaluación clínica-reumatológica de los pacientes.

Lic. MCP Felipe de Jesús Pérez Vázquez. – Estudiante del Doctorado en Farmacología del Instituto de Investigación en Reumatología y Sistema Músculo esquelético (IIRSME), Medico encargado de las citas de los pacientes, la evaluación clínica, el seguimiento de los pacientes durante el estudio, captura de los datos y entrega de los resultados.

Lic. QFB Gustavo Ignacio Díaz Rubio. - Doctorante. Doctorado en Biología Molecular en Medicina del Instituto de Investigación en Reumatología y Sistema Músculo esquelético (IIRSME). Químico fármaco-biólogo encargado de la toma de muestras, procesamiento de las mismas y evaluación de los resultados.

Dr. en C. Efraín Chavarría Ávila. - Investigador. Instituto de Investigación en Reumatología y Sistema Músculo Esquelético (IIRSME). Químico fármaco-biólogo y Doctor en ciencias biomédicas con orientación en inmunología, diplomado en bioestadística, encargado de la evaluación estadística de los datos, análisis estadístico y elaboración de tablas y gráficos.

Dr. en C. Ernesto German Cardona Muñoz. - Investigador. Director del Instituto de Terapéutica Experimental y Clínica (INTEC). Cardiólogo y Doctor en Farmacología.

Dr. David Cardona Müller. - Investigador. Instituto de Terapéutica Experimental y Clínica (INTEC). Cardiólogo, encargado de la evaluación cardiológica de los pacientes, así como la realización del ultrasonido carotideo.

Dr. en C. Fernando Grover Páez. - Investigador. Instituto de Terapéutica Experimental y Clínica (INTEC). Médico Internista, Doctor en Farmacología, encargado de la medición del índice corazón-tobillo (CAVI) y evaluación de los pacientes por sospecha de efectos adversos al medicamento.

Dr. en C. Carlos Gerardo Ramos Becerrra. - Investigador. Instituto de Terapéutica Experimental y Clínica (INTEC). Doctor en Hipertensión, encargado de la medición de la velocidad de onda de pulso por medio de pOpmetre y pulsepen.

Lic. en Nutrición Lesli Yazmín Lozano Padilla. - Nutrióloga del Instituto de Investigación en Reumatología y Sistema Músculo esquelético (IIRSME). Encargada de la evaluación de nutrición y medidas antropométricas de los pacientes.

Mrs. Cynthia Alejandra Gómez Ríos. - Asistente del Instituto de Investigación en Reumatología y Sistema Músculo esquelético (IIRSME). Encargada de la administración del instituto y recepción de los pacientes.

19. Recursos materiales.

Para el desarrollo del proyecto se cuenta con los recursos materiales necesarios para llevar a cabo los análisis de laboratorio y evaluación de los pacientes en el Instituto de Investigación en Reumatología y del Sistema Musculo-esquelético de forma breve se cuenta con centrífugas para separación de las muestras, ultra-congeladores para la conservación de las mismas. Espectrofotómetro para lectura de las placas de ELISA y equipo para el análisis de las imágenes obtenidas por quimioluminiscencia. Se cuenta con el PulsePen, pOpmétre y CAVI para el análisis cardiovascular en el Instituto de Terapéutica Experimental y Clínica (INTEC).

20. Conflictos de interés.

El presente estudio no presenta conflictos de interés de ninguna índole, tales como convenios con la industria farmacéutica o propia del investigador principal o al personal involucrado al estudio.

21. Referencias bibliográficas.

1. Gossec, L., M. Dougados, and W. Dixon, *Patient-reported outcomes as end points in clinical trials in rheumatoid arthritis.* RMD Open, 2015. **1**(1): p. e000019.
2. Burmester, G.R., E. Feist, and T. Dorner, *Emerging cell and cytokine targets in rheumatoid arthritis.* Nat Rev Rheumatol, 2014. **10**(2): p. 77-88.
3. Gibofsky, A., *Overview of epidemiology, pathophysiology, and diagnosis of rheumatoid arthritis.* Am J Manag Care, 2012. **18**(13 Suppl): p. S295-302.
4. Silman, A.J. and J.E. Pearson, *Epidemiology and genetics of rheumatoid arthritis.* Arthritis Res, 2002. **4 Suppl 3**: p. S265-72.
5. Tobon, G.J., P. Youinou, and A. Saraux, *The environment, geo-epidemiology, and autoimmune disease: Rheumatoid arthritis.* Autoimmun Rev, 2010. **9**(5): p. A288-92.
6. Cardiel, M.H. and J. Rojas-Serrano, *Community based study to estimate prevalence, burden of illness and help seeking behavior in rheumatic diseases in Mexico City. A COPCORD study.* Clin Exp Rheumatol, 2002. **20**(5): p. 617-24.
7. Bennett, K., et al., *Community screening for rheumatic disorder: cross cultural adaptation and screening characteristics of the COPCORD Core Questionnaire in Brazil, Chile, and Mexico. The PANLAR-COPCORD Working Group. Pan American League of Associations for Rheumatology. Community Oriented Programme for the Control of Rheumatic Disease.* J Rheumatol, 1997. **24**(1): p. 160-8.
8. Pelaez-Ballestas, I., et al., *Epidemiology of the rheumatic diseases in Mexico. A study of 5 regions based on the COPCORD methodology.* J Rheumatol Suppl, 2011. **86**: p. 3-8.
9. Karlson, E.W. and K. Deane, *Environmental and gene-environment interactions and risk of rheumatoid arthritis.* Rheum Dis Clin North Am, 2012. **38**(2): p. 405-26.
10. Too, C.L., et al., *Smoking interacts with HLA-DRB1 shared epitope in the development of anti-citrullinated protein antibody-positive rheumatoid arthritis: results from the Malaysian Epidemiological Investigation of Rheumatoid Arthritis (MyEIRA).* Arthritis Res Ther, 2012. **14**(2): p. R89.
11. Bax, M., et al., *Genetics of rheumatoid arthritis: what have we learned?* Immunogenetics, 2011. **63**(8): p. 459-66.

12. Feitsma, A.L., et al., *Identification of citrullinated vimentin peptides as T cell epitopes in HLA-DR4-positive patients with rheumatoid arthritis.* Arthritis Rheum, 2010. **62**(1): p. 117-25.
13. Padyukov, L., et al., *A gene-environment interaction between smoking and shared epitope genes in HLA-DR provides a high risk of seropositive rheumatoid arthritis.* Arthritis Rheum, 2004. **50**(10): p. 3085-92.
14. Lundstrom, E., et al., *Gene-environment interaction between the DRB1 shared epitope and smoking in the risk of anti-citrullinated protein antibody-positive rheumatoid arthritis: all alleles are important.* Arthritis Rheum, 2009. **60**(6): p. 1597-603.
15. Makrygiannakis, D., et al., *Smoking increases peptidylarginine deiminase 2 enzyme expression in human lungs and increases citrullination in BAL cells.* Ann Rheum Dis, 2008. **67**(10): p. 1488-92.
16. Pikwer, M., et al., *Early menopause and severity of rheumatoid arthritis in women older than 45 years.* Arthritis Res Ther, 2012. **14**(4): p. R190.
17. Brzustewicz, E. and E. Bryl, *The role of cytokines in the pathogenesis of rheumatoid arthritis--Practical and potential application of cytokines as biomarkers and targets of personalized therapy.* Cytokine, 2015. **76**(2): p. 527-536.
18. van Boekel, M.A., et al., *Autoantibody systems in rheumatoid arthritis: specificity, sensitivity and diagnostic value.* Arthritis Res, 2002. **4**(2): p. 87-93.
19. Hensen, S.M. and G.J. Pruijn, *Methods for the detection of peptidylarginine deiminase (PAD) activity and protein citrullination.* Mol Cell Proteomics, 2014. **13**(2): p. 388-96.
20. Mouterde, G., et al., *Association of anticyclic citrullinated peptide antibodies and/or rheumatoid factor status and clinical presentation in early arthritis: results from the ESPOIR cohort.* J Rheumatol, 2014. **41**(8): p. 1614-22.
21. Gerli, R., et al., *Association of anti-cyclic citrullinated peptide antibodies with subclinical atherosclerosis in patients with rheumatoid arthritis.* Ann Rheum Dis, 2008. **67**(5): p. 724-5.
22. Luban, S. and Z.G. Li, *Citrullinated peptide and its relevance to rheumatoid arthritis: an update.* Int J Rheum Dis, 2010. **13**(4): p. 284-7.
23. McInnes, I.B. and G. Schett, *Cytokines in the pathogenesis of rheumatoid arthritis.* Nat Rev Immunol, 2007. **7**(6): p. 429-42.

24. van der Helm-van Mil, A.H., et al., *A prediction rule for disease outcome in patients with recent-onset undifferentiated arthritis: how to guide individual treatment decisions.* Arthritis Rheum, 2007. **56**(2): p. 433-40.

25. Huizinga, T.W. and A. van der Helm-van Mil, *A quantitative approach to early rheumatoid arthritis.* Bull NYU Hosp Jt Dis, 2011. **69**(2): p. 116-21.

26. Gkaliagkousi, E., et al., *Cardiovascular risk in rheumatoid arthritis: pathogenesis, diagnosis, and management.* J Clin Rheumatol, 2012. **18**(8): p. 422-30.

27. Solomon, D.H., et al., *Cardiovascular morbidity and mortality in women diagnosed with rheumatoid arthritis.* Circulation, 2003. **107**(9): p. 1303-7.

28. Kramer, H.R. and J.T. Giles, *Cardiovascular disease risk in rheumatoid arthritis: progress, debate, and opportunity.* Arthritis Care Res (Hoboken), 2011. **63**(4): p. 484-99.

29. Crowson, C.S., et al., *How much of the increased incidence of heart failure in rheumatoid arthritis is attributable to traditional cardiovascular risk factors and ischemic heart disease?* Arthritis Rheum, 2005. **52**(10): p. 3039-44.

30. Kremers, H.M., et al., *High ten-year risk of cardiovascular disease in newly diagnosed rheumatoid arthritis patients: a population-based cohort study.* Arthritis Rheum, 2008. **58**(8): p. 2268-74.

31. Crilly, M.A., et al., *Arterial stiffness and cumulative inflammatory burden in rheumatoid arthritis: a dose-response relationship independent of established cardiovascular risk factors.* Rheumatology (Oxford), 2009. **48**(12): p. 1606-12.

32. Klocke, R., et al., *Arterial stiffness and central blood pressure, as determined by pulse wave analysis, in rheumatoid arthritis.* Ann Rheum Dis, 2003. **62**(5): p. 414-8.

33. Park, S. and E.G. Lakatta, *Role of inflammation in the pathogenesis of arterial stiffness.* Yonsei Med J, 2012. **53**(2): p. 258-61.

34. Holmes, M.V., et al., *Paradoxical association of C-reactive protein with endothelial function in rheumatoid arthritis.* PLoS One, 2010. **5**(4): p. e10242.

35. Roman, M.J., et al., *Arterial stiffness in chronic inflammatory diseases.* Hypertension, 2005. **46**(1): p. 194-9.

36. Vazquez-Del Mercado, M., et al., *Serum levels of anticyclic citrullinated peptide antibodies, interleukin-6, tumor necrosis factor-alpha, and C-reactive protein are associated with increased carotid intima-media thickness: a cross-sectional analysis of a cohort of rheumatoid arthritis patients without cardiovascular risk factors.* Biomed Res Int, 2015. **2015**: p. 342649.

37. Vassilopoulos, D., et al., *Adalimumab decreases aortic stiffness independently of its effect in disease activity in patients with rheumatoid arthritis.* Clin Rheumatol, 2015. **34**(2): p. 359-64.
38. Angel, K., et al., *Tumor necrosis factor-alpha antagonists improve aortic stiffness in patients with inflammatory arthropathies: a controlled study.* Hypertension, 2010. **55**(2): p. 333-8.
39. Wallberg-Jonsson, S., et al., *Increased arterial stiffness and indication of endothelial dysfunction in long-standing rheumatoid arthritis.* Scand J Rheumatol, 2008. **37**(1): p. 1-5.
40. Wong, M., et al., *Reduced arterial elasticity in rheumatoid arthritis and the relationship to vascular disease risk factors and inflammation.* Arthritis Rheum, 2003. **48**(1): p. 81-9.
41. Xi, X., et al., *A review on bradykinin-related peptides isolated from amphibian skin secretion.* Toxins (Basel), 2015. **7**(3): p. 951-70.
42. Wilkinson, I.B., et al., *Reproducibility of pulse wave velocity and augmentation index measured by pulse wave analysis.* J Hypertens, 1998. **16**(12 Pt 2): p. 2079-84.
43. Laurent, S., et al., *Expert consensus document on arterial stiffness: methodological issues and clinical applications.* Eur Heart J, 2006. **27**(21): p. 2588-605.
44. Yildiz, M., *Arterial distensibility in chronic inflammatory rheumatic disorders.* Open Cardiovasc Med J, 2010. **4**: p. 83-8.
45. Townsend, R.R., et al., *Recommendations for Improving and Standardizing Vascular Research on Arterial Stiffness: A Scientific Statement From the American Heart Association.* Hypertension, 2015. **66**(3): p. 698-722.
46. Igic, R. and R. Skrbic, *The renin-angiotensin system and its blockers.* Srp Arh Celok Lek, 2014. **142**(11-12): p. 756-63.

22. ANEXOS.

22.1 ANEXO 1. CARTA DE CONSENTIMIENTO BAJO INFORMACIÓN

“Efecto del uso de enalapril en la rigidez arterial en pacientes con artritis reumatoide”.

No firme este consentimiento bajo información si usted no está de acuerdo en participar en el estudio. El documento puede tener información o términos que no comprenda, por favor pida ayuda al médico investigador encargado o en su caso al médico designado para su entrevista que le resuelva todas sus dudas.

La artritis reumatoide es una enfermedad que afecta a las articulaciones generando dolor y deformidad articular. La artritis reumatoide además de afectar a las articulaciones daña a los vasos sanguíneos causando rigidez arterial y comprometiendo frecuentemente al corazón.

El maleato de enalaprilo, conocido comúnmente como enalapril es un medicamento que ha demostrado tener efectos benéficos para el tratamiento de la rigidez arterial en otras enfermedades. El enalapril es un medicamento que se elimina por riñon, y en general es bien tolerado, dentro de los efectos adversos se han reportado: cansancio, debilidad, presión baja, desmayos, náuseas, dolor de cabeza, diarrea, calambres musculares, alergia, tos seca, hinchazón de los parpados y lengua; los cuales se han presentado en baja frecuencia y en 94% de los casos no amerita suspensión del medicamento.

El almidón de maíz es un producto alimenticio que es utilizado en el área de investigación bajo el nombre de “placebo” el cual es considerado como una sustancia la cual no posee ninguna propiedad benéfica ni maléfica en el organismo.

El estudio será con cegamiento, es decir ni el investigador a cargo, ni usted, sabrán quien está tomando enalapril o placebo, a menos de que se requiera o hasta el final del estudio. Ambos serán proporcionados de forma gratuita, con la cantidad suficiente y con indicaciones de cómo debe de tomarla, además en todo momento usted podrá comunicar dudas o molestias que el tratamiento ocasione.

Objetivo del estudio.

El objetivo del estudio es evaluar y monitorizar sus niveles de velocidad de onda pulso, índice vascular y otros estudios de laboratorio tras la administración del enalapril o placebo.

Descripción del estudio.

En este estudio participan 64 pacientes; 32 pacientes por grupo.- Grupo de enalapril y Grupo de placebo. Si usted desea participar se abrirá un expediente y se realizara la historia clínica, se tomaran las pruebas de laboratorio y clínicas como peso, talla, circunferencia de cintura y signos vitales. Este estudio tiene una duración de 3 meses, con 6 visitas las cuales se escriben a continuación:

-Visita 1 (Escrutinio): El candidato será citado a las 08:00 am con 12 horas de ayuno. En esta primera visita se proporciona información referente al estudio; los objetivos, características, procedimientos, así como también sus beneficios y riesgos. Si usted acepta y el encargado del estudio decide que cumple los criterios de selección para la participación en el, se procederá a la firma de este consentimiento bajo información. Se realizará la historia clínica, revisión médica reumatológica, se tomaran medidas y signos vitales (peso, talla, IMC, porcentaje de grasa corporal, circunferencia de cintura, presión arterial, frecuencia cardiaca y frecuencia respiratoria), toma de muestra sanguínea (10 ml de sangre / equivale a 2 cucharadas soperas) para medir glucosa, colesterol, trigliceridos, velocidad de sedimentación globular, proteína C reactiva, niveles de factor de necrosis tumoral alfa (TNFa), interleucina-6 (IL-6) y de anticuerpos contra factor reumatoide (FR) y anticuerpos anti-citrulinados (anti-CCP). Por su seguridad e higiene, todo el material empleado en este estudio es estéril y desechable y al término de los análisis planeados, el resto de la muestra será destruida. El tiempo aproximado es de 30 minutos.

-Visita 2 (Basal): Se valoran sus resultados de laboratorio y se corrobora que cumpla con todos los criterios de inclusión, de ser así se realizarán los estudios cardiovasculares que consistirán en la medición de la velocidad de onda de pulso e índice vascular, y se procederá con la asignación al azar de su tratamiento, ya sea enalapril o placebo (el tratamiento será administrado una capsula de 5mg, 30 minutos antes de cada comida, dos veces al día c/12 hrs por 90 dias). Se entregará un diario donde se describe la dosis

diaria y además se entregará el medicamento que corresponde al primer mes de la intervención. Finalmente se darán recomendaciones generales de nutrición ya que durante el estudio no está indicado iniciar algún tipo de dieta para bajar de peso o un incremento en la actividad física. Se citará a los 30 días posteriores de haber iniciado el tratamiento, fecha que será registrada en su diario.

-Visita 3 y 4 (Seguimiento): Día 30 y 60 se citará al paciente para vigilar la cantidad de medicamento ingerido y la tolerabilidad al tratamiento, y se proporcionará el medicamento correspondiente al mes en curso.

-Visita 5 (Fin del periodo de intervención): Posteriores a los 90 días al inicio de la intervención, se le citará nuevamente y se tomarán las mediciones clínicas y de laboratorio de igual forma como se le realizaron en la primera visita.

-Visita 6 (Evaluación y entrega de resultados): A lo largo de 4 semanas se citará para entregar y explicar los resultados, incluyendo los iniciales y finales, también se le explicará la evolución de cada una de ellas. Finalmente, se le hará un resumen clínico con recomendaciones médico-nutrimentales, así como su constante vigilancia médica en caso necesario.

Resultados durante y al final del estudio e información nueva.

Durante el transcurso de este estudio, le informaremos cualquier hallazgo nuevo que sea importante para decidir si continua o abandona la intervención. Si le llegamos a proporcionar nueva información, le solicitaremos su consentimiento nuevamente para seguir participando en este estudio en caso de nuevas intervenciones.

Riesgos generales de la intervención.

Durante la recolección de las muestras de sangre se requerirá de la realización de una o más punciones (toma de sangre con aguja y jeringa) en alguna de las venas de sus brazos, durante la cual puede sentir dolor y ocasionalmente puede llegar a formarse un moretón en el área donde se realice la toma de sangre. Con el fin de evitar riesgos innecesarios para usted y su familia el medicamento que le sea dado o prescrito de forma estricta deberá ser consumido únicamente por usted y en la forma en que le sea indicado. Usted podría llegar a presentar: cansancio, debilidad, presión baja, desmayos, náuseas, dolor de cabeza, diarrea, calambres musculares, alergia, tos seca, hinchazón de los

parpados y lengua. Es importante mencionar que estos pueden o no presentarse, debido a las características físicas y genéticas de cada individuo. Es indispensable que usted informe al investigador tratante en esta investigación de todas las molestias que pudiera padecer a lo largo del estudio. El investigador conoce todas las molestias que puede llegar a presentar y la mejor forma de tratarlos, lo cual puede incluir la suspensión del tratamiento o disminución de la dosis del mismo, y en base al ARTÍCULO 14 DEL REGLAMENTO DE LA LEY GENERAL DE SALUD EN MATERIA DE INVESTIGACIÓN, la atención a todos los participantes de esta investigación será las 24 horas del día y en caso de presentarse cualquier adversidad o enmienda durante la ejecución del proyecto, el investigador lo comunicará al Comité de Ética en Investigación del Hospital Civil Juan I. Menchaca. Se cuenta con la disponibilidad de tratamiento médico y la indemnización a que legalmente se tenga por derecho por parte de la institución en el caso de daños que la ameriten directamente causados por la investigación, de igual forma si existen gastos adicionales, éstos serán absorbidos por el presupuesto de la investigación.

Beneficios de la intervención.

Usted no recibirá pago por su participación en este estudio, ni este estudio implica gasto para usted. Usted se beneficiará con una valoración que incluye: el peso, talla, índice de masa corporal, porcentaje de grasa corporal, presión arterial, circunferencia de cintura, determinaciones de laboratorio, además de recibir orientación profesional acerca de cuáles son las medidas que debe tomar para disminuir el riesgo de presentar complicaciones. Al finalizar el estudio se le dará seguimiento nutricional y apoyo multidisciplinario para tratar la artritis reumatoide en caso de ser necesario. Las visitas al hospital, los procedimientos ocasionados por el medicamento no tendrán ningún costo para usted de ser necesarios durante la intervención. Además, los resultados del presente estudio contribuirán al avance del conocimiento científico para nuevas aplicaciones en el uso de enalapril, así como también para el manejo de personas que como usted presentan el mismo problema.

Participación o retiro en el estudio.

Su participación en nuestro estudio es completamente voluntaria. Usted puede decidir no ingresar o descontinuar su participación en cualquier momento durante el estudio sin penalización o pérdida de beneficios. El investigador también podrá suspender su

participación sin su consentimiento si necesitara tratamiento adicional, no siguiera las instrucciones o hubiera sospecha de que el medicamento es nocivo para su salud. Sin embargo, aun contará con el beneficio de la atención médica y así como un seguimiento nutricional.

Privacidad y confidencialidad.

La información que se nos proporcione (como su nombre, teléfono y dirección), igual que sus respuestas a los cuestionarios y los resultados de sus pruebas clínicas y de laboratorio será guardada de manera confidencial para garantizar su privacidad. Nadie más tendrá acceso a la información que se nos proporcione durante su participación, al menos que usted así lo desee y/o que fuese necesario para proteger su bienestar y sus derechos.

Difusión de los resultados.

Los resultados se entregarán de manera confidencial a cada uno de los participantes asi como medidas especiales que ameriten a su autocuidado o seguimiento acorde a los resultados. El resultado del estudio será publicado en revista científica indexada y se proporcionará a cada uno de los participantes del estudio.

Personal de contacto para dudas y aclaraciones.

Si durante el estudio, usted llegara a tener preguntas o dudas que tengan que ver en este estudio de investigación puede comunicarse las 24 horas del día con el investigador principal del estudio.

A QUIEN CONSULTAR.

Para cualquier duda o aclaración relacionada con el trato, manejo del paciente, así como de la muestra para conocer una información específica o particular relacionada a la misma puede contactar al investigador principal:

Nombre: Dra. Mónica Vázquez del Mercado Espinosa
Correo electrónico: dravme@hotmail.com

Número celular: 3322232420 / 3325996852

Declaración de consentimiento informado.

Se nos ha explicado con claridad en qué consiste este estudio, además he leído y/o alguien me ha leído el contenido de este consentimiento. Se nos ha dado la oportunidad de hacer preguntas y todas nuestras preguntas han sido contestadas con lenguaje claro y satisfacen plenamente nuestras dudas, además nos han proporcionado una copia de este formato. Al firmar este consentimiento, reconocemos que hemos sido informados sobre los métodos, procedimientos del estudio, la administración del medicamento, así como los beneficios y los efectos adversos que pudieran acontecer. Comprendo que somos libres de retirarnos del estudio en cualquier momento con previo aviso a los investigadores de nuestras razones, sin perder beneficios u obtener penalidad alguna. Libremente y sin reservas damos nuestro consentimiento para participar como paciente en este estudio.

Lugar: Instituto de Investigación en Reumatología y Sistema Músculo Esquelético, Centro Universitario de Ciencias de la Salud, Universidad de Guadalajara. Sierra mojada No. 950. Colonia Independencia. CP. 44370. Tel. (33) 10585200. Ext. 33635.

Guadalajara, Jalisco, a______ de__________ del__________

____________________________________ ______________________ ______________________

Médico o investigador que proporciona la Firma

Fecha

Información y recaba consentimiento.

________________________________ ______________________ ______________________

Nombre Completo del paciente Firma Fecha

TESTIGO 1

Nombre completo del testigo 1:______________________

Dirección: ____

Relación o
Parentesco____

Fecha___________________ Firma___________________

TESTIGO 2

Nombre completo del testigo 2:______________________

Dirección: ____

Relación o
Parentesco____

Fecha___________________ Firma___________________

22.2 ANEXO 2. HOJA DE IDENTIFICACIÓN Y RECOLECCIÓN DE DATOS.

Visita 1 (día -1)

HISTORIA CLÍNICA

DATOS GENERALES		
01	**FECHA DE ELABORACIÓN**	
02	**NÚMERO DE REGISTRO**	
03	**NOMBRE COMPLETO**	
04	**FECHA DE NACIMIENTO**	
05	**EDAD ACTUAL**	
06	**SEXO**	
07	**ESTADO CIVIL**	
08	**OCUPACIÓN**	
09	**ESCOLARIDAD**	
10	**LUGAR DE NACIMIENTO**	
11	**POBLACIÓN ACTUAL**	
12	**COLONIA**	
13	**CALLE**	
14	**NUMERO EXTERIOR**	
15	**NÚMERO INTERIOR**	
16	**CRUZA CON**	
17	**TELEFONO FIJO**	
18	**TELEFONO CELULAR**	
19	**CORREO ELECTRONICO**	

20 ¿COMO CONSIDERA SU ESTADO ACTUAL DE SALUD? : BUENO ( ) REGULAR ( ) MALO ( )

ANTECEDENTES HEREDO FAMILIARES marcar en el paréntesis solo si presenta la enfermedad.		
21	OBESIDAD / SOBREPESO	Mamá( ) Papá( ) Hermanos ( ) Hermanas( ) Tíos p( )( ) Tíos m( )( ) Abuela p ( ) Abuelo p ( ) abuela m ( ) abuelo p ( )<br>Mamá( ) Papá( ) Hermanos ( ) Hermanas( ) Tíos p( )( ) Tíos m( ) ( ) Abuela p ( ) Abuelo p ( ) abuela m( ) abuelo p( )
22	DM2	Mamá( ) Papá( ) Hermanos ( ) Hermanas( ) Tíos p( )( ) Tíos m( )( ) Abuela p ( ) Abuelo p ( ) abuela m ( )abuelo p ( )
23	HTA	Mamá( ) Papá( ) Hermanos ( ) Hermanas( ) Tíos p( )( ) Tíos m( )( ) Abuela p ( ) Abuelo p ( ) abuela m ( ) abuelo p( )
24	ECV *	Mamá( ) Papá( ) Hermanos ( ) Hermanas( ) Tíos p( )( ) Tíos m( )( ) Abuela p ( ) Abuelo p ( ) abuela m ( ) abuelo p( )
25	IAM	Mamá( ) Papá( ) Hermanos ( ) Hermanas( ) Tíos p( )( ) Tíos m( )( ) Abuela p ( ) Abuelo p ( ) abuela m ( ) abuelo p ( )
26	IRC	Mamá( ) Papá( ) Hermanos ( ) Hermanas( ) Tíos p( )( ) Tíos m( )( ) Abuela p ( ) Abuelo p ( ) abuela m ( ) abuelo p ( )
27	EVC	Mamá( ) Papá( ) Hermanos ( ) Hermanas( ) Tíos p( )( ) Tíos m( )( ) Abuela p ( ) Abuelo p ( ) abuela m ( ) abuelo p ( )
28	CANCER	Mamá( ) Papá( ) Hermanos ( ) Hermanas( ) Tíos p( )( ) Tíos m( )( ) Abuela p ( ) Abuelo p ( ) abuela m ( ) abuelo p ( )
29	HIPERCOLESTEROLEMIA	Mamá( ) Papá( ) Hermanos ( ) Hermanas( ) Tíos p( )( ) Tíos m( )( ) Abuela p ( ) Abuelo p ( ) abuela m ( ) abuelo p ( )

***Incluye aterosclerosis, angina de pecho. Insuficiencia venosa y arterial. p. Paterno. m. Materno**

Tíos el primer paréntesis es para tíos y el segundo para tías

ANTECEDENTES PERSONALES PATOLÓGICOS		
30	Consumo de medicamentos	
31	TABAQUISMO	No. De cigarrillos: TIEMPO DE CONSUMO:<br>Fumador pasivo:
32	ALCOHOLISMO	FRECUENCIA: CANTIDAD: TIEMPO DE CONSUMO:
33	TOXICOMANIAS	
34	OBESIDAD/SOBREPESO	
35	DM2	
36	HTA	
37	ECV*	
38	IAM	
39	IRC	
40	EVC	
41	CANCER	
42	HIPERCOLESTEROLEMIA	
43	ANT OBSTÉTRICOS	G: P: C: A:
44	ANT GINECOLÓGICOS	Menarca: Ritmo: FUM: CONSUMO DE HORMONAS:
45	FECHA DE DIAGNOSTICO DE AR	
46	INICIO DE LA ENFERMEDAD	
47	TRATAMIENTO ACTUAL	

48. INTERROGATORIO POR APARATOS Y SISTEMAS: De ser positivo encierre en un ovalo

Cefalea – Vértigo/mareo – Tinitus – visión borrosa – parestesias - sincope – convulsiones-sangrado/hematomas - palidez de piel – uñas frágiles, alopecia - prurito - debilidad muscular – fatiga – dolor articular/muscular/óseo- rigidez articular – edema articular – anorexia - fiebre – sudoración nocturna – - ganancia/perdida ponderal – polidipsia - polifagia – poliuria – tremor – palpitaciones – deprimido- estrés - aletargado- dolor torácico – disnea de esfuerzo – disnea paroxística nocturna – ortopnea – edema de mp`s mt`s – claudicación intermitente/reposo – tos – esputo – hemoptisis – dolor abdominal- distensión abdominal – dispepsia – dolor epigástrico - tenesmo/urgencia – goteo posmicción- disminución del calibre del chorro – incontinencia urinaria – disuria – hematuria – nicturia – cambio en habito intestinal/cambio del calibre – dolor pélvico- prurito vaginal – desecho

49. Descripción:

EXPLORACIÓN FÍSICA

50	TA (mmHg)	BD: BI: B: 2: 3:<br>PROMEDIO:
51	PULSO (X’)	
52	FC (X’)	
53	FR (X’)	
54	TEMPERATURA (°C)	
55	CABEZA	Negativo ( ) Positivo:
56	FONDOSCOPIA	Negativo ( ) Positivo:
57	OTOSCOPIA	Negativo ( ) Positivo:
58	CUELLO	Negativo ( ) Positivo:
59	TORAX	Negativo ( ) Positivo:
60	ÁREA CARDIACA	Negativo ( ) Positivo:
61	ABDOMEN	Negativo ( ) Positivo:

62	EXTREMIDADES	Negativo ( ) Positivo:
63	SNC	Negativo ( ) Positivo:

**ANTROPOMETRÍA E IMPEDANCIA BIOELECTRICA**		
64	PESO (Kg)	
65	IMC (Kg/m2)	
66	MASA GRASA (Kg)	
67	GRASA CORPORAL (%)	
68	AGUA CORPORAL TOTAL (Kg)	
69	MASA MAGRA (Kg)	
70	MASA OSEA	
71	MASA GRASA (Kg/%) en:	
72	TRONCO	
73	BRAZO DERECHO	
74	BRAZO IZQUIERDO	
75	PIERNA DERECHA	
76	PIERNA IZQUIERDA	
**ANTROPOMETRÍA**		
77	TALLA (cm)	
78	CIRCUNFERENCIA DE CINTURA (cm)	
79	CIRCUNFERENCIA DE CADERA (cm)	
80	CIRCUNFERENCIA DE MEDIO BRAZO (cm)	
81	PANICULO ADIPOSO ABDOMINAL (mm)	
82	PANICULO ADIPOSO BICIPITAL (mm)	
83	PANICULO ADIPOSO TRICIPITAL (mm)	

84	PANICULO ADIPOSOSUB-ESCAPULAR (mm)	
85	PANICULO ADIPOSO SUPRA-ILIACO (mm)	
86	PANICULO ADIPOSO FEMORAL (mm)	
87	DIAMETRO SAGITAL (mm)	
88	DIAMETRO CORONAL (mm)	

FRECUENCIA DE CONSUMO DE ALIMENTOS

Indicar con una cruz en la columna de frecuencia, la opción que se considere mas cercana a su realidad. En la columna de la derecha, registrar el número correspondiente a la frecuencia de consumo reportada.

Grupo de alimentos		Consumo (veces al mes)										
		90	60	30	20	16	12	8	4	2	0	
Verduras	Naturales											
	Procesadas											
Frutas	Naturales											
	Procesadas											
Cereales y Tubérculos	Naturales											
	Procesadas											
Leguminosas	Naturales											
	Procesadas											
Origen animal	Naturales											
	Procesadas											
Leche / Yogurt	Naturales											
	Procesadas											
Aceites y grasas	Naturales											
	Procesadas											
Azucares												
Productos chatarra												
Bebidas alcohólicas												

EVALUACIÓN DE LA ACTIVIDAD DE LA ENFERMEDAD

DAS-28

Fecha de evaluación: ______________

Folio del Paciente: ____

No. De Expediente: ________________ No. AR: __________________

Marqué con una X la articulación que corresponda:

	Derecho		Izquierdo	
	**NAD**	**NAT**	**NAD**	**NAT**
Hombros				
Codos				
Carpos				
MTCF1				
MTCF2				
MTCF3				
MTCF4				
MTCF5				
Gaeslen				
IFP1				
IFP2				
IFP3				
IFP4				
IFP5				
Rodillas				
**Total DAS**	**NADt**		**NATt**	

1. Severidad de la enfermedad percibida por el paciente

Muy bien Muy mal

Tamaño total de la recta (mm):________ Total hasta la marca (mm): _________

2. Severidad del dolor percibido por el paciente

Muy bien Muy mal

Tamaño total de la recta (mm):________ Total hasta la marca (mm): _________

3. Severidad percibida por el médico

Muy bien Muy mal

Tamaño total de la recta (mm):________ Total hasta la marca (mm): _________

Cálculo DAS28	
VSG	
PCR	
NAT	
NAD	
Índice NAT/NAD	
DAS28 VSG	
DAS28 PCR	
CDAI	
SDAI	

HEALTH ASSESSMENT QUESTIONNAIRE (HAQ)

**Puntuación:**					
	**Durante la última semana, ¿Ha sido usted capaz de…**	**Sin dificultad**	**Alguna dificultad**	**Mucha dificultad**	**Incapaz de hacerlo**
**1**	**Vestirse solo, incluyendo abrocharse los botones y atarse los cordones de los zapatos?**				
	**Enjabonarse la cabeza?**				
**2**	**Levantarse de una silla sin brazos?**				
	**Acostarse y levantarse de la cama?**				
**3**	**Cortar un filete de carne?**				
	**Abrir un cartón de leche nuevo?**				
	**Servirse la bebida?**				
**4**	**Caminar fuera de casa por un terreno plano?**				
	**Subir cinco escalones?**				
**5**	**Lavarse y secarse todo el cuerpo?**				
	**Sentarse y levantarse del retrete?**				
	**Ducharse?**				
**6**	**Tomar un paquete de azúcar de 1Kg de una estantería colocada por encima de su cabeza?**				
	**Agacharse y recoger ropa del suelo?**				
**7**	**Abrir la puerta de un coche?**				
	**Abrir tarros cerrados que ya antes habían sido abiertos?**				
	**Abrir y cerrar grifos?**				
**8**	**Hacer los mandados y las compras?**				
	**Entrar y salir de un coche?**				
	**Hacer tareas de casa como barrer o lavar los platos?**				

Señale para que actividades necesita la ayuda de otra persona:	Señale si utiliza alguno de estos utensilios habitualmente:

__Vestirse, asearse<br>__Levantarse<br>__Comer<br>__Caminar, pasear<br>__Higiene personal<br>__Alcanzar<br>__Abrir y cerrar cosas<br>__Recados y tareas de casa	__Cubiertos de mango ancho<br>__Bastón, muletas, andador o silla de ruedas<br>__Asiento o barra especial para el baño<br>__Asiento alto para el retrete<br>__Abridor para tarros previamente abiertos

H O J A   D E   R E C O L E C C I Ó N   D E   D A T O S

V i s i t a   2   (d í a   0)

I N I C I O   D E   I N T E R V E N C I Ó N

<table>
<tr><td colspan="2">**Fecha:** I______I_______I_______I
__________________________   **Iniciales:**</td><td>**Aleatorización:**
I______________I
Registro:
I_____I_____I_____I</td></tr>
<tr><td>**Datos clínicos:**

Peso: I_________I **kg**
Talla: I_________I **m**
IMC: I__________I **kg/m²**
FC: I___________I **lpm**
FR: I___________I **rpm**</td><td colspan="2">**Datos paraclínicos:**
TA (mmHg): I________I / I________I
PAC (mmHg): I________I / I________I
Porcentaje de grasa Corporal: I_______I **%**
Porcentaje de grasa Visceral: I_______I **%**
Circunferencia de Cintura: I_______I **cm**
Circunferencia Abdominal: I_______I **cm**</td></tr>
</table>

**Comentarios adicionales:**
____
____
____
____
____
____

Fecha para visita 3:___________________

V i s i t a   3   (d í a   3 0 ± 2)

<table>
<tr><td colspan="2">**Fecha:** I_____I_____I_____I   **Iniciales:**
__________________________</td><td>**Aleatorización:** I______________I
Registro: I_____I_____I_____I</td></tr>
<tr><td>**Datos clínicos:**
Peso: I_________I **kg**
Talla: I_________I **m**
IMC: I__________I **kg/m²**
Grasa Corporal: I__________I %
Circunferencia abdominal: I__________I cm</td><td colspan="2">**Datos clínicos:**
FC: I___________I **lpm**
FR: I___________I **rpm**
TA (mmHg): I________I / I________I
Circunferencia cintura: I__________I cm</td></tr>
</table>

**Apego al tratamiento:**	**Tolerabilidad y efectos adversos:**
**Fecha de la primera dosis:_________________________**<br>**Fecha de la última dosis:**<br>**___________________________**<br>**Ha olvidado alguna Dosificación:**<br>**____________________**<br>**¿Cuándo?**<br>**____**<br>**Recuento de medicamento:**<br>**_________________________**	**Duración y descripción del evento adverso: _________________**<br>**____**<br>**____**<br>**____**<br>**____**

Fecha para visita 4:___________________

V i s i t a 4 (d í a 6 0 ± 2)

**Fecha: l_____l_____l_____l** **Iniciales:** **___________________________**	**Aleatorización: l______________l**<br>**Registro: l_____l_____l_____l**
**Datos clínicos:**<br>**Peso: l_________l kg**<br>**Talla: l_________l m**<br>**IMC: l__________l kg/m^2**<br>**Grasa Corporal: l__________l** %<br>**Circunferencia abdominal: l__________l** cm	**Datos clínicos:**<br>**FC: l___________l lpm**<br>**FR: l___________l rpm**<br>**TA (mmHg): l________l / l________l**<br>**Circunferencia cintura: l__________l** cm
**Apego al tratamiento:**<br><br>**Fecha de la primera dosis:_________________________**<br>**Fecha de la última dosis:**<br>**__________________________**<br>**Ha olvidado alguna Dosificación:**<br>**___________________**<br>**¿Cuándo?**<br>**____**<br>**Recuento de medicamento:**<br>**________________________**	**Tolerabilidad y efectos adversos:**<br><br>**Duración y descripción del evento adverso: _________________**<br>**____**<br>**____**<br>**____**<br>**____**

V i s i t a   5   (d í a   9 0 ± 2)

**Fecha: I_____I_____I_____I** **Iniciales:** ________________________	**Aleatorización:** I_____I_____I_____I **Registro: I_____I_____I_____I**
**Datos clínicos:**	**Datos paraclínicos:**
**Peso: I_________I kg**	**Ac. Urico:_____________mg/dl**
**Talla: I_________I m**	**Creatinina:____________ mg/dl**
**IMC: I__________I kg/m^2**	**TGO:________________U/l**
**FC: I___________I lpm**	**TGP:________________ U/l**
**FR: I___________I rpm**	**VOP________________**
**TA (mmHg): I________I / I________I**	
**PAC (mmHg): I________I / I________I**	
**Porcentaje de grasa Corporal: I_______I %**	
**Porcentaje de grasa Visceral: I_______I %**	
**Circunferencia de Cintura: I_______I cm**	
**Glucosa basal: I__________I mg/dl**	
**C-HDL: I__________I mg/dl**	
**C-LDL: I__________I mg/d**	
**CT: I__________I mg/dl**	
**Triglicéridos: I__________I mg/dl**	
**Comentarios adicionales:** ____	

22.3 ANEXO 3. DIARIO DE MONITOREO Y SEGUIMIENTO DEL PACIENTE.

PROYECTO DE INVESTIGACIÓN:

Efecto del uso de enalapril en la rigidez arterial en pacientes con artritis reumatoide.

<table>
<tr><td>Nombre del paciente:</td><td>Aleatorización: I_____I_____I_____I

Registro: I_____I_____I_____I</td></tr>
<tr><td>Fecha de inicio:
I________I________I________I</td><td>Fecha de término:
I________I________I________I</td></tr>
<tr><td colspan="2"><u>Investigador responsable:</u>

Dra. Mónica Vázquez del Mercado Espinosa / Dr. Felipe de Jesús Pérez Vázquez
<u>3322232420 / 3325996852</u></td></tr>
</table>

RECUERDE:

1.- Llevar ropa ligera para la toma de peso y medidas.

2- Llevar su diario de tratamiento a sus citas.

3.- Llevar el frasco de cápsulas que se le entrego para cambiarlo por otro con nuevo tratamiento

¿CÓMO LLENAR EL DIARIO?

- **REGISTRO DE CITAS:**
- Aquí se registrarán las fechas en que acudirá a sus evaluaciones.
  -
- **REGISTRO DE TOMA DEL TRATAMIENTO:**
  - Usted deberá registrar las fechas y horas de la toma de cápsula, que se indican antes del desayuno cada día.

- **OBSERVACIONES O COMENTARIOS:**
  - Cualquier molestia relacionado con la ingesta de medicamento, favor de reportarlo con los médicos responsables.
  - Usted debe registrar cualquier **molestia** o bien **enfermedad** y/o **tratamiento** que se presente durante el periodo del estudio, incluyendo las que usted crea que no se relacionen con el medicamento. También registrará si fue necesaria la toma de otro medicamento además del de estudio.
  - En este espacio usted anotará los cambios en el estado de salud o bien si tuvo algún evento extraordinario ya sea de actividad física o dieta.

Registro de citas

Visita	Fecha	Hora	Indicaciones
**1 Inicio**		8 am	Ayuno
**2 seguimiento**		8 am	Traer su frasco<br>Traer su diario
**3 seguimiento**		8 am	Traer su frasco<br>Traer su diario
**4 fin del estudio**		8 am	Ayuno<br>Traer su diario
**5 resultados**		8 am	

Registro semanal

Fecha ___/___/____ al ___/___/____

# cápsulas entregadas en el mes: _________

Semana del ___/___/____ al ___/___/____

Registro de la toma antes de las comidas

**Frasco**	**Día**	**MARQUE**		✓	×	**Observaciones/ Comentarios***
		Antes del desayuno		Antes de la cena		
___	1					
___	2					
___	3					
___	4					
___	5					

___	6				
___	7				
___	8				

#cápsulas que olvidó tomar: ________

Registro de toma de cápsulas

Fecha ___/___/____ al ___/___/____ # cápsulas entregadas en el mes: _________

Semana del ___/___/____ al ___/___/____

Registro de la toma antes de las comidas

**Frasco**	**Día**	**MARQUE**		✓ ×	**Observaciones/ Comentarios***
		Antes del desayuno		Antes de la cena	
___	9				
___	10				
___	11				
___	12				
___	13				
___	14				
___	15				
___	16				

#cápsulas que olvidó tomar: ________

Registro de toma de cápsulas

Fecha ___/___/____ al ___/___/____ # cápsulas entregadas en el mes: _________

Semana del ___/___/____ al ___/___/____

Registro de la toma antes de las comidas

Frasco	Día	MARQUE		✓ ✗	Observaciones/ Comentarios*
		Antes del desayuno		Antes de la cena	
___	17				
___	18				
___	19				
___	20				
___	21				
___	22				
___	23				
___	24				

#cápsulas que olvidó tomar: _________

Registro de toma de cápsulas

Fecha ___/___/____ al ___/___/____ # cápsulas entregadas en el mes: _________

Semana del ___/___/____ al ___/___/____

Registro de la toma antes de las comidas

Frasco	Día	MARQUE		✓ ✗	Observaciones/ Comentarios*
		Antes del desayuno		Antes de la cena	
___	25				
___	26				

___	27				
___	28				
___	29				
___	30				

#cápsulas que olvidó tomar: _________

Comentarios:
