# Supplementary material for: Enalapril Influence on Arterial Stiffness in Rheumatoid Arthritis Women: A Randomized Clinical Trial
Source: Front Med (Lausanne). 2020 Jan 29;6:341. doi: 10.3389/fmed.2019.00341 (PMC7025600; doi:10.3389/fmed.2019.00341)
Supplement: Supplementary file 3 [file Table_1.docx]

**Supplementary Table 1.** Delta of clinical characteristics of RA study groups at Baseline and after 12 weeks of intervention.

|  | | | |
| --- | --- | --- | --- |
|  | **Delta** | | |
|  | **Placebo**  **n = 26** | **Enalapril**  **n = 27** | ***P*** |
| **∆ Age, years** | 0.26  (0.25 to 0.29) | 0.26  (0.24 to 0.28) | 0.337 |
| **∆ BMI, kg/m^2^** | -0.06 *±* 0.740 | -0.09 *±* 0.931 | 0.881 |
| **∆ Fat mass, %** | -0.27 *±* 2.405 | -0.68 *±* 3.050 | 0.592 |
| **∆ TC, mg/dL** | 8.00  (-22.50 to 15.00) | 5.00  (-5.00 to 30.00) | 0.197 |
| **∆ TG, mg/dL** | 9.19 *±* 48.801 | 7.70 *±* 68.435 | 0.928 |
| **∆ HDL-c, mg/dL** | -1.35 *±* 9.334 | 1.67 *±* 7.206 | 0.193 |
| **∆ LDL-c, mg/dL** | -38.65 *±* 58.460 | -11.07 *±* 51.099 | 0.073 |
| **∆ Glucose, mg/dL** | -5.92 *±* 11.610 | -2.11 *±* 13.648 | 0.279 |
| **∆ Disease duration, years** | 0.26  (0.25 to 0.29) | 0.26  (0.24 to 0.28) | 0.337 |
| **∆ CRP, mg/L** | -0.16  (-2.82 to 2.57) | -1.00  (-3.60 to 2.20) | 0.796 |
| **∆ ESR, mm/h** | 0.65 *±* 10.789 | -1.59 *±* 11.157 | 0.460 |
| **∆ DAS-28 CRP** | -0.19  (-1.31 to 0.45) | 0.00  (-0.55 to 0.96) | 0.173 |
| **∆ CDAI** | -2.65  (-14.77 to 1.77) | 1.60  (-2.80 to 5.20) | 0.102 |
| **∆ SDAI** | -1.90  (-12.85 to 4.32) | 0.70  (-10.60 to 6.70) | 0.346 |
| **∆ QRISK3-2018, %** | 0.00  (-0.15 to 0.20) | -0.10  (-0.40 to 0.10) | 0.159 |
| **∆ QRISK3-2018, RR** | -0.01 *±* 0.190 | -0.07 *±* 0.206 | 0.294 |
| **∆ pSBP, mmHg** | -2.85 *±* 6.035 | -6.00 *±* 8.131 | 0.117 |
| **∆ pDBP, mmHg** | -1.08 *±* 4.459 | -2.07 *±* 6.749 | 0.530 |
| **∆ cIMTmean, µm** | 7.52 *±* 88.863 | 1.35 *±* 72.493 | 0.783 |
| **∆ cDistensibility, 10^-3^/ kPa** | 0.07 *±* 8.908 | 2.53 *±* 8.143 | 0.298 |
| **∆ Einc, kPax10^3^** | 0.01 *±* 0.205 | -0.08 *±* 0.181 | 0.103 |
| **∆ cfPWV, m/s** | 0.05 *±* 0.922 | -0.04 *±* 0.929 | 0.754 |
| **∆ CAVI** | 0.39 *±* 0.944 | -0.21 *±* 0.888 | 0.032 |

∆ **BMI,** Delta Body mass index; ∆ **TC**, Delta total cholesterol; ∆ **TG**, Delta triglycerides; ∆ **HDL-c**, Delta High density lipoprotein cholesterol; ∆ **LDL-c**, Delta Low-density lipoprotein cholesterol; ∆ **CRP**, Delta c-reactive protein; ∆ **ESR**, Delta Erythrocyte sedimentation rate; ∆ **CDAI,** Delta Clinical disease activity index; ∆ **SDAI,** Delta Simple disease activity index; ∆ **DAS-28**, Delta Disease activity score on 28 joints; ∆ **QRISK3-2018%**, Delta 10-year QRISK3 score; ∆ **QRISK3-2018RR**, Delta QRISK3 relative risk. ∆ **pSBP,** Delta peripheral systolic blood pressure**;** ∆ **pDBP,** Delta peripheral diastolic blood pressure; ∆ **cIMT,** Delta Carotid intima-media thickness; ∆ **cDistensibility**, Delta Carotid artery distensibility; ∆ **Einc,** Delta Young´s Elastic modulus; ∆ **cfPWV,** Delta carotid to femoral pulse wave velocity**;** ∆ **CAVI,** Delta Cardio-Ankle Vascular Index. Values were representend as mean ± SD and median with ranges.
