# Supplementary material for: Enalapril Influence on Arterial Stiffness in Rheumatoid Arthritis Women: A Randomized Clinical Trial
Source: Front Med (Lausanne). 2020 Jan 29;6:341. doi: 10.3389/fmed.2019.00341 (PMC7025600; doi:10.3389/fmed.2019.00341)
Supplement: Supplementary file 4 [file Table_2.docx]

| **Supplementary Table 2.** CAVI correlations in the RA enalapril intervention group. | | | | | | |
| --- | --- | --- | --- | --- | --- | --- |
|  | **Baseline** | | **After 12 Weeks** | | **∆** | |
|  |  | **CAVI*** |  | **CAVI**** |  | **CAVI***** |
| **Age, years** |  | 0.650  (0.001) |  | 0.718  (0.000) |  | 0.253  (0.243) |
| **BMI, kg/m^2^** |  | 0.138  (0.531) |  | -0.016  (0.942) |  | 0.019  (0.933) |
| **Fat mass, %** |  | 0.134  (0.542) |  | 0.005  (0.980) |  | 0.181  (0.408) |
| **TC, mg/dL** |  | -0.090  (0.682) |  | -0.019  (0.930) |  | -0.264  (0.223) |
| **TG, mg/dL** |  | -0.090  (0.684) |  | -0.171  (0.436) |  | 0.140  (0.523) |
| **HDL-c, mg/dL** |  | -0.132  (0.548) |  | 0.076  (0.729) |  | -0.407  (0.054) |
| **LDL-c, mg/dL** |  | -0.002  (0.994) |  | 0.148  (0.501) |  | -0.068  (0.758) |
| **Glucose, mg/dL** |  | 0.277  (0.201) |  | -0.048  (0.827) |  | 0.259  (0.233) |
| **Disease duration, years** |  | 0.218  (0.318) |  | 0.471  (0.023) |  | 0.253  (0.243) |
| **RF (+), n (%)** |  | -0.235  (0.280) |  | -0.055  (0.803) |  | -0.100  (0.651) |
| αCCP positive **(+), n (%)** |  | -0.125  (0.589) |  | -0.088  (0.737) |  | -0.244  (0.329) |
| αCCP log_10_ |  | -0.223  (0.332) |  | -0.008  (0.971) |  | 0.368  (0.101) |
| **CRP, mg/L** |  | 0.146  (0.507) |  | -0.209  (0.338) |  | 0.227  (0.297) |
| **ESR, mm/h** |  | 0.071  (0.748) |  | 0.133  (0.545) |  | -0.269  (0.214) |
| **DAS-28 CRP** |  | 0.118  (0.591) |  | 0.087  (0.692) |  | -0.302  (0.161) |
| **CDAI** |  | 0.111  (0.613) |  | 0.000  (0.998) |  | -0.316  (0.141) |
| **SDAI** |  | 0.214  (0.328) |  | -0.029  (0.897) |  | -0.084  (0.703) |
| **pSBP, mmHg** |  | 0.591  (0.003) |  | 0.333  (0.121) |  | 0.003  (0.988) |
| **pDBP, mmHg** |  | 0.378  (0.075) |  | 0.076  (0.730) |  | 0.293  (0.175) |
| **Methotrexate, mg** |  | 0.139  (0.560) |  | 0.045  (0.852) |  | -0.121  (0.610) |
| **Sulfasalazine, gr** |  | 0.088  (0.696) |  | 0.107  (0.635) |  | -0.033  (0.886) |
| **Cloroquine, mg** |  | -0.079  (0.719) |  | -0.106  (0.631) |  | 0.060  (0.787) |

**CAVI,** Cardio-Ankle Vascular Index**; BMI,** Body mass index; **TC**, total cholesterol; **TG**, triglycerides; **HDL-c**, High density lipoprotein cholesterol; **LDL-c**, Low-density lipoprotein cholesterol; **RF**, rheumatoid factor; **aCCP**, anti-cyclyc citrullinated peptide antibodies; **CRP**, c-reactive protein; **ESR**, Erythrocyte sedimentation rate; **CDAI,** Clinical disease activity index; **SDAI,** Simple disease activity index; **DAS-28**, Disease activity score on 28 joints; **QRISK3-2018%**, 10-year QRISK3 score; **QRISK3-2018RR**, QRISK3 relative risk. **pSBP,** peripheral systolic blood pressure**; pDBP,** peripheral diastolic blood pressure. *Correlation with clinical parameters at baseline, **Correlation with clinical parameters after 12 weeks of intervention, ***Correlation with delta of clinical parameters.
